# Linocin M18 protein from the insect pathogenic bacterium *Brevibacillus laterosporus* isolates

**DOI:** 10.1007/s00253-023-12563-8

**Published:** 2023-05-19

**Authors:** Tauseef K. Babar, Travis R. Glare, John G. Hampton, Mark R. H. Hurst, Josefina Narciso, Campbell R. Sheen, Barbara Koch

**Affiliations:** 1 Bio-Protection Research Centre, Lincoln University, Lincoln, Canterbury, 7647 New Zealand; 2 Department of Entomology, Faculty of Agricultural Sciences and Technology, Bahauddin Zakariya University, Multan, 60000 Pakistan; 3 Faculty of Agriculture and Life Sciences, Lincoln University, Lincoln, Canterbury, 7647 New Zealand; 4 Resilient Agriculture, AgResearch, Lincoln Research Centre, Christchurch, New Zealand; 5 Protein Science and Engineering, Callaghan Innovation, Christchurch, New Zealand

**Keywords:** Antibacterial protein, *Brevibacillus laterosporus*, Encapsulating protein, Heterologous expression, Insect pathogenic bacterium, Linocin M18 protein

## Abstract

**Abstract:**

*Brevibacillus laterosporus* (*Bl*) is a Gram-positive and spore-forming bacterium. Insect pathogenic strains have been characterised in New Zealand, and two isolates, *Bl* 1821L and *Bl* 1951, are under development for use in biopesticides. However, growth in culture is sometimes disrupted, affecting mass production. Based on previous work, it was hypothesised that Tectiviridae phages might be implicated. While investigating the cause of the disrupted growth, electron micrographs of crude lysates showed structural components of putative phages including capsid and tail-like structures. Sucrose density gradient purification yielded a putative self-killing protein of ~30 kDa. N-terminal sequencing of the ~30 kDa protein identified matches to a predicted 25 kDa hypothetical and a 31.4 kDa putative encapsulating protein homologs, with the genes encoding each protein adjacent in the genomes. BLASTp analysis of the homologs of 31.4 kDa amino acid sequences shared 98.6% amino acid identity to the Linocin M18 bacteriocin family protein of *Brevibacterium* sp. JNUCC-42. Bioinformatic tools including AMPA and CellPPD defined that the bactericidal potential originated from a putative encapsulating protein. Antagonistic activity of the ~30 kDa encapsulating protein of *Bl* 1821L and *Bl* 1951during growth in broth exhibited bacterial autolytic activity. LIVE/DEAD staining of *Bl* 1821L cells after treatment with the ~30 kDa encapsulating protein of *Bl* 1821L substantiated the findings by showing 58.8% cells with the compromised cell membranes as compared to 37.5% cells in the control. Furthermore, antibacterial activity of the identified proteins of *Bl* 1821L was validated through gene expression in a Gram-positive bacterium *Bacillus subtilis* WB800N.

**Key Points:**

• *Gene encoding the 31.4 kDa antibacterial Linocin M18 protein was identified*

• *It defined the autocidal activity of Linocin M18 (encapsulating) protein*

• *Identified the possible killing mechanism of the encapsulins*

**Supplementary Information:**

The online version contains supplementary material available at 10.1007/s00253-023-12563-8.

## Introduction

*Brevibacillus laterosporus* (*Bl*) is a Gram-positive and spore-forming bacterium belonging to the *Brevibacillus brevis* phylogenetic cluster (Shida et al. 1996). Numerous strains exhibit pathogenicity against insect pests of different orders including Coleoptera  (Kouadio et al. 2021; Salama et al. 2004), Diptera (Bedini et al. 2020; Carramaschi et al. 2015), Lepidoptera  (van Zijll de Jong et al. 2016), and also against nematodes (Hamze and Ruiu 2022) and molluscs (De Oliveira et al. 2004) have been documented. Due to the entomocidal action of *Bl* against multifarious insect pests, there has been a surge of interest in commercialisation for future microbial pesticide development (Baum et al. 2020; Boets et al. 2011; Floris et al. 2008; Glare et al. 2014; Glare et al. 2018; Sampson et al. 2016). In New Zealand, three insect pathogenic strains (*Bl* 1821L, *Bl* 1951, *Bl* Rsp) demonstrating activity against diamondback moth and mosquitoes (van Zijll de Jong et al. 2016) have been characterised and found to be genetically distinct from the strains of other parts of the world (Glare et al. 2020). Strains *Bl* 1821L and *Bl* 1951 are under development as biopesticides, but due to difficulties in mass production, it has been often problematic to harness their insecticidal potential. Therefore, a study was undertaken to find the cause of stunted growth of these two isolates.

Prokaryotes including bacteria often harbour nanocompartments within the cytoplasm (Wiryaman and Toor 2022). These protein-based compartments play key roles in various metabolic and ecological processes ranging from iron homeostasis to carbon fixation (Giessen et al. 2019; McDowell and Hoiczyk 2022). One such class is the encapsulins whose shell proteins seize a fold previously found only in viral capsids (Suhanovsky and Teschke 2015; Wikoff et al. 2000). Encapsulins were first identified in 1994 as a high molecular-weight (HMW) complex in the culture supernatant of a Gram-positive bacterium *Brevibacterium linens* M18 which exhibited bacteriostatic activity against various strains of *Arthrobacter*, *Bacillus*, *Brevibacterium*, *Corynebacterium*, and *Listeria* while N-terminal sequencing of the antagonistic protein identified the encoding gene, *lin*, in the chromosomal DNA (Eppert et al. 1997; Valdes-Stauber and Scherer 1996). Since then, encapsulins have been described in various bacteria including *Mycobacterium leprae* (Winter et al. 1995), *M. tuberculosis* (Rosenkrands et al. 1998), *Thermotoga maritima* (Hicks et al. 1998), *Streptomyces coelicolor* (Kawamoto et al. 2001), and *Quasibacillus thermotolerans* (Giessen et al. 2019). Furthermore, several authors have highlighted the nature, structural features, and the role of these bacterial nanocompartments (Almeida et al. 2021; Andreas and Giessen 2021; Chmelyuk et al. 2023; Giessen 2022; Giessen and Silver 2016; Giessen and Silver 2017; Jones and Giessen 2021; McDowell and Hoiczyk 2022; Wiryaman and Toor 2022).

Herein, we report on the isolation, purification, and characterisation of Linocin M18, a putative antibacterial (encapsulating) protein of the insect pathogenic strains *Bl* 1821L and *Bl* 1951.

## Material and methods

### Bacterial strains and growth conditions

The insect pathogenic strains *Bl* 1821L and *Bl* 1951, originally isolated from *Brassica* seeds (van Zijll de Jong et al. 2016) and held at −80 °C in the Bio-Protection Research Centre Culture Collection, Lincoln University, New Zealand, were used in this study. The strains were routinely grown in Luria-Bertani medium broth (LB Miller, Sigma, St. Louis, MI, USA) on an orbital shaker (Conco, TU 4540, Taibei, Taiwan) at 250 rpm and 30 °C overnight for further usage.

### Mitomycin C induction of putative antibacterial proteins (ABPs)

To induce the putative ABPs residing in the genome of *Bl* 1821L and *Bl* 1951, a modified protocol of Rybakova et al. (2014) was used. Single colonies of *Bl* 1821L/*Bl* 1951 were used to inoculate 5 mL of LB broth. The inoculated culture was incubated overnight at 30 °C with shaking on rotatory shaker (Conco, TU 4540, Taiwan) at 250 rpm. Aliquots (500 μL) of the overnight culture of *Bl* 1821L/*Bl* 1951 were used to inoculate 25 mL of LB broth in 250 mL conical flasks. This culture was allowed to grow at 30 °C with shaking at 250 rpm until the culture attained turbidity (10–12 h). Mitomycin C (Sigma, Sydney, NSW, Australia) was added into the flasks, which were left shaking overnight at 40 rpm and ambient temperature (24 °C). The flasks were monitored for signs of lysis (clearing of the culture or accumulation of bacterial debris). For induction of putative ABPs of *Bl* 1821L, mitomycin C at 1 μg/mL (Babar et al. 2022a) and for *Bl* 1951 at 3 μg/mL (Babar 2021) was used. After induction, the culture was centrifuged at 16,000 × g for 10 min, and the supernatant was passed through a 0.22 μm filter (Merck Millipore, UK) to obtain the cell free supernatants (CFS). Antibacterial activity of the CFS against the *Bl* 1821L and *Bl* 1951 as the host bacterium was determined using the Kirby-Bauer disc diffusion test (Kirby et al. 1956).

### Purification of putative ABPs

The crude lysates of *Bl* 1821L and *Bl* 1951 harbouring the putative ABPs were purified using the sucrose density gradient centrifugation method (Babar et al. 2022b). For the purification, 7.5 mL CFS was transferred into polypropylene centrifuge tubes (Konical, Beckman, Brea, CA, USA) and ultracentrifuged at 151,263 × g in a swing bucket rotor (41Ti, Beckman, Brea, CA, USA) for 70 min. The concentrated pellet was resuspended in 100–150 μL of tris buffer saline (10 × TBS) (40 g NaCl, 1 g KCl, 2.42 g TRIS Base, 16.5 g TRIS-HCl, dH_2_O 490 mL, pH 7.4). Sucrose density gradients (10%, 20%, 30%, 40%, and 50%) were made by applying layers of 1.5 mL of freshly prepared sucrose solution. The highest concentration of sucrose was applied first followed by the lower concentrations. The *Bl* 1821L and *Bl* 1951 ultracentrifuged preparations (200 μL) were applied on top of the sucrose density gradients and centrifuged at 151,263 × g in a swing bucket rotor (41Ti, Beckman) for 70 min. After ultracentrifugation, each gradient was carefully taken out and tested against *Bl* 1821L and *Bl* 1951 as the host bacterium following the Kirby-Bauer disc diffusion assay test. Next, each of the gradients (1.5 mL) was transferred into polypropylene centrifuge tubes to make the final volume of 7.5 mL with the addition of TBS and ultracentrifuged at 151,263 × g for 70 min to concentrate the ABPs. Based on our previous work (Babar et al. 2022b), the purified and concentrated solution of 20% sucrose density gradient of *Bl* 1821L and 50% of *Bl* 1951 were cleaned from sucrose residues using an Amicon Ultra-0.5 (10 kDa) centrifugal filter (Merck Millipore, Cork, Ireland) for sodium dodecyl sulphate polyacrylamide gel electrophoresis (SDS-PAGE) and transmission electron microscopy (TEM).

### TEM analysis of crude and purified lysates

For TEM analysis of crude or purified lysates, 5 μL sample of the concentrated pellet was applied to a freshly glow-discharged plastic-coated hydrophilic 200 mesh EM grid (ProSciTech; Thuringowa, Australia) and stained with 3 μL of 0.7% uranyl acetate (UA, pH 5). The sample was subjected to TEM analysis at 18,000 to 25,000 magnifications using a Morgagni 268D (FEI, Hillsboro, OR, USA) TEM operated at 80 KeV. The images were photographed using the TENGRA camera.

### SDS-PAGE analysis of purified putative ABPs

The highly purified proteins of *Bl* 1821L and *Bl* 1951 were subjected to SDS-PAGE (10%) analysis using the protocol of Laemmli (1970). Ten microlitre of protein ladder (BIO-RAD, Precision Plus Protein™ Standards, Hercules, CA, USA) was loaded. Gels were run for 50 min at 200 volts and then washed four times with H_2_O before staining. For N-terminal sequencing, the protocol of Yasumitsu et al. (2010) involving the RAMA stain (29 mL MQW (milli que water), 12.5 mL CBB (coomassie brilliant blue) stock (1 gm CBB, 300 MeOH, 200 mL MQW), 3.75 mL ammonium sulphate (200 gm, 500 mL MQW), 5 mL of glacial acetic acid per gel) was followed. Prior to overnight destaining in water, the gel was rinsed in water. In addition, the purified and concentrated proteins were also assessed with the silver staining method of Blum et al. (1987).

### N-terminal sequencing and bioinformatic analysis

Purified bands of the protein of interest of *Bl* 1821L and *Bl* 1951 were excised for N-terminal sequence analysis. Peptides generated by trypsin (Promega, Madison, WI, USA) cleavage were analysed using liquid chromatography mass spectrometry (LC-MS). The obtained peptide masses were compared with the National Centre for Biotechnology Information (NCBI) library using the genomes of *Bl* 1821L (GenBank accession NZ_CP033464.1) and *Bl* 1951 (GenBank accession RHPK01000003, contig 1). Amino acid sequences from the genomes were assessed and characterised using UniProt database (https://www.uniprot.org; accessed 25 September 2019), BLASTp (Basic Local Alignment Search Tool) (https://blast.ncbi.nlm.nih.gov; accessed 23 April 2020), ExPasy (https://www.expasy.org; accessed 23 April 2020), and the CLUSTALO (Clustal Omega) (https://www.uniprot.org; accessed 23 April 2020). The identified ~30 kDa putative antibacterial protein of *Bl* 1821L and *Bl* 1951 was analysed using Geneious basic (Kearse et al. 2012). Amino acid sequence alignments were performed using CLUSTALO (https://www.uniprot.org; accessed 23 April 2020). The genomes of both the insect pathogenic strains were searched for the presence of secondary metabolism biosynthesis gene clusters using antiSMASH (antibiotics and secondary metabolite analysis shell) (https://antismash.secondarymetabolites.org; accessed 23 April 2020) and BAGEL4 (identifying the prokaryotic genes involved in the biosynthesis of ribosomally synthesised and post translationally modified peptides (RiPPs) and (unmodified) bacteriocins) (http://bagel4.molgenrug.nl; accessed 23 April 2020) (Medema et al. 2011; van Heel et al. 2018). The antiSMASH-predicted gene clusters were compared with the similar known gene clusters using the programme MIBiG 3.0 (minimum information about a biosynthetic gene cluster) (https://mibig.secondarymetabolites.org; accessed 10 April 2023) (Terlouw et al. 2023). Bioinformatic tools AMPA (http://tcoffee.crg.cat/apps/ampa; accessed 23 April 2020) (Torrent et al. 2012) and CellPPD (Designing of Cell Penetrating Peptides) (http://crdd.osdd.net/raghava/cellppd; accessed 23 April 2020) (Gautam et al. 2013) were used to identify the motifs related to bactericidal activity and potency of cell penetrating peptides (CPPs) of 31.4 kDa putative encapsulating protein (EP) of *Bl* 1821L and *Bl* 1951. AMPA is an automated web server used to predict the antimicrobial regions in a protein. It is based on an antimicrobial propensity scale that takes into account the physical and chemical properties of each amino acid like hydrophobicity and amphipathicity and the relevance of amino acid position for antimicrobial activity (Torrent et al. 2012). In this study, an antimicrobial index (AI) set at a default value of 0.225 was used, and an AI of ˂ 0.225 was considered a positive hit for an antimicrobial peptide (AMP). CellPPD is a support vector machine (SVM) that scores each amino acid residue with an SVM score, and an SVM >0 is considered a positive CPP (cell-penetrating peptide) hit (Gautam et al. 2013).

### Bactericidal activity assay

To determine the bactericidal activity of the putative ABPs of *Bl* 1821L and *Bl* 1951 in crude and purified forms, growth assay was performed. Five hundred microlitre of crude lysate harbouring the putative ABPs was pipetted into 5 mL of the overnight grown culture of the host bacteria. For the control treatment, a similar volume of LB broth/TBS buffer without putative ABPs was used. All the flasks with/without putative ABPs were maintained at 30 °C on a shaking incubator (Conco, TU 4540, Taiwan). Samples were drawn from each treatment after 1, 3, 6, 12, 18, and 24 h of incubation to determine the number of viable cells (CFU/mL) and OD_600nm_. Cell biomass (OD_600nm_) was measured using the Ultrospec-10 spectrophotometer (Amersham Biosciences, Amersham, UK). To determine the number of viable cells (CFU/mL), tenfold serial dilutions (10^−1^ to 10^−6^) of each time interval were prepared, and 100 μL from each dilution was spread in duplicate on two independent LB agar plates. After incubation at 30 °C for 2–3 days, the colonies were counted using a colony counter (Cole-Palmer™ Stuart™, Thermo Fisher Scientific, UK) and converted into log_10_ CFU/mL. To define potential ABP antagonistic activity, the percentage change in number of viable cells compared to the control treatment (without ABPs) after treatment with the putative ABPs was calculated. Four independent sets of experiments with biological replications were performed, and the pooled data were subjected to statistical analysis using the ANOVA (analysis of variance) test through the Genstat 20th edition programme. Similar to this, an experiment with the purified ~30 kDa putative EP of *Bl* 1821L and *Bl* 1951 was performed.

### LIVE/DEAD staining

Ten milliliters of overnight culture of the host bacterium (*Bl* 1821L or *Bl* 1951) was aliquoted into two parts. One part was treated with the ~30 kDa-purified putative EP, and the other part, serving as the control, was treated with the TBS buffer. Two volumes (100 μL and 200 μL) of purified putative EPs (~30 kDa) were used. All the treatments with/without TBS or putative EPs were incubated at 30 °C, and the samples were drawn from each treatment (1, 3, 6, 12, 18, 24 h) for LIVE/DEAD staining. For fluorescent microscopy, 5 μL of SYTO9/propidium iodide (PI) stain (LIVE/DEAD® BacLight™ bacterial viability assay kit; Invitrogen, Carlsbad, CA, USA) was mixed with an equal volume of host bacteria with/without ~30 kDa-purified putative EP for 5 min in a UV-safe tube. Next, 5 μL of this mixture was pipetted onto a slide, and 3 μL of molten agarose (0.1%) was added to reduce the cell movement. Cells were examined under a BX51 fluorescent microscope (Olympus, Sydney, NSW, Australia) at × 100 magnification. An Olympus DP74 camera (Olympus, Sydney, NSW, Australia) attached to the microscope was used to take the images with the help of CellSens 2.1 software (Olympus, Sydney, NSW, Australia). SYTO9 (green) stain was visualised using a FITC filter (Olympus, Sydney, NSW, Australia) with excision of 550 nm wavelength, and for PI stain (red), a wavelength of 650 nm was used. Immediately, both the images were overlapped using the CellSens 2.1 software to visualise the status of cells (alive or dead).

### Expression of putative ABPs  in the Gram-positive bacterium *B. subtilis* WB800N

Genomic DNA of *Bl* 1821L was extracted using the DNeasy Blood and Tissue Kit (250) (QIAGEN, Hilden, Germany) according to the manufacturer’s instructions. Identified *Bl* 1821L genes corresponding to a hypothetical protein (25 kDa) residing at 18,917 bp to 19,564 bp (648 bp), a putative EP (31.4 kDa) encoded at 19,592 bp to 20,434 bp (843 bp), and both the genes encoding 25 kDa and 31.4 kDa proteins (Supplementary (S) Information, Figure S1) were cloned (pHT01) into a Gram-positive bacterium *Bs* WB800N expression system. Three constructs were made, pHT01-*hypo* (25 kDa), pHT01-*encap* (31.4 kDa), and pHT01-*hypo.encap* containing both the genes. To form these, vector primers (Table 1.) were used to amplify each region and their combinations using CloneAmp (TakaRa Bio, San Jose, CA, USA) master mix according to the manufacturer’s instructions. The bands of appropriate size were visualised and excised on a DarkReader blue light table (Clare Chemical Research, Dolores, CO, USA) before purification using a NucleoSpin gel and PCR purification kit (Macherey-Nagel, Allentown, PA, USA) according to the manufacturer’s instructions.

**Table 1.**
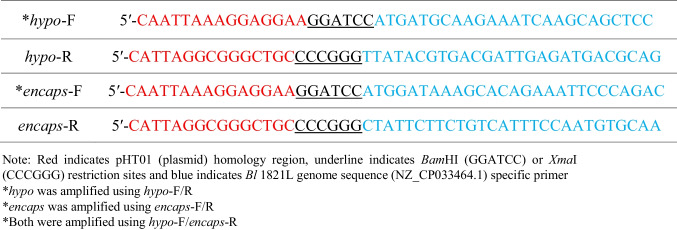
Primers used for amplification of a hypothetical- *hypo (*25 kDa), putative encapsulating gene- *encaps *(31.4 kDa), and both the genes- *hypo*.*encap* (25 kDa and 31.4 kDa)

### Cloning into the plasmid pHT01

PCR-amplified sequences were cloned into plasmid pHT01 (MoBiTec GmbH, Göttingen, Germany) (Figure S2) using an Infusion HD cloning kit (TakaRa Bio, San Jose, CA, USA). Briefly, plasmid pHT01 was digested with *Bam*HI and *Xma*I (The New England BioLabs, Ipswich, MA, USA) then gel purified as above. Purified linear plasmid and respective PCR products were used in the infusion cloning reaction, according to the manufacturer’s instructions. Cloning reactions were then used to transform *Escherichia coli* stellar cells (Clontech Laboratories, TakaRa Bio, San Jose, CA, USA) according to manufacturer’s instructions with selection on LB agar containing 100 μg/mL carbenicillin. Plasmid DNA was extracted using a NucleoSpin Plasmid Mini kit (Macherey-Nagel, USA). Plasmids were sequenced by Macrogen (Seoul, Korea), and sequencing data were aligned to those of the respective constructs using Geneious Prime-2020 (Biomatters, New Zealand) and then manually curated. Plasmid DNA from one correct construct was used to transform *Bs* WB800N. The Gram-positive bacterium, *Bs* WB800N (*nprE aprE epr bpr mpr::ble nprB::bsr Δvpr wprA::hyg cm::neo*; NeoR) was used for transformation (Jeong et al. 2018; Wu et al. 2002). Electrocompetent cells of *Bs* WB800N were prepared following the protocol of Lu et al. (2012). Validated *Bs* WB800N transformants of pHT01-*hypo* (A1, A2), pHT01-*encap* (B1, B2), and pHT01-*hypo*.*encap* were independently cultivated overnight in LB broth until the culture attained OD_600nm_ 0.7–0.8 and aliquoted into two parts. An aliquot (7.5 mL) was induced with 1 mM IPTG (Isopropyl β-D-1-thiogalactopyranoside), and the other was uninduced. Both induced and uninduced samples were harvested after 3.5 and 24 h of treatment. The resultant cultures were centrifuged at 6000 × g for 10 min at 4 °C to spin down the cells. Cell pellets were stored at −20 °C, and the supernatant filter was sterilised by passing twice through a 0.22 *μ*m filter.

### Assay test of expressed proteins

CFS of *Bs* WB800N containing the (uninduced and induced) proteins corresponding to the pHT01-*hypo* (25 kDa), pHT01-*encap* (31.4 kDa), and pHT01- *hypo*.*encap* encoding both the proteins was evaluated using the Kirby-Bauer disc diffusion test (Kirby et al. 1956) against *Bl* 1821L and *Bl* 1951 as the host bacteria. CFS of *Bs* WB800N without transformation (pHT01-*hypo*, pHT01-*encap*, pHT01-*hypo.encap*) was tested for bioactivity, and none was detected.

### Purification and SDS-PAGE analysis of expressed proteins

CFS of expressed proteins (pHT01-*hypo*, pHT01-*encap*, pHT01-*hypo.encap*) in the Gram-positive bacterium *Bs* WB800N and demonstrating antagonistic activities were concentrated and purified with some differences using the sucrose density gradient centrifugation as outlined above. Sucrose density gradients of 10%, 20%, 30%, 40%, 50%, and 60% were sequentially applied in layers of 1.25 mL of freshly prepared sucrose solution. Purified and expressed protein (pHT01-*hypo*, pHT01-*encap*, pHT01-*hypo.encap*) CFS were again concentrated before running on gel electrophoresis. SDS-PAGE of sucrose density gradient purified *Bs* WB800N CFS without transformation (pHT01-*hypo*, pHT01-*encap*, pHT01-*hypo.encap*) was also developed for comparative analysis.

## Results

### Bioactivity assay, electron microscopy, and SDS-PAGE analysis of putative ABPs

**Fig. 1:**
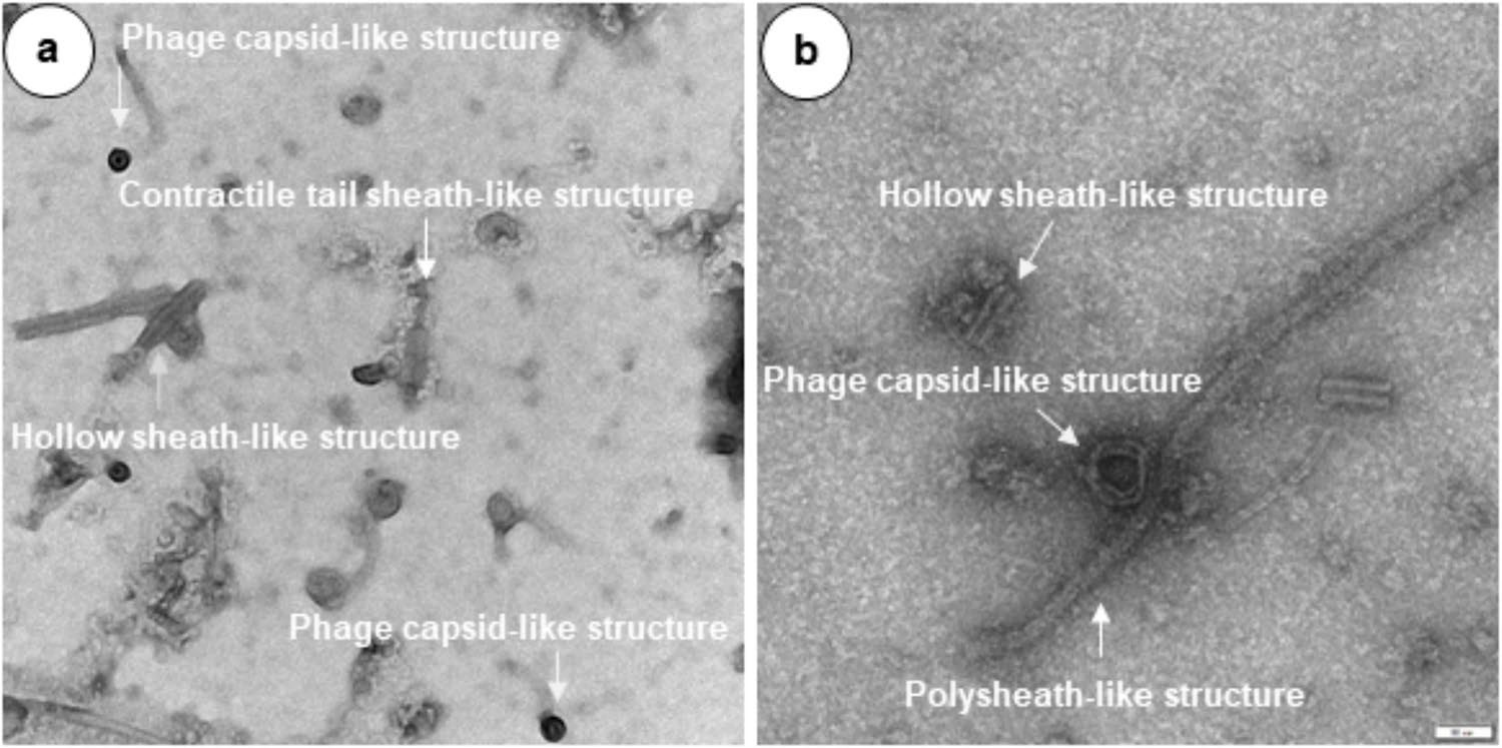
Electron micrographs of crude lysates of *Bl* 1821L (**a**) and *Bl* 1951 (**b**) showing globular or phage capsid-like, hollow sheath-like, contractile phage tail sheath-like, and polysheath-like structures

**Fig. 2:**
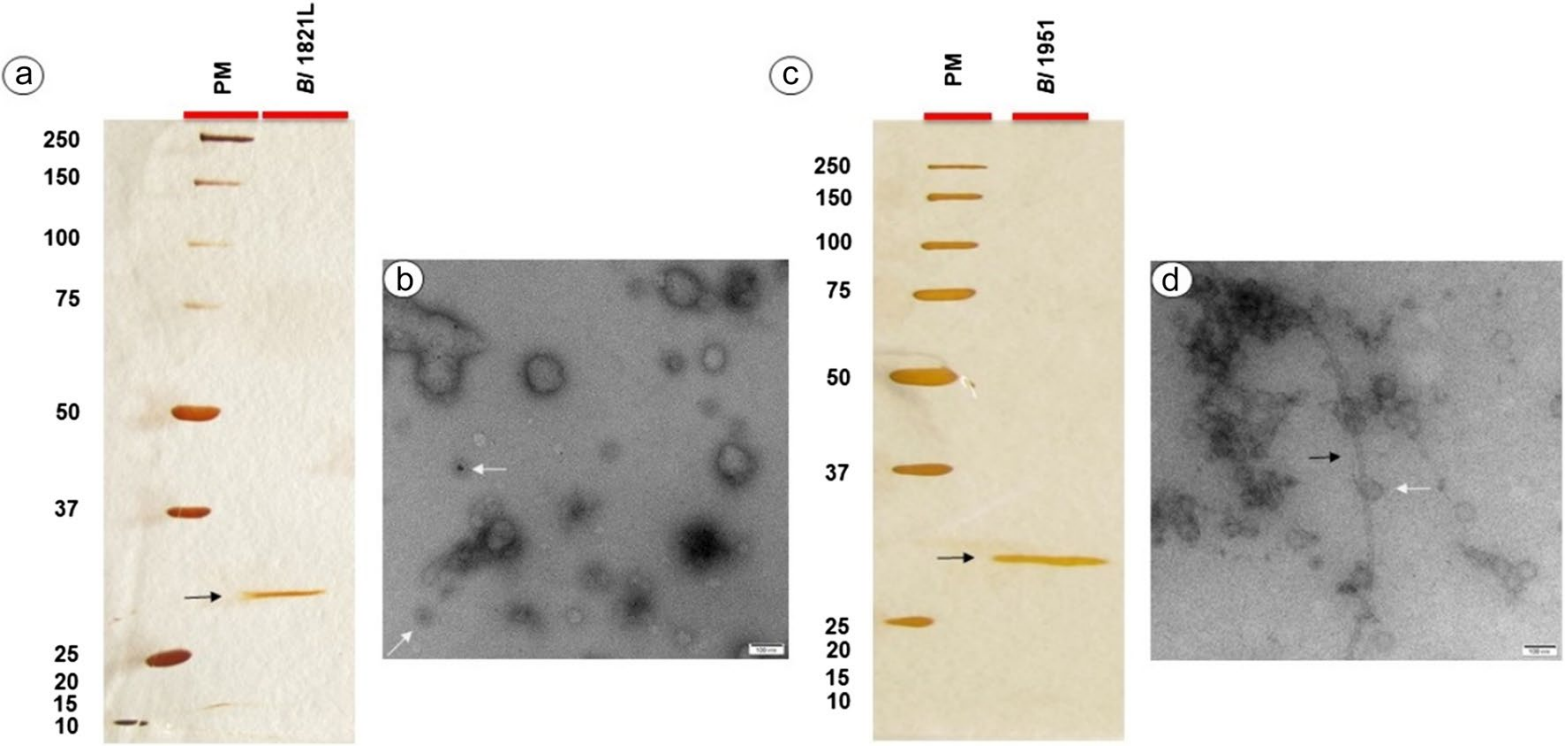
SDS-PAGE showing the *Bl* 1821L and *Bl* 1951 sucrose density gradient centrifugation purified and 10 kDa MWCO membrane concentrated ~30 kDa putative antibacterial protein (**a**, **c** shown with dark blue arrow). Arrows in electron micrographs of purified putative antibacterial protein of *Bl* 1821L denote globular or phage capsid-like structures (**b** shown with white arrow) while for *Bl* 1951, globular or phage capsid-like structures (**d** shown with white arrow) and polysheath-like structures (**d** shown with dark blue arrow) are visualised. Scale bar= 100 nm. PM denotes protein marker

Mitomycin C-induced CFS of *Bl* 1821L and *Bl* 1951in the bioactivity assay against the producer and the vice versa strain produced a prominent zone of inhibition, and no such activity was noted in the negative control (Figure S3). TEM analysis of the crude lysates of *Bl* 1821L (Fig. 1a) and *Bl* 1951 (Fig. 1b) showed similar structures including globular or capsid, hollow sheath, contractile phage tail sheath, and polysheath-like structures. Purified lysates of *Bl* 1821L and *Bl* 1951 after 10 kDa molecular weight cutoff (MWCO) membrane concentration yielded a single protein band of ~30 kDa (Fig. 2a, c). Assessments of the concentrated protein of *Bl* 1821L under electron microscope predominantly displayed the presence of globular or hexagonal structures that resembled phage capsids (Fig. 2b) while for *Bl* 1951 globular or phage capsid-like structures and long nanotubes similar to polysheaths were seen (Fig. 2d).

### Genome sequence analysis of *Bl* 1821L and *Bl* 1951

Analysis of the *Bl* 1821L (GenBank accession NZ_CP033464.1) and *Bl* 1951 (GenBank accession RHPK01000003.1, contig 1) genomes using antiSMASH identified several gene clusters responsible for the production of potential secondary metabolites including petrobactin, basiliskamide A, bogorol A, zwittermicin, and laterocidine (Figure S4, S5). Using the programme MIBiG 3.0, these predicted gene clusters were aligned with the known similar gene clusters of Gram-positive bacteria *Bacillus anthracis* strain Ames (GenBank accession AE016879.1), *Bl* PE36 (GenBank accession AXBT01000013), *Bl* DMS 25 (GenBank accession KY810814), *Bacillus cereus* strain UW85 (GenBank accession FJ430564.1), and *Bl* LMG 15441 (GenBank accession CP007806) respectively, and the percentage similarity of these gene clusters is shown in Figure S4, S5. BAGEL4 analysis of the *Bl*1821L and *Bl* 1951 genomes identified different areas of interest (AOI) that had the potential to produce ribosomally synthesised antimicrobial peptides (laterosporulin, sactipeptides, UviB, lanthipeptide, linear azole-containing peptides (LAPs)) (Table S1). Notably, among the predicted AOI, two areas matched to the already defined core peptides (bacteriocins), laterosporulin (Singh et al. 2012) of *Bl* GI-9 (GenBank accession HE579167.1), and UviB (Anderson et al. 2005) of *Bacillus thuringiensis* (Bt) *serovar israelensis* ATC35646 (GenBank accession AAJM01000279) (Table S1, Figure S6, S7). Results of the percentage amino acid identity of the antiSMASH- and BAGEL4-predicted antimicrobial molecules are presented in Table S1. However, to authenticate the nature of putative ABPs, purified protein bands of ~30 kDa were excised for N-terminal sequence analyses.

### Identification of ~30 kDa putative antibacterial protein

**Fig. 3:**
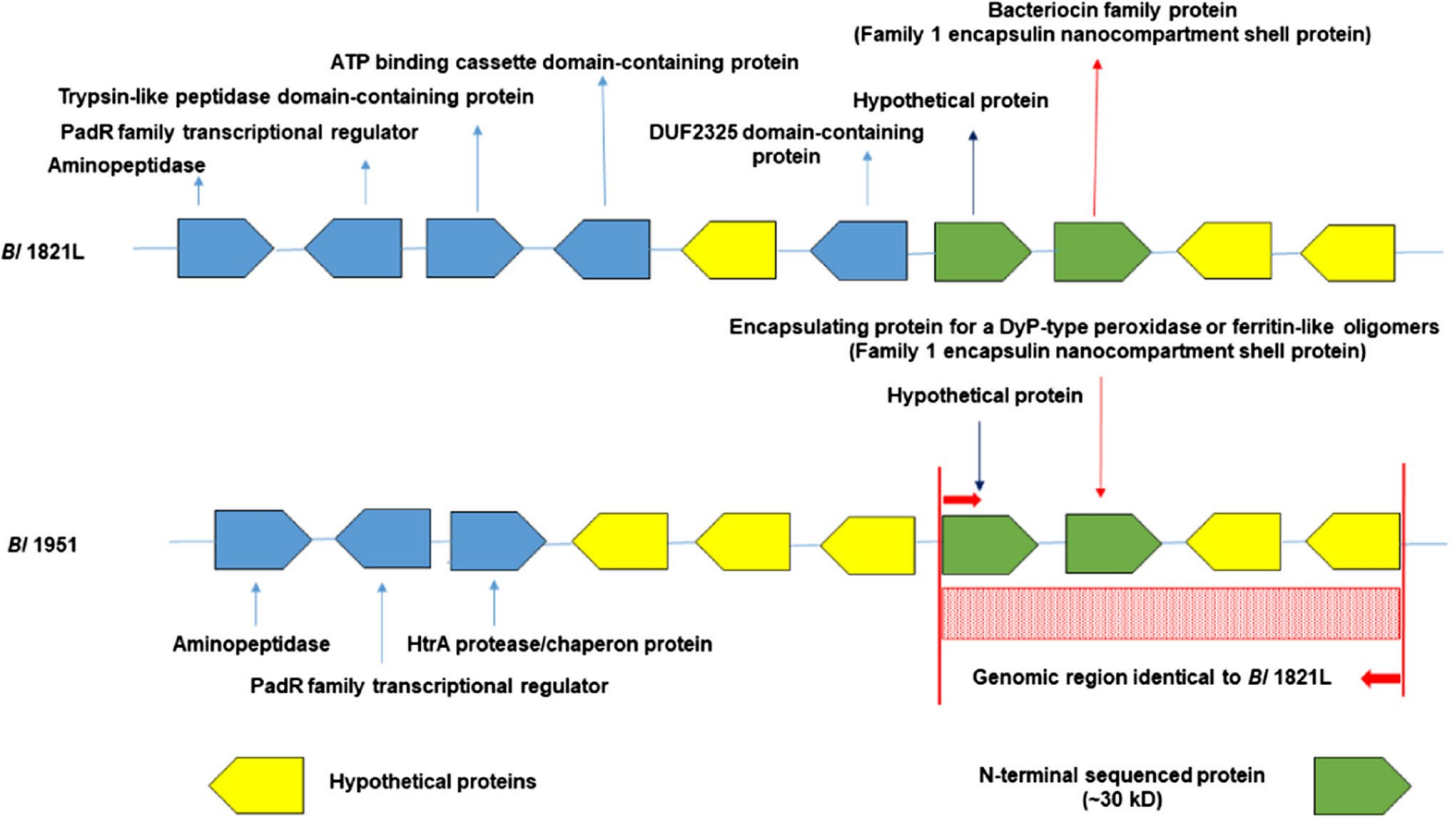
*Bl* 1821L and *Bl* 1951 genomic architecture of ~30 kDa N-terminal sequence showing an identified hypothetical protein of 25 kDa (dark blue arrow) and a bacteriocin family protein (encapsulating) of 31.4 kDa (red arrow) residing in *Bl* 1821L and *Bl* 1951 genomes along with other proteins of the region with terminology used in GenBank accessions. *Bl* 1951 genomic region identical to *Bl* 1821L genome is highlighted with red shaded box region

The amino acid sequences derived from N-terminal sequencing of the purified ~30 kDa protein band were used to predict the corresponding gene in the *Bl* 1821L genome (GenBank accession NZ_CP033464.1). Analysis identified several matches to a hypothetical protein (A0A502I846, covering 57% of the amino acid sequence; Figure S8) and a bacteriocin family protein (A0A502IA18, covering 78% of the amino acid sequence; Figure S9). The genes encoding the hypothetical protein (GenBank accession WP_113757161) and the putative bacteriocin family protein (GenBank accession WP_113757162) residing adjacently were identified (Fig. 3, Figure S10). Amino acid sequence analysis through ExPasy computed the molecular weight of the identified hypothetical and bacteriocin family proteins of *Bl* 1821L to be 25 kDa and 31.4 kDa. The identification of two proteins suggested co-migration of proteins. The molecular mass of the predicted 31.4 kDa protein was within the expected range estimated by SDS-PAGE analysis, i.e., ~30 kDa (Fig. 2a). The same genes and predicted proteins were also identified in the *Bl* 1951 genome (GenBank accession RHPK01000003.1, contig 1). For *Bl* 1951, the genes encoding these proteins were annotated as putative encapsulating protein for a DyP-type peroxidase or ferritin-like protein (31.4 kDa) and hypothetical protein (25 kDa) in the GenBank annotated deposits (Fig. 3, Figure S11).

### BLASTp analysis of the identified putative ABPs

BLASTp analysis of the identified putative antibacterial protein (31.4 kDa) of *Bl* 1821L and *Bl* 1951 (GenBank accession WP_113757162.1) against the GenBank database shared 98.6 % amino acid identity to the bacteriocin family protein of *Brevibacterium* sp. JNUCC-42 (GenBank accession QOS99091.1) and >95% amino acid identity to the family 1 nanocompartment shell protein of the different species of the *Brevibacillus* (Table S2). The 25 kDa identified hypothetical protein (GenBank accession WP_113757161.1) displayed 98.6%, 91.2%, and >90% amino acid identity to the *Brevibacterium* sp. JNUCC-42 (GenBank accession QOS99092.1), *Brevibacillus* sp. SKDU10 (GenBank accession WP_064017301.1), and several bacteria belonging to the genus *Brevibacillus* (Table S3).

### Bioinformatic analyses of ~30 kDa putative antibacterial protein

The identified gene (GenBank accession WP_113757162.1) encoding a putative bacteriocin family protein in the *Bl* 1821L at the 5′ end was flanked by two genes encoding hypothetical proteins (GenBank accessions WP_113757163 and WP_119732342 ) while at the 3′ end, another identified gene encoding the 25 kDa hypothetical protein resided (Fig. 3, Figure S10). Notably, this genomic region presented similar organizational architecture to *Bl* 1951 genome (GenBank accession RHPK01000003.1, contig 1) (Fig. 3, Figure S11). Different families of transcriptional regulator proteins including PadR, MarR, Helix-turn-Helix (HEH), and other domain containing proteins such as the trypsin-like peptidase and bacillithiol system redox-active protein (YtxJ) were present (Figure S10). Furthermore, the gene encoding the stress protein (YtxJ) in *Bl* 1821L (Figure S10) and its ortholog in *Bl* 1951 was noted but annotated as Pyridoxamine 5′-phosphate oxidase (EC 1.4.3.5) CDS (CoDing sequence) in *Bl* 1951 (Figure S11).

### Bactericidal activity of putative encapsulating protein

AMPA analysis of the 31.4 kDa putative EP sequence of *Bl* 1821L revealed an index value below the threshold level, which suggested its bactericidal potency (Figure S12). The region of amino acid similarity covered amino acids −2 to 279 with a propensity value of 0.001 (0%) and a mean value of 0.001 (Figure S12). CPP specific amino acids and motifs identified in the *Bl* 1821L (identical to the *Bl* 1951) EP (31.4 kDa) sequence are presented in Table S4 and Figure S13. The identified genes and amino acid content of N-terminal sequenced and bioinformatically analysed ~30 kDa protein band of *Bl* 1951 were 100% identical to the identified 31.4 kDa *Bl* 1821L putative EP. Therefore, the resultant bactericidal activity predicted through bioinformatic tools AMPA and CellPPD is likely to be similar.

**Fig. 4:**
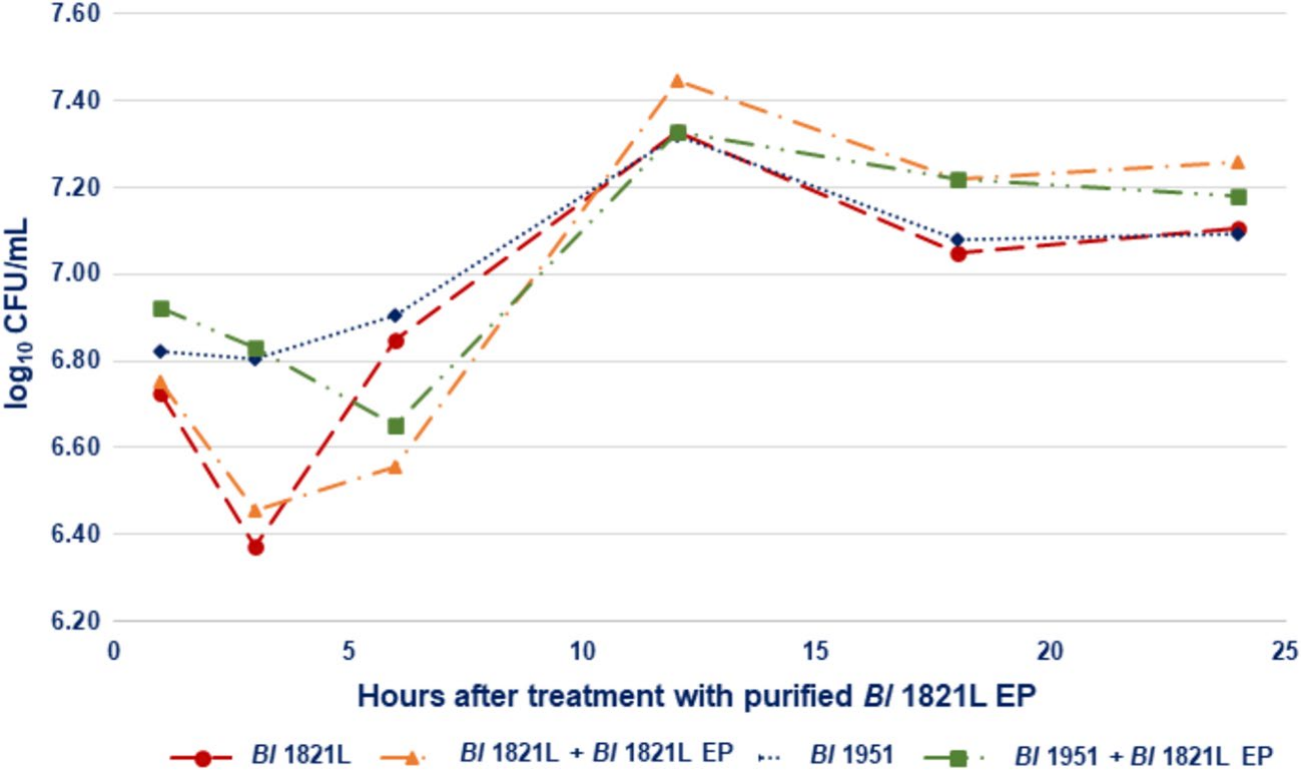
Number of viable cells (log_10_ CFU/mL) of *Bl* 1821L and *Bl* 1951 with/without treatment of purified *Bl* 1821L putative encapsulating protein (~30 kDa) after incubation at 30 °C for various time intervals

The crude lysate of *Bl* 1951 harbouring the putative ABPs in the liquid assay exhibited the autocidal activity by showing a non-significant decrease of 48.4% in the number of viable cells after 6 h of incubation at 30 °C (Table S5, Figure S14). However, for the same time intervals, the addition of ~30 kDa-purified putative EP of *Bl* 1821L and *Bl* 1951, antibacterial activities against both the isolates, were noted (Table S7, S9, Fig. 4, Figure S17). Spectrophotometric readings (OD_600nm_) of ~30 kDa-purified putative EP-treated and untreated cultures similar to the crude lysates revealed very small changes across the evaluated time intervals (Table S6, S8, S10, Figure S15, S16, S18).

### LIVE/DEAD staining

**Fig. 5:**
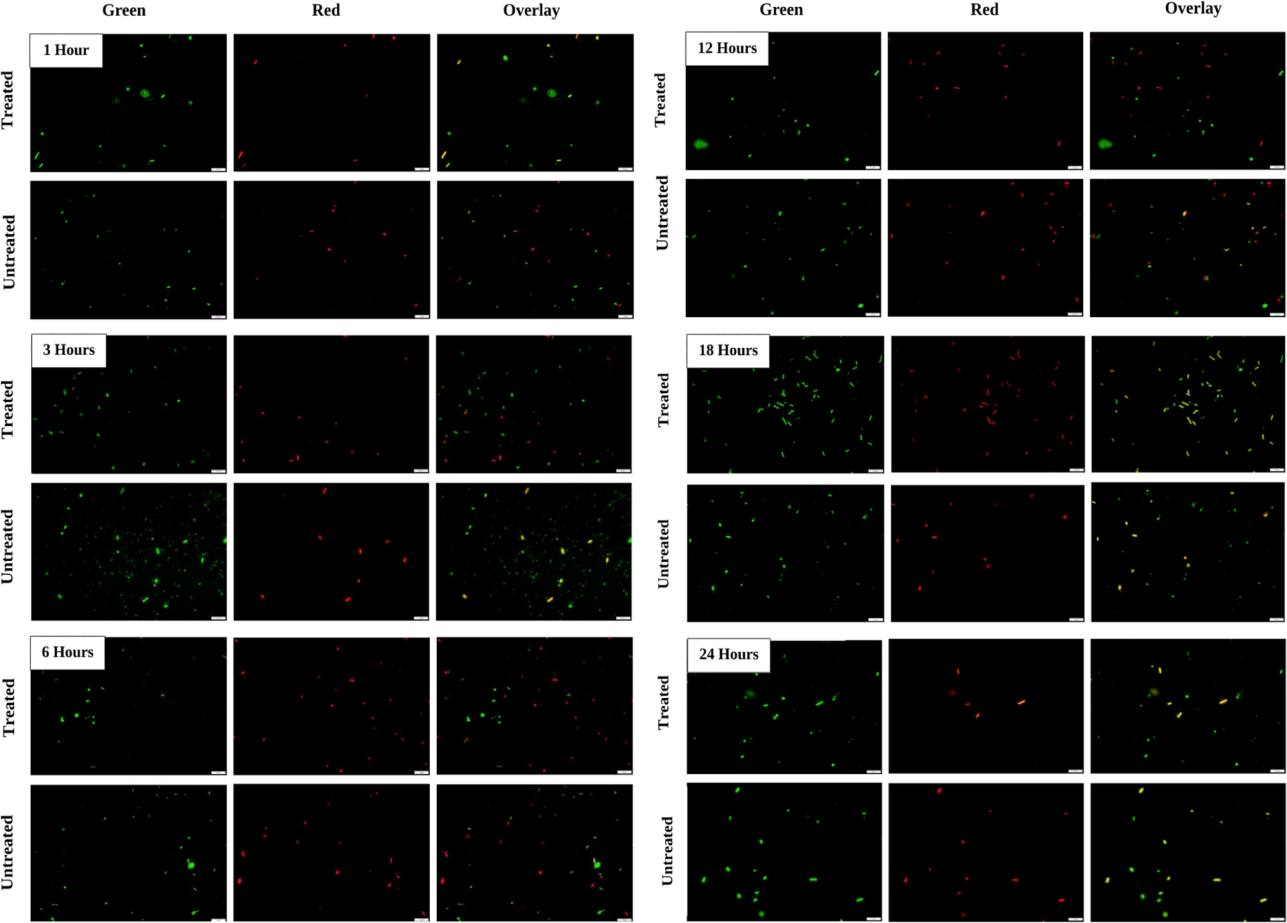
LIVE/DEAD staining of *Bl* 1821L cells after treatment with the purified *Bl* 1821L putative encapsulating protein (~30 kDa). Green denotes live cells while red and orange show the cells with compromised membranes. Scale = 10 μm

**Fig. 6:**
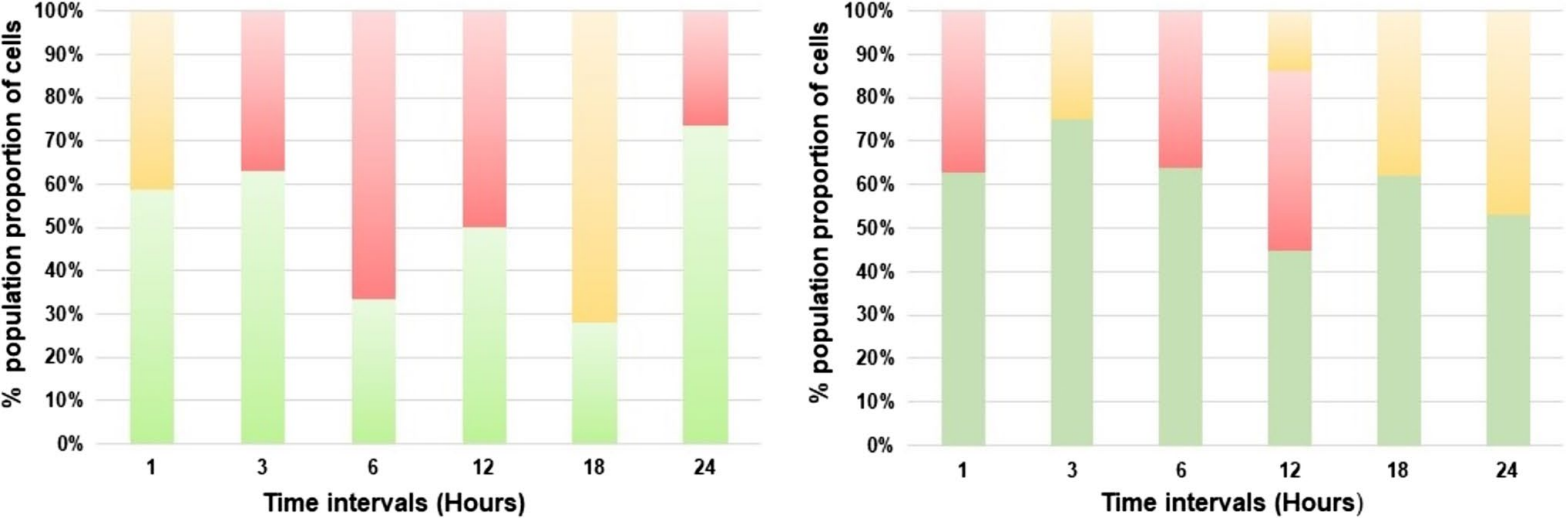
LIVE/DEAD stained percentage population proportion of *Bl* 1821L cells after treatment (left side graph) with the ~30 kDa-purified putative encapsulating protein of *Bl* 1821L and without treatment (right side graph). Green denotes live cells, and red and orange show the proportion of cells with compromised cell membranes

LIVE/DEAD staining of *Bl* 1821L-treated culture with the ~30 kDa-purified putative EP of *Bl* 1821L did not show a higher population of red cells (cells with compromised membranes) after 1 h of incubation than in the control treatment (without EP) (Fig. 5). After 3 h, more red cells were seen with the ~30 kDa putative EP-treated culture as compared to the control where yellow/orange colour cells were predominant (Fig. 5). *Bl* 1821L cells with compromised cell membranes (red) were prominent after 6 and 12 h of incubation with the purified putative EP, which was similar to the control (Fig. 5). Notably, the appearance of red cells coincided with the decrease in the number of viable cells (48.9%) 6 h after treatment with the purified putative EP but in contrast to the 12-h treatment (Table S7, Figs. 5 and 6). On a percentage basis, 6 h after treatment, 58.8% of the putative EP-treated and 37.5% of the untreated cells were red (Fig. 6). LIVE/DEAD staining of *Bl* 1821L-treated culture with the ~30 kDa-purified putative EP of *Bl* 1821L at a higher volume (200 μL) resulted in more cells with compromised membranes (red) from the initial 1 h of incubation up to 6 h when compared to the control (without EP). However, yellow/orange cells in the *Bl* 1821L culture with the purified ~30 kDa EP were dominant from 12 to 24 h of incubation at 30 °C (Figure S19).

### Expression of *B. laterosporus * 25 kDa and 31.4 kDa proteins in *B. subtilis* WB800N

The CFS of the expressed proteins pHT01-*hypo* (25 kDa), pHT01-*encap* (31.4 kDa), and the both (pHT01-*hyo*.*encap*) were used for further analysis. *Bs* WB800N supernatant (pHT01-*hypo*, A1) expressing the 25 kDa hypothetical protein demonstrated antagonistic activity against *Bl* 1951 by developing a zone of inhibition of 11.7 mm and 12 mm with induced and uninduced cultures respectively after 3.5 h of induction (Table S11, Figure S20b). For *Bl* 1821L, the same supernatant demonstrated no significant activity (Table S12, Figure S20a). Similarly, 25 kDa hypothetical protein (pHT01-*hypo*, A2) after 3.5 h of induction exhibited similar activity against both strains (*Bl* 1821L and *Bl* 1951) by developing a zone of inhibition of 11.7 mm and 11 mm respectively. However, after 24 h of induction, antagonistic activity was almost absent (Table S11, S12, Figure S21a, S21b). Purification of the corresponding protein was only visualised on SDS-PAGE with the 25 kDa hypothetical protein sample (pHT01-*hypo*, A1) (Figure S20c), and no protein bands were seen with the other concentrated protein (pHT01-*hypo*, A2). Unexpectedly, a defined band of ~48 kDa in the 40% gradient, faint bands of the same level in other gradients, and prominent protein bands of >48 kDa in the 20% gradient were visualised on SDS-PAGE with the purified and concentrated 25 kDa hypothetical protein (pHT01-*hypo*, A1) (Figure S20c). Assay test of the CFS of *Bs* WB800N without any transformation (pHT01-*hypo*, pHT01-*encap*, pHT01-*hypo.encap*) showed no zones of inhibition against *Bl* 1821L and *Bl* 1951 (Table S11, S12, Figure S22). In addition, no bands were observed on silver-stained SDS-PAGE of these concentrated supernatants (Figure S22).

**Fig. 7:**
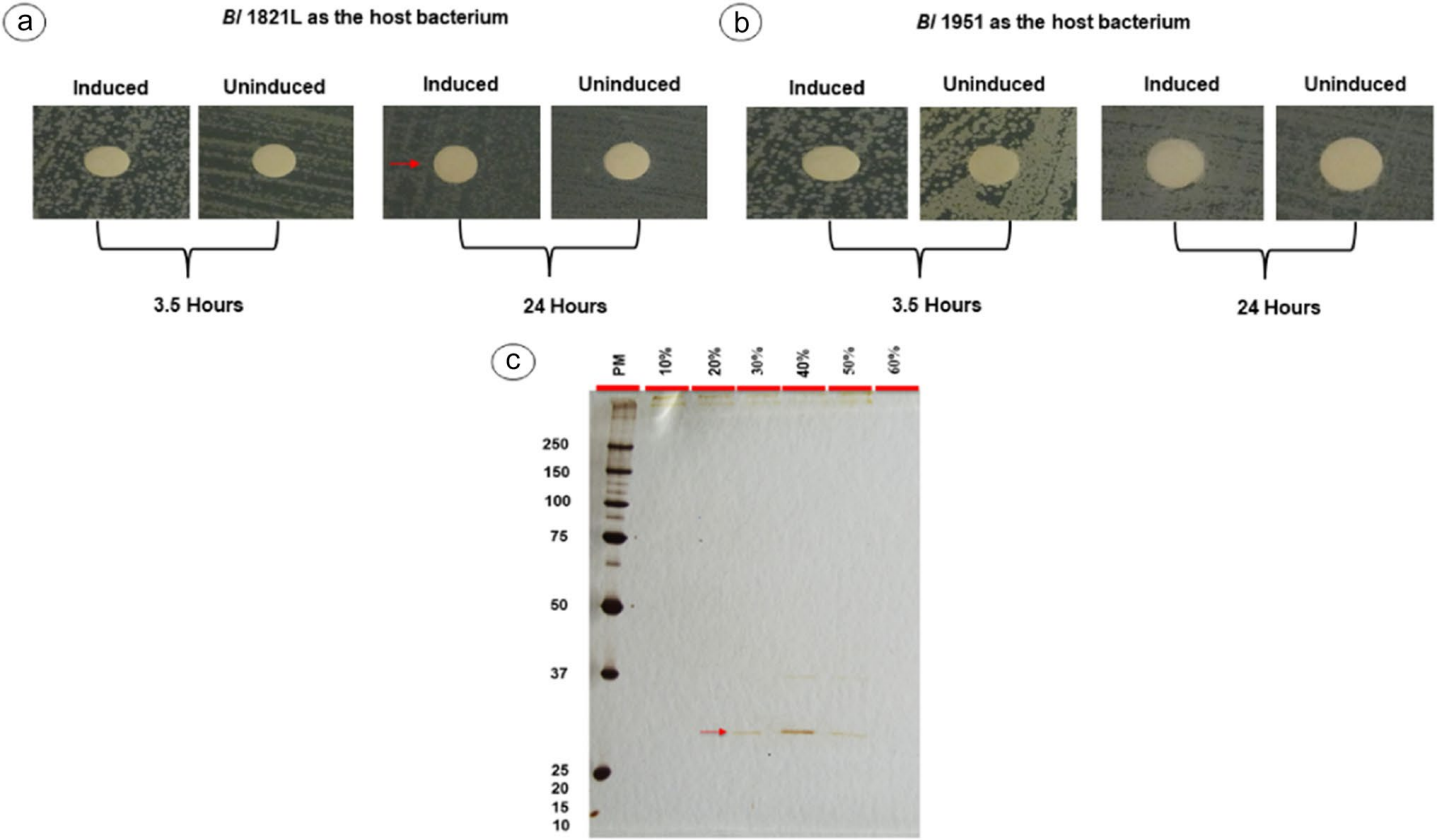
Assay test of CFS from *Bs* WB800N (pHT01-*encap*, B2) expressing 31.4 kDa putative encapsulating protein against *Bl* 1821L (**a**) and *Bl* 1951 (**b**) as the host bacterium. Arrows (red) denote the zones of inhibition showing a diameter of ≥11 mm. SDS-PAGE analysis showing a purified ~30 kDa putative encapsulating protein (**c** shown with red arrow) expressed after 24 h of induction from *Bs* WB800N (pHT01-*encap*, B2). PM denotes protein marker

Antibacterial activity was found in CFS (pHT01-*encap*, B1) containing 31.4 kDa putative EP against *Bl* 1821L as the host bacterium, developing a lysis zone of 12.3 mm after 24 h of induction and 11.7 mm without induction (Table S12, Figure S23a). For *Bl* 1951, a halo zone of 11 mm was noted 24 h after induction (Table S11, Figure S23b). SDS-PAGE analysis of the 31.4 kDa-concentrated putative EP (pHT01-*encap*, B1) after 3.5 h of induction displayed a very faint band of ~30 kDa (Figure S23c), but after 24 hours of induction, in addition to the ~30 kDa band, other proteins were also present (Figure S23d). Putative 31.4 kDa EP (pHT01-*encap*, B2) exhibited inhibitory activity solely against *Bl* 1821L as the host bacterium by producing a zone of inhibition of 11 mm after 24 h of incubation (Table S12, Fig. 7a), and a purified band of ~30 kDa in 40% gradient was visualised on SDS-PAGE (Fig. 7c).

**Fig. 8:**
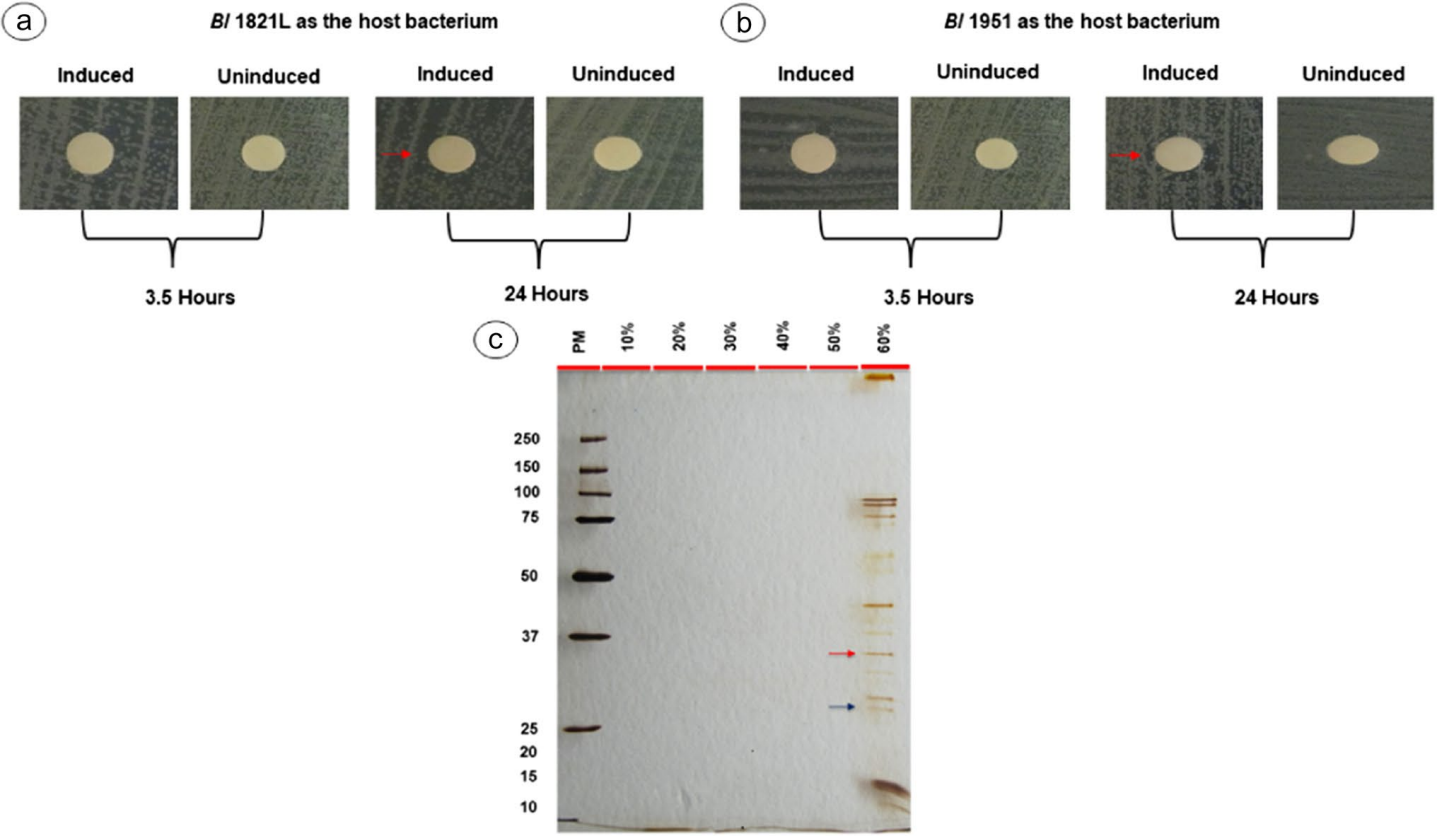
Assay test of CFS from *Bs* WB800N (pHT01-*hypo.encap*) expressing both 25 kDa hypothetical and 31.4 kDa putative encapsulating proteins against *Bl* 1821L (**a**) and *Bl* 1951 (**b**) as the host bacterium. Arrows (red) denote the zones of inhibition showing a diameter of ≥11 mm. SDS-PAGE analysis showing the purified ~25 kDa hypothetical (shown with dark blue arrow) and ~30 kDa encapsulating proteins (**c** shown with red arrow) from *Bs* WB800N (pHT01-*hyo.encap*). PM denotes protein marker

*Bs* WB800N supernatant expressing (pHT01-*hyo*.*encap*) with both the hypothetical (25 kDa) and putative encapsulating (31.4 kDa) proteins displayed prominent antibacterial activity against *Bl* 1821L and *Bl* 1951 after 24 h of induction by causing inhibition zones of 11 mm and 12 mm respectively (Table S11, S12, Fig. 8a, b). SDS-PAGE analysis of purified and concentrated culture (pHT01-*hyo*.*encap*) displayed numerous prominent bands including a ~30 kDa protein in the 60% gradient (Fig. 8c).

## Discussion

In this study, we determined the identity of an antibacterial protein produced by two insect pathogenic isolates *Bl* 1821L and *Bl* 1951. Initially, the genome sequence analysis of the *Bl* 1821L and *Bl* 1951through antiSMASH and BAGEL4 revealed the presence of gene clusters associated with the production of non-ribosomally synthesised peptides (petrobactin, bogorol A, basiliskamide A, zwittermicin A, and laterocidine) and ribosomally synthesised peptides (laterosporulin, sactipeptides, UviB, and LAPs). Importantly, among the latter, group matches were found to the previously defined low molecular-weight (LMW) bacteriocins such as laterosporulin (5.75 kDa) of *Bl* GI-9 (Singh et al. 2012) and UviB (8.78 kDa) of *Bt *serovar* israelensis* (Anderson et al. 2005). However, subsequent experimental work involving the isolation and sucrose density gradient purification yielded a homogeneous protein of ~30 kDa on SDS-PAGE. N-terminal sequencing of the ~30 kDa-purified putative antibacterial protein bands of *Bl* 1821L and *Bl* 1951 located the putative genes encoding the HMW proteins (bacteriocins) in their genomes. A gene encoding the 25 kD hypothetical protein at the 3′ end of the identified putative EP (31.4 kDa) encoding gene was also found in both the strains. Bioinformatic analyses of the 31.4 kDa EP sequence using the tools AMPA and CellPPD identified the potential bactericidal motifs. The killing potency of the putative ABPs present in the crude and purified lysates of *Bl* 1821L and *Bl* 1951 against the cells of both the strains was determined growing in broth and corroborated with the LIVE and DEAD staining. Furthermore, the genes encoding the 25 kDa hypothetical protein, 31.4 kDa putative EP, and both the genes (25 kDa and 31.4 kDa) of *Bl* 1821L were transformed in a Gram-positive bacterium *Bs* WB800N for heterologous expression, with activity confirmed for expression of both proteins (Jeong et al. 2018).

Genes encoding the identified putative antibacterial protein of *Bl* 1821L and *Bl* 1951 shared >97% amino acid identity to the family 1 encapsulin nanocompartment shell protein belonging to the multispecies of the genus *Brevibacillus*. Nanocompartment proteins are generally divided into four families, and the members in the family 1 are called “classical encapsulins”. This distinctive protein is commonly found in eubacteria and archaea (Al-Sammak 2014; Valdes-Stauber and Scherer 1996), and so far, 31 out of 35 prokaryotic phyla are known to encode encapsulin-like operons (Giessen and Silver 2017). In addition, the nanocompartments also contain ferritin-like compounds or peroxidase enzymes (McHugh et al. 2014). Ferritin family proteins include three sub-families: classical ferritin (Ftn), bacterioferritin (Bfr), and the DNA-binding protein from starved cells (Dps) (Andrews 2010; Theil et al. 2013). The first two categories (Ftn and Bfr) perform diverse functions including ribonucleotide reductase (Åberg et al. 1993), protecting DNA from oxidative damage (Grant et al. 1998), and iron storage (Bradley et al. 2014). However, Dps proteins are unique on account of their role in detoxification of iron as opposed to its storage (Karas et al. 2015; Orban and Finkel 2022), and a failure to detoxify iron may prove fatal to the cells (Bradley et al. 2020; He et al. 2016). *Mycobacterium tuberculosis*, a pathogenic bacterium, accommodates two putative iron storage proteins, BfrA (Rv1876) a bacterioferritin and BfrB (Rv3841) a ferritin-like protein. The expression of both *bfrA* and *bfrB* is regulated by the binding of iron-activated *ideR* (iron-dependent regulator) to the tandem operator sites present at the 5′ end of the iron storage gene (Cole et al. 1998). Reddy et al. (2012) generated mutants lacking these functional genes. Interestingly, the authors found that the mutant bacterium under iron-deficient conditions not only showed a significant decrease in growth but was also vulnerable to oxidative stress (Reddy et al. 2012). In this context, it is possible that the 31.4 kDa putative EP of *Bl* 1821L and *Bl* 1951 might be undergoing iron deficiency, or the ferritin proteins failed to detoxify the excessive iron which contributed towards the stunted growth of both the strains.

Encapsulin, originating from the supernatants of *Br. linens* M18, was initially designated “bacteriocins” due to their inhibitory effect on the growth of several Gram-positive bacteria, but no lethal effect was noticed against the Gram-negative bacteria (Eppert et al. 1997; Valdes-Stauber and Scherer 1996). The newly found 31 kDa biomolecule (Linocin M18) lost its antagonistic activity upon purification (Valdés-Stauber and Scherer 1994). In our study, the sucrose density gradients containing the purified ~30 kDa putative EP (Linocin M18) of *Bl* 1951 and *Bl* 1821L sustained bioactivity in the paper disc assay. Furthermore, the ~30 kDa-purified putative EP of *Bl* 1951 and *Bl* 1821L in the liquid assay exhibited an autolytic activity which was evident from the decrease in the number of viable cells of *Bl* 1951 and *Bl* 1821L after 3 and 6 h of treatment. Typically, the putative ABPs (bacteriocins) inhibit growth and kill a broad array of microbial species or just closely related strains of the same species, and the producer strains are immune to their toxic effects (Ghequire and De Mot 2015; Riley and Wertz 2002). However, some members of a genetically identical population can kill their siblings (autocidal) (Popp and Mascher 2019) like bacteriocin hyicin 3682 (Fagundes et al. 2011). Miljkovic et al. (2019), while assessing the antimicrobial potential of different *Bl* strains (BGSP7, BGSP9, BGSP11), also found their pesticidal potential against the potato beetle, *Leptinotarsa decemlineata*, and some crop pathogenic fungi. The authors in their preliminary work noticed the autocidal (self-killing) activity of *Bl* BGSP7, *Bl* BGSP9, and *Bl* BGSP11 strains which they cited due to the synthesis of numerous antimicrobial molecules. In addition, antiSMASH analysis of the three potential insect pathogenic isolates predicted a 31.4 kDa family 1 encapsulating nanocompartment shell protein (Linocin M18), but the following research revealed that among the several purified antagonistic compounds, one antimicrobial molecule of 1.5 kDa produced by the *Bl* BGSP7 and *Bl* BGSP9 and two antimicrobial molecules of 1.5 kDa and 6 kDa produced by *Bl* BGSP11 were found to be more potent than others. Though, the BLASTp analysis of the 31.4 kDa-identified putative EP protein (Linocin 18) in our study exhibited 97.1%, 97.1%, and 96.4% amino acid identity with the similar protein of *Bl* BGSP7, *Bl* BGSP9, and *Bl* BGSP11 respectively. The Gram-positive bacterium *Bacillus subtilis* (*Bs*) exhibits the self-killing activity under nutritional stress environment (Popp and Mascher 2019). The *Bs* cells at the onset of sporulation secrete extracellular killing factors and lyse the sibling non-sporulating cells that have not developed immunity to these toxins. In addition, the resultant lysis also releases nutrients from the dead cells into the starved medium that the surviving sporulating cells can feed on, this behaviour was called “cannibalism” (González-Pastor 2011; Lamsa et al. 2012). The cannibalistic phenomenon has some biological significance for the bacterial populations as the *Bs* population could be at risk if most of the members engage in sporulation at once (Höfler et al. 2016).

Electron micrographs of the crude lysates of both the *Bl* 1821L and *Bl* 1951 showed similar structures including the globular or phage capsid-like (encapsulating) and contractile phage tail-like structures. Typically, family 1 encapsulin is assembled from a single type of shell protein into compartments of 24 to 42 nm diameter with either T1, T3, or T4 icosahedral symmetry (Akita et al. 2007; Sutter et al. 2008). Therefore, the appearance of the ~30 kDa-purified protein suggests that ~30 kDa assembles as an icosahedral (capsid-like) particle. Recently, we isolated, purified, and characterised the putative phage tail-like protein (bacteriocin) of ~48 kDa from the insect pathogenic isolate *Bl* 1821L (Babar et al. 2022a). Based on these findings, it can be summarised that the crude lysates of *Bl* 1821L and *Bl* 1951 harbour the ~30 kDa and ~48 kDa putative ABPs. Notably, these putative ABPs of both the isolates demonstrated autocidal activity, but the purified ~30 kDa putative EP showed antagonistic activity against *Bl* 1821L and *Bl* 1951. Variation in the antagonistic activity of crude lysate (~30 kDa and ~48 kDa) and the purified putative EP (~30 kDa) might be attributed to the prevalence of a toxin-antitoxin mechanism (Hayes and Kędzierska 2014). In this stance, it is likely that in the crude state, either the ~30 kDa or ~48 kDa putative ABP is masking the toxic effect of the other.

Encapsulin proteins seize a fold of capsid protein of lambdoid bacteriophage in their shell protein (formerly referred to as linocin-like protein) (Gabashvili et al. 2020; Suhanovsky and Teschke 2015); therefore, it is likely that encapsulins and the capsid proteins of tailed phages have evolved from a common ancestor (Duda and Teschke 2019; Orlova et al. 2012). However, viral capsids and encapsulin nanocompartments functionally differ; the former transport viral genomes from one cell to another and the latter are involved in metabolic activities (Giessen 2022; Nichols et al. 2017). A search of the identified 31.4 kDa family 1 encapsulin nanocompartment shell protein (Linocin M18) of *Bl* 1821L and *Bl* 1951 in the Pfam (protein families) database revealed its association with the clan (CL0373) of phage coat superfamily proteins (Mistry et al. 2021). Virus structural proteins (capsid) often encode various bioactive peptides such as antimicrobial peptides (AMPs) and CPPs (Freire et al. 2015; Järver and Langel 2006). Bioinformatic tool AMPA revealed the bactericidal potential of the 31.4 kDa putative EP, and CellPPD identified the motifs in the bactericidal stretch that have the faculty to penetrate the cells. Typically, CPPs are short cationic peptides, usually 5–30 amino acid residues, which can be found in a wide range of natural sources from microbes to plants and animals (Milletti 2012; Zorko and Langel 2022). Our findings corroborate a recent study of a peptide derived from the dengue virus capsid protein, PepR, which demonstrated rapid bactericidal activity against *Staphylococcus aureus* (Pinto et al. 2019). Furthermore, several authors deliberated that the viral protein-derived peptide (vAMP 059) and viral protein-derived CPPs (vCPP 0769 and vCPP 2319) are all bactericidal in nature and derived from viral capsids (Dias et al. 2017; Freire et al. 2015).

Intact cell membrane, metabolic activity, and reproducibility are the three accepted general parameters for the viability assessment of microorganisms (Oliver 2005). The conventional colony-forming unit (CFU) counting method takes into account only one of these parameters and reproducibility and provides only information about viable and culturable cells. The method is prone to errors due to the factors involved in bacterial growth on agar plates (Davis 2014). However, an alternative to culture-based detection is the assessment of cell viability using fluorescent dyes (LIVE/DEAD staining), including membrane potential and integrity that is being employed to determine bacterial viability (Berney et al. 2007; Stiefel et al. 2015). LIVE/DEAD staining permits detection of cell states other than live and dead, as those unable to grow, including live injured (where membrane integrity is compromised) cells that are unable to grow on agar plates (Léonard et al. 2016; Stiefel et al. 2015). Furthermore, it allows the loss of membrane integrity to be visualised over time (Nocker et al. 2017). Therefore, LIVE/DEAD staining was performed in this study to look into the status of host cells after treatment with the ~30 kDa-purified putative EP of *Bl* 1821L. The staining displayed more *Bl* 1821L cells with compromised membranes (red) 6 to 12 h after treatment, and a pronounced effect was noted with an increase in the volume of purified putative EP. Interestingly, the appearance of red cells occurred at the same time as a decrease in the number of viable cells at 6 h after treatment with the ~30 kDa-purified putative EP as compared to the control. Typically, cells with compromised membranes (red) are considered to be dead or approaching death (Oliver 2010; Robertson et al. 2019). However, in this study, yellow/orange cells were also seen after LIVE/DEAD staining of host cells in the overlay images which have been included the category of dead cells in the past (Leuko et al. 2004; Lu et al. 2014; Stiefel et al. 2015). The cause might be insufficient replacement of SYTO9 with PI and the retention of both the dyes within the cells at the same time (Kirchhoff and Cypionka 2017; Lu et al. 2014). A decrease in the colony count and a concurrent increase in the number of cells with compromised cell membranes (red) in LIVE/DEAD staining assay suggests that the CPPs residing in the ~30 kDa-purified putative EP may be involved in the formation of pores to lyse the susceptible cells (Sanderson 2005).

The Gram-positive bacterium, *Bs*, is an effective expression system for foreign proteins due to the structural differences in the outer cell membrane compared to the Gram-negative bacterium, *E. coli* (Su et al. 2020; Zweers et al. 2008). The gene *lin* encoding the Linocin 18 protein (bacteriocin) from the bacterium *Br. linens* M18 has been successfully expressed in *E. coli* (Valdes-Stauber and Scherer 1996). However, to enhance the stability of secreted proteins, an extracellular protease deficient mutant *Bs* WB800N (Jeong et al. 2018; Nguyen et al. 2011) was used in this study. The role of genes encoding the 25 kDa hypothetical and 31.4 kDa putative EP (Linocin M18) in the antagonistic activity was affirmed by expressing the corresponding genes individually (pHT01-*hypo*, 25 kDa), (pHT01-*encap*, 31.4 kDa) and together pHT01-*hypo*.*encap* in *Bs* WB800N. Notably, in the bioactivity assays, each expressed hypothetical (pHT01-*hypo*), encapsulating (pHT01-*encap*), and pHT01-*hypo*.*encap* encoding both the proteins expressed their antagonistic activity. BLASTp analysis of 25 kDa hypothetical protein shared 98.6% amino acid identity to a hypothetical protein of *Brevibacterium* sp. JNUCC-42. Interestingly, the CFS of heterologously expressed 25 kDa hypothetical protein (pHT01-*hypo*) exhibited more potent activity as compared to 31.4 kDa putative EP (pHT01-*encap*), but SDS-PAGE analysis showed only purified bands of ≥50 kDa. The absence of the expected protein band might be due to the loss of expressed protein in the multiple purification steps (Walker 2010) or low expression of the proteins of interest (Lu et al. 2012). The putative EP (31.4 kDa) of *Bl* 1821L was successfully expressed in *Bs* WB800N at low levels. Bioactivity assay of *Bs* WB800N (pHT01-*encap*) supernatant containing 31.4 kDa protein exhibited antibacterial activity against *Bl* 1821L, and a prominent purified band of ~30 kDa was visualised on SDS-PAGE. However, activity of the 31.4 kDa protein (pHT01-*encap*, B1) was found against both strains *Bl* 1821L and *Bl* 1951, which might be due to the variation in the expression of pHT01-*encap*, B1 and pHT01-*encap*, and B2. Notably, the expression of both the encoded 25 kDa hypothetical and 31.4 kDa putative encapsulating proteins in *Bs* WB800N (pHT01-*hypo*.*encap*) displayed an inhibitory action against both the strains (*Bl* 1821L and *Bl* 1951), and purification of the corresponding supernatant yielded a protein band of ~30 kDa along with other proteins. All the constructs used in this study expressed the antibacterial activity in *Bs* WB800N, which is otherwise absent in this strain. Based on the literature, this is not only the first report of the involvement of family 1 encapsulin nanocompartment shell protein (Linocin M18) from the genus *Brevibacillus* in the putative antibacterial activity, but also among the entomopathogenic bacteria.

Overall, this research identified, purified, and characterised homologous genes encoding the 31.4 kDa family 1 encapsulin nanocompartment shell protein (Linocin M18) in *Bl* 1821L and *Bl* 1951 genomes. In addition, a gene encoding the more potent 25 kDa hypothetical protein was also identified at 3′ end of the identified putative EP. Antagonistic activity of the crude and purified lysates differed remarkably. The ~30 kDa-purified putative EP of *Bl* 1951 and *Bl* 1821L was found to be active against both the isolates, but the autocidal activity was more pronounced with the crude lysates of both the strains. The autocidal activity of the identified 31.4 kDa putative EP (Linocin M18) may pose implications in harnessing the insecticidal potential of these isolates in future. Furthermore, based on bioinformatics analyses, this study proposed different killing mechanisms of 31.4 kDa putative EP of *Bl* 1821L and *Bl* 1951. These include the activation of stress relevant transcriptional regulator family proteins (PadR and MarR) or YtxJ protein under some stresses (such as malnutrition of iron), failure of ferritin protein to detoxify iron, and cell-penetrating peptide activity. However, these likely mechanisms require further investigation to validate their mechanistic role in the stunted growth of *Bl* 1821L and *Bl* 1951strains.

## Supplementary information

ESM 1 The online version contains supplementary material available at https://doi.org/xxx/.

## Data Availability

All data generated or analysed during this study are included in this published article and its supplementary information file. The insect pathogenic strains *Bl* 1951 and *Bl* 1821L are deposited in the National Measurement Institute, Melbourne, Australia with the accession NMI No. V12/0001945 (*Bl* 1951) and NMI No. V12/0001946 (*Bl* 1821L). Sequences of *Bl* 1951 (GenBank accession RHPK01000003.1, contig 1) and *Bl* 1821L (GenBank accession NZ_CP033464.1) are deposited under the Bioproject accession number PRJNA503267 in the National Centre for Biotechnology Information (NCBI) database.

## References

[CR1] Åberg A, Nordlund P, Eklund H (1993). Unusual clustering of carboxyl side chains in the core of iron-free ribonucleotide reductase. Nature.

[CR2] Akita F, Chong KT, Tanaka H, Yamashita E, Miyazaki N, Nakaishi Y, Suzuki M, Namba K, Ono Y, Tsukihara T (2007). The crystal structure of a virus-like particle from the hyperthermophilic archaeon *Pyrococcus furiosus* provides insight into the evolution of viruses. J Mol Biol.

[CR3] Almeida AV, Carvalho AJ, Pereira AS (2021). Encapsulin nanocages: protein encapsulation and iron sequestration. Coord Chem Rev.

[CR4] Al-Sammak EG (2014). Identification of bacteriocin Linocin M18 from *Brevibacterium* and related genera using PCR. J Biotech Res Center.

[CR5] Anderson I, Sorokin A, Kapatral V, Reznik G, Bhattacharya A, Mikhailova N, Burd H, Joukov V, Kaznadzey D, Walunas T (2005). Comparative genome analysis of *Bacillus cereus* group genomes with *Bacillus subtilis*. FEMS Microbiol Lett.

[CR6] Andreas MP, Giessen TW (2021). Large-scale computational discovery and analysis of virus-derived microbial nanocompartments. Nat Commun.

[CR7] Andrews NC (2010). Ferrit (in) ing out new mechanisms in iron homeostasis. Cell Metab.

[CR8] Babar TK (2021). Heads or tails? An insight into the nature of antibacterial structures of an entomopathogenic bacterium *Brevibacillus laterosporus*.

[CR9] Babar TK, Glare TR, Hampton JG, Hurst MRH, Narciso JO (2022). Isolation, purification, and characterisation of a phage tail-like bacteriocin from the insect pathogenic bacterium *Brevibacillus laterosporus*. Biomolecules.

[CR10] Babar TK, Glare TR, Hampton JG, Hurst MRH, Narciso JO, Beattie A (2022b, 1932) Purification of high-molecular-weight antibacterial proteins of insect pathogenic *Brevibacillus laterosporus* isolates. Processes 10(10)

[CR11] Baum JA, CaJacob CA, Feldman P, Heck GR, Nooren IMA, Plaetinck G, Maddelein WT, Vaughn TT (2020) Methods for genetic control of insect infestations in plants and compositions thereof. Google Patents. U.S. Patent No. 10, 538, 783

[CR12] Bedini S, Muniz ER, Tani C, Conti B, Ruiu L (2020). Insecticidal potential of *Brevibacillus laterosporus* against dipteran pest species in a wide ecological range. J Invertebr Pathol.

[CR13] Berney M, Hammes F, Bosshard F, Weilenmann H-U, Egli T (2007) Assessment and interpretation of bacterial viability by using the LIVE/DEAD BacLight Kit in combination with flow cytometry. App Environ Microbiol 73(10):3283–3290 10.1128/AEM.02750-06 PMC1907116 17384309

[CR14] Blum H, Beier H, Gross HJ (1987). Improved silver staining of plant proteins, RNA and DNA in polyacrylamide gels. Electrophoresis.

[CR15] Boets A, Arnaut G, Van Rie J, Damme N (2011) Toxins. Google Patents. U.S. Patent No. 7,919,609

[CR16] Bradley JM, Moore GR, Le Brun NE (2014). Mechanisms of iron mineralization in ferritins: one size does not fit all. J Biol Inorg Chem.

[CR17] Bradley JM, Svistunenko DA, Wilson MT, Hemmings AM, Moore GR, Le Brun NE (2020). Bacterial iron detoxification at the molecular level. J Biol Chem.

[CR18] Carramaschi IN, Pereira LA, Queiroz MMC, Zahner V (2015). Preliminary screening of the larvicidal effect of *Brevibacillus laterosporus* strains against the blowfly *Chrysomya megacephala* (Fabricius, 1794)(Diptera: Calliphoridae). Rev Soc Bra Med Trop.

[CR19] Chmelyuk NS, Oda VV, Gabashvili AN, Abakumov MA (2023). Encapsulins: structure, properties, and biotechnological applications. Biochem (Mosc).

[CR20] Cole S, Brosch R, Parkhill J, Garnier T, Churcher C, Harris D, Gordon S, Eiglmeier K, Gas S, Cr B (1998). Deciphering the biology of *Mycobacterium tuberculosis* from the complete genome sequence. Nature.

[CR21] Davis C (2014). Enumeration of probiotic strains: review of culture-dependent and alternative techniques to quantify viable bacteria. J Microbiol Methods.

[CR22] De Oliveira EJ, Rabinovitch L, Monnerat RG, Passos LKJ, Zahner V (2004). Molecular characterisation of *Brevibacillus laterosporus* and its potential use in biological control. Appl Environ Microbiol.

[CR23] Dias SA, Freire JM, Pérez-Peinado C, Domingues MM, Gaspar D, Vale N, Gomes P, Andreu D, Henriques ST, Castanho MA (2017). New potent membrane-targeting antibacterial peptides from viral capsid proteins. Front Microbiol.

[CR24] Duda RL, Teschke CM (2019). The amazing HK97 fold: versatile results of modest differences. Curr Opin Virol.

[CR25] Eppert I, Valdés-Stauber N, Götz H, Busse M, Scherer S (1997). Growth reduction of *Listeria* spp. caused by undefined industrial red smear cheese cultures and bacteriocin-producing *Brevibacterium* lines as evaluated in situ on soft cheese. Appl Environ Microbiol.

[CR26] Fagundes PC, Ceotto H, Potter A, de Paiva Brito MAV, Brede D, Nes IF, MDC d FB (2011). Hyicin 3682, a bioactive peptide produced by *Staphylococcus hyicus* 3682 with potential applications for food preservation. Res Microbiol.

[CR27] Floris I, Ruiu L, Satta A, Delrio G, Rubino S, Paglietti B, Ellar DJ, Pantaleoni RA (2008) *Brevibacillus laterosporus* strain compositions containing the same and method for the biological control of dipters. Google Patents. Switzerland. Patent No. WO 2008/031887 A2

[CR28] Freire JM, Almeida Dias S, Flores L, Veiga AS, Castanho MA (2015). Mining viral proteins for antimicrobial and cell-penetrating drug delivery peptides. Bioinformatics.

[CR29] Gabashvili AN, Chmelyuk NS, Efremova MV, Malinovskaya JA, Semkina AS, Abakumov MA (2020). Encapsulins-bacterial protein nanocompartments: structure, properties, and application. Biomolecules.

[CR30] Gautam A, Chaudhary K, Kumar R, Sharma A, Kapoor P, Tyagi A, Raghava GP (2013) In silico approaches for designing highly effective cell penetrating peptides. J Trans Med 11(1):1-12 10.1186/1479-5876-11-74 PMC3615965 23517638

[CR31] Ghequire MG, De Mot R (2015). The tailocin tale: peeling off phage tails. Trends Microbiol.

[CR32] Giessen TW (2022). Encapsulins. Ann Rev Biochem.

[CR33] Giessen TW, Orlando BJ, Verdegaal AA, Chambers MG, Gardener J, Bell DC, Birrane G, Liao M, Silver PA (2019). Large protein organelles form a new iron sequestration system with high storage capacity. e-Life.

[CR34] Giessen TW, Silver PA (2016). Encapsulation as a strategy for the design of biological compartmentalization. J Mol Biol.

[CR35] Giessen TW, Silver PA (2017). Widespread distribution of encapsulin nanocompartments reveals functional diversity. Nat Microbiol.

[CR36] Glare TR, Durrant A, Berry C, Palma L, Ormskirk MM, Cox MP (2020). Phylogenetic determinants of toxin gene distribution in genomes of *Brevibacillus laterosporus*. Genomics.

[CR37] Glare TR, Hampton JG, Cox MP, Bienkowski DA (2014) Novel strains of *Brevibacillus laterosporus* as biocontrol agents against plant pests, particularly lepidoptera and diptera. Switzerland. Patent No. WO 2014/045131

[CR38] Glare TR, Hampton JG, Cox MP, Bienkowski DA (2018) Biocontrol compositions. Google Patents. U.S. Patent No. 10, 004,236 B2

[CR39] González-Pastor JE (2011). Cannibalism: a social behavior in sporulating *Bacillus subtilis*. FEMS Microbiol Rev.

[CR40] Grant R, Filman D, Finkel S, Kolter R, Hogle J (1998). The crystal structure of Dps, a ferritin homolog that binds and protects DNA. Nat Struct Biol.

[CR41] Hamze R, Ruiu L (2022). Brevibacillus laterosporus as a natural biological control agent of soil-dwelling nematodes. Agronomy.

[CR42] Hayes F, Kędzierska B (2014). Regulating toxin-antitoxin expression: controlled detonation of intracellular molecular timebombs. Toxins.

[CR43] He D, Hughes S, Vanden-Hehir S, Georgiev A, Altenbach K, Tarrant E, Mackay CL, Waldron KJ, Clarke DJ, Marles-Wright J (2016). Structural characterisation of encapsulated ferritin provides insight into iron storage in bacterial nanocompartments. eLife.

[CR44] Hicks PM, Rinker KD, Baker JR, Kelly RM (1998). Homomultimeric protease in the hyperthermophilic bacterium *Thermotoga maritima* has structural and amino acid sequence homology to bacteriocins in mesophilic bacteria. FEBS Lett.

[CR45] Höfler C, Heckmann J, Fritsch A, Popp P, Gebhard S, Fritz G, Mascher T (2016). Cannibalism stress response in *Bacillus subtilis*. Microbiol.

[CR46] Järver P, Langel Ü (2006). Cell penetrating peptides- a brief introduction. Biochim Biophys Acta- Biomembr.

[CR47] Jeong H, Jeong DE, Park SH, Kim SJ, Choi SK (2018) Complete genome sequence of *Bacillus subtilis* strain WB800N, an extracellular protease-deficient derivative of strain 168. Microbiol Resour Announc 7(18). 10.1128/mra.01380-18 10.1128/MRA.01380-18 PMC6256541 30533776

[CR48] Jones JA, Giessen TW (2021). Advances in encapsulin nanocompartment biology and engineering. Biotech Bbioeng.

[CR49] Karas VO, Westerlaken I, Meyer AS (2015). The DNA-binding protein from starved cells (Dps) utilises dual functions to defend cells against multiple stresses. J Bacteriol.

[CR50] Kawamoto S, Watanabe M, Saito N, Hesketh A, Vachalova K, Matsubara K, Ochi K (2001). Molecular and functional analyses of the gene (eshA) encoding the 52-kilodalton protein of *Streptomyces coelicolor* A3 (2) required for antibiotic production. J Bacteriol.

[CR51] Kearse M, Moir R, Wilson A, Stones-Havas S, Cheung M, Sturrock S, Buxton S, Cooper A, Markowitz S, Duran C (2012). Geneious Basic: an integrated and extendable desktop software platform for the organisation and analysis of sequence data. Bioinformatics.

[CR52] Kirby WM, Yoshihara GM, Sundsted KS, Warren JH (1956) Clinical usefulness of a single disc method for antibiotic sensitivity testing. Antibiot Annu:892–897 13425478

[CR53] Kirchhoff C, Cypionka H (2017). Propidium ion enters viable cells with high membrane potential during live-dead staining. J Microbiol Methods.

[CR54] Kouadio J-L, Duff S, Aikins M, Zheng M, Rydel T, Chen D, Bretsnyder E, Xia C, Zhang J, Milligan J (2021). Structural and functional characterisation of Mpp75Aa1. 1, a putative beta-pore forming protein from *Brevibacillus laterosporus* active against the western corn rootworm. PLoS One.

[CR55] Laemmli U (1970). SDS-PAGE Laemmli method. Nature.

[CR56] Lamsa A, Liu WT, Dorrestein PC, Pogliano K (2012). The *Bacillus subtilis* cannibalism toxin SDP collapses the proton motive force and induces autolysis. Mol Microbiol.

[CR57] Léonard L, Bouarab Chibane L, Ouled Bouhedda B, Degraeve P, Oulahal N (2016). Recent advances on multi-parameter flow cytometry to characterise antimicrobial treatments. Fron Microbiol.

[CR58] Leuko S, Legat A, Fendrihan S, Stan-Lotter H (2004). Evaluation of the LIVE/DEAD Bac Light kit for detection of extremophilic archaea and visualisation of microorganisms in environmental hypersaline samples. App Environ Microbiol.

[CR59] Lu J, Turnbull L, Burke CM, Liu M, Carter DA, Schlothauer RC, Whitchurch CB, Harry EJ (2014). Manuka-type honeys can eradicate biofilms produced by *Staphylococcus aureus* strains with different biofilm-forming abilities. P Journal.

[CR60] Lu YP, Zhang C, Lv F, Bie X, Lu ZX (2012). Study on the electro-transformation conditions of improving transformation efficiency for *Bacillus subtilis*. Lett App Microbiol.

[CR61] McDowell HB, Hoiczyk E (2022). Bacterial nanocompartments: structures, functions, and applications. J Bacteriol.

[CR62] McHugh CA, Fontana J, Nemecek D, Cheng N, Aksyuk AA, Heymann JB, Winkler DC, Lam AS, Wall JS, Steven AC (2014). A virus capsid-like nanocompartment that stores iron and protects bacteria from oxidative stress. The EMBO J.

[CR63] Medema MH, Blin K, Cimermancic P, de Jager V, Zakrzewski P, Fischbach MA, Weber T, Takano E, Breitling R (2011) antiSMASH: rapid identification, annotation and analysis of secondary metabolite biosynthesis gene clusters in bacterial and fungal genome sequences. Nucleic Acids Res 39 (Web Server issue):W339-46. 10.1093/nar/gkr466 PMC3125804 21672958

[CR64] Miljkovic M, Jovanovic S, O'Connor PM, Mirkovic N, Jovcic B, Filipic B, Dinic M, Studholme DJ, Fira D, Cotter PD, Kojic M (2019). Brevibacillus laterosporus strains BGSP7, BGSP9, and BGSP11 isolated from silage produce broad spectrum multi-antimicrobials. PLoS One.

[CR65] Milletti F (2012). Cell penetrating peptides: classes, origin, and current landscape. Drug Discov Today.

[CR66] Mistry J, Chuguransky S, Williams L, Qureshi M, Salazar GA, Sonnhammer EL, Tosatto SC, Paladin L, Raj S, Richardson LJ (2021). Pfam: the protein families database in 2021. Nucleic Acids Res.

[CR67] Nguyen TKC, Tran NP, Cavin J-F (2011). Genetic and biochemical analysis of PadR-padC promoter interactions during the phenolic acid stress response in *Bacillus subtilis* 168. J Bacteriol.

[CR68] Nichols RJ, Cassidy-Amstutz C, Chaijarasphong T, Savage DF (2017). Encapsulins: molecular biology of the shell. Crit Rev Biochem Mol Biol.

[CR69] Nocker A, Cheswick R, Dutheil de la Rochere P-M, Denis M, Léziart T, Jarvis P (2017). When are bacteria dead? A step towards interpreting flow cytometry profiles after chlorine disinfection and membrane integrity staining. Environ Tech.

[CR70] Oliver JD (2005). The viable but nonculturable state in bacteria. J Microbiol.

[CR71] Oliver JD (2010). Recent findings on the viable but nonculturable state in pathogenic bacteria. FEMS Microbiol Rev.

[CR72] Orban K, Finkel SE (2022) Dps is a universally conserved dual-action DNA-binding and ferritin protein. J Bacteriol e00036-22 10.1128/jb.00036-22 PMC9112962 35380871

[CR73] Orlova E, White H, Sherman M, Brasilès S, Tavares P, Jacquet E, Seavers P (2012). Capsid structure and its stability at the late stages of bacteriophage SPP1 assembly. Microsc Microan.

[CR74] Pinto SN, Dias SA, Cruz AF, Mil-Homens D, Fernandes F, Valle J, Andreu D, Prieto M, Castanho MA, Coutinho A (2019). The mechanism of action of pepR, a viral-derived peptide, against *Staphylococcus aureus* biofilms. J Antimicrob Chemother.

[CR75] Popp PF, Mascher T (2019). Coordinated cell death in isogenic bacterial populations: sacrificing some for the benefit of many?. J Mol Biol.

[CR76] Reddy PV, Puri RV, Khera A, Tyagi AK (2012). Iron storage proteins are essential for the survival and pathogenesis of *Mycobacterium tuberculosis* in THP-1 macrophages and the guinea pig model of infection. J Bacteriol.

[CR77] Riley MA, Wertz JE (2002). Bacteriocins: evolution, ecology, and application. Ann Rev Microbiol.

[CR78] Robertson J, McGoverin C, Vanholsbeeck F, Swift S (2019). Optimisation of the protocol for the LIVE/DEAD® BacLightTM bacterial viability kit for rapid determination of bacterial load. Front Microbiol.

[CR79] Rosenkrands I, Rasmussen PB, Carnio M, Jacobsen S, Theisen M, Andersen P (1998). Identification and characterisation of a 29-kilodalton protein from *Mycobacterium tuberculosis* culture filtrate recognised by mouse memory effector cells. Infect Immun.

[CR80] Rybakova D, Mitra AK, Hurst MRH (2014). Purification and TEM of Afp and Its variants. Bio-Protoc.

[CR81] Salama H, Foda M, El-Bendary M, Abdel-Razek A (2004). Infection of red palm weevil, *Rhynchophorus ferrugineus*, by spore-forming bacilli indigenous to its natural habitat in Egypt. J Pest Sci.

[CR82] Sampson KS, Tomso DJ, Guo R (2016) Pesticidal genes from *Brevibacillus* and methods for their use. Google Patents. U.S. Patent No. 9,238,823 B2

[CR83] Sanderson JM (2005). Peptide-lipid interactions: insights and perspectives. Organ Biomol Chem.

[CR84] Shida O, Takagi H, Kadowaki K, Komagata K (1996). Proposal for two new genera, *Brevibacillus* gen. nov. and *Aneurinibacillus* gen. nov. Int J Syst Evol Microbiol.

[CR85] Singh PK, Sharma V, Patil PB, Korpole S (2012). Identification, purification and characterisation of laterosporulin, a novel bacteriocin produced by *Brevibacillus* sp. strain GI-9. PloS One.

[CR86] Stiefel P, Schmidt-Emrich S, Maniura-Weber K, Ren Q (2015). Critical aspects of using bacterial cell viability assays with the fluorophores SYTO9 and propidium iodide. BMC Microbiol.

[CR87] Su Y, Liu C, Fang H, Zhang D (2020). Bacillus subtilis: a universal cell factory for industry, agriculture, biomaterials and medicine. Microb Cell Factories.

[CR88] Suhanovsky MM, Teschke CM (2015). Nature′ s favorite building block: deciphering folding and capsid assembly of proteins with the HK97-fold. Virology.

[CR89] Sutter M, Boehringer D, Gutmann S, Günther S, Prangishvili D, Loessner MJ, Stetter KO, Weber-Ban E, Ban N (2008). Structural basis of enzyme encapsulation into a bacterial nanocompartment. Nat Struct Mol Biol.

[CR90] Terlouw BR, Blin K, Navarro-Muñoz JC, Avalon NE, Chevrette MG, Egbert S, Lee S, Meijer D, Recchia MJ, Reitz ZL (2023). MIBiG 3.0: a community-driven effort to annotate experimentally validated biosynthetic gene clusters. Nucleic Acids Res.

[CR91] Theil EC, Behera RK, Tosha T (2013). Ferritins for chemistry and for life. Coord Chem Rev.

[CR92] Torrent M, Di Tommaso P, Pulido D, Nogués MV, Notredame C, Boix E, Andreu D (2012). AMPA: an automated web server for prediction of protein antimicrobial regions. Bioinformatics.

[CR93] Valdés-Stauber N, Scherer S (1994) Isolation and characterisation of Linocin M18, a bacteriocin produced by *Brevibacterium linens*. App Environ Microbiol 60(10):3809–3814. 10.1128/aem.60.10.3809-3814.1994 10.1128/aem.60.10.3809-3814.1994 PMC201890 7986050

[CR94] Valdes-Stauber N, Scherer S (1996) Nucleotide sequence and taxonomical distribution of the bacteriocin gene *lin *cloned from *Brevibacterium linens* M18. Appl Environ Microbiol 62(4):1283–1286. 10.1128/aem.62.4.1283-1286.1996 10.1128/aem.62.4.1283-1286.1996 PMC167894 8919789

[CR95] van Heel AJ, de Jong A, Song C, Viel JH, Kok J, Kuipers OP (2018). BAGEL4: a user-friendly web server to thoroughly mine RiPPs and bacteriocins. Nucleic Acids Res.

[CR96] van Zijll de Jong E, Roush TL, Glare TR, Hampton JG (2016). Discovery of two *Brevibacillus laterosporus* isolates from brassica with insecticidal properties against diamondback moth. Biocont Sci Tech.

[CR97] Walker J, Wilson K, Walker J (2010). Proteins structure, purification, characterisation, and functional analysis. Principles and techniques of biochemistry and molecular biology.

[CR98] Wikoff WR, Liljas L, Duda RL, Tsuruta H, Hendrix RW, Johnson JE (2000). Topologically linked protein rings in the bacteriophage HK97 capsid. Science.

[CR99] Winter N, Triccas JA, Rivoire B, Pessolani MCV, Eiglmeier K, Lim E-M, Hunter SW, Brennan PJ, Britton WJ (1995). Characterisation of the gene encoding the immunodominant 35 kDa protein of *Mycobacterium leprae*. Mol Microbiol.

[CR100] Wiryaman T, Toor N (2022) Recent advances in the structural biology of encapsulin bacterial nanocompartments. J Struct Bio:100062. 10.1016/j.yjsbx.2022.100062 10.1016/j.yjsbx.2022.100062 PMC8802124 35146412

[CR101] Wu S-C, Yeung JC, Duan Y, Ye R, Szarka SJ, Habibi HR, Wong S-L (2002). Functional production and characterisation of a fibrin-specific single-chain antibody fragment from *Bacillus subtilis*: effects of molecular chaperones and a wall-bound protease on antibody fragment production. Appl Eviron Microbiol.

[CR102] Yasumitsu H, Ozeki Y, Kawsar SM, Fujii Y, Sakagami M, Matuo Y, Toda T, Katsuno H (2010). RAMA stain: a fast, sensitive and less protein-modifying CBB R250 stain. Electrophoresis.

[CR103] Zorko M, Langel Ü, Langel Ü (2022). Cell-penetrating peptides. Cell penetrating peptides: methods and protocols.

[CR104] Zweers JC, Barák I, Becher D, Driessen AJ, Hecker M, Kontinen VP, Saller MJ, Vavrová L, van Dijl JM (2008). Towards the development of *Bacillus subtilis* as a cell factory for membrane proteins and protein complexes. Microb Cell Factories.

