# Supplementary material for: Linocin M18 protein from the insect pathogenic bacterium Brevibacillus laterosporus isolates
Source: Appl Microbiol Biotechnol. 2023 May 19;107(13):4337–53. doi: 10.1007/s00253-023-12563-8 (PMC10313851; doi:10.1007/s00253-023-12563-8)
Supplement: Supplementary file 1 — The online version contains supplementary material available at https://doi.org/xxx/. [file 253_2023_12563_MOESM1_ESM.pdf]

Supplementary Information

Applied Microbiology and Biotechnology

Linocin M18 protein from the insect pathogenic bacterium *Brevibacillus laterosporus* isolates

Tauseef K. Babar*[1,2], Travis R. Glare[1,3], John G. Hampton[1,3], Mark R. H. Hurst[4], Josefina Narciso[1,3], Campbell R. Sheen[5], Barbara Koch[5]

[1] Bio-Protection Research Centre, Lincoln University, Lincoln 7647, Canterbury, New Zealand
[2] Department of Entomology, Faculty of Agricultural Sciences and Technology, Bahauddin Zakariya University, Multan 60000, Pakistan
[3] Faculty of Agriculture and Life Sciences, Lincoln University, Lincoln 7647, Canterbury, New Zealand
[4] Resilient agriculture, AgResearch, Lincoln Research Centre, Christchurch, New Zealand
[5] Protein Science and Engineering, Callaghan Innovation, Christchurch, New Zealand

Author for correspondence: Dr. Tauseef Khan Babar, Assistant Professor, Department of Entomology, Faculty of Agricultural Sciences and Technology, Bahauddin Zakariya University, Multan 60000, Pakistan

E-mail: tauseefkhan@bzu.edu.pk

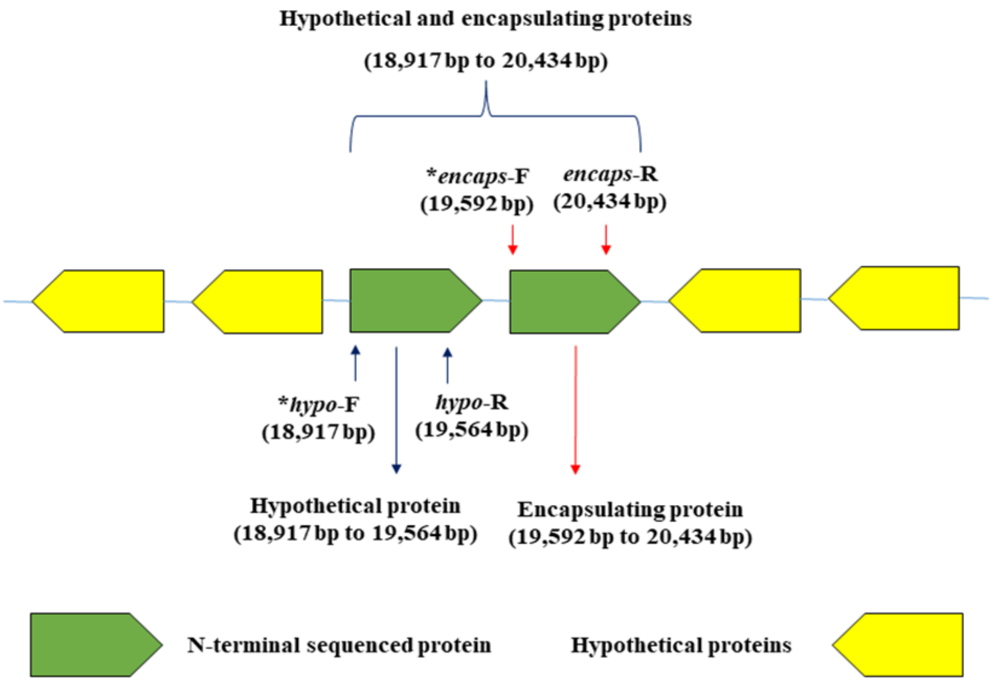

Fig. S1 Schematic of genes encoding the *Bl* 1821L proteins which correspond to the constructs used in this study for expression in *Bacillus subtilis* WB800N. Genes encoding the 25 kDa hypothetical protein (GenBank accession WP_113757161, shown with blue arrow) and 31.4 kDa putative encapsulating protein (GenBank accession WP_113757162, shown with red arrows) are shown in green colours

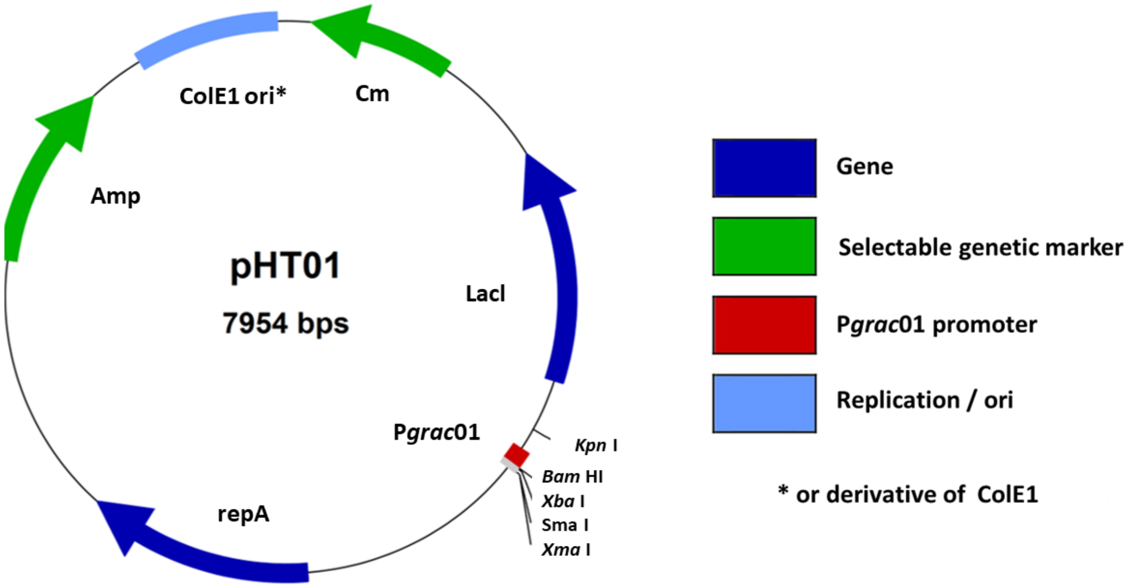

Fig. S2 Schematic presentation of plasmid pHT01 used in this study with promoter P*grac*01 and associated restriction enzyme sites

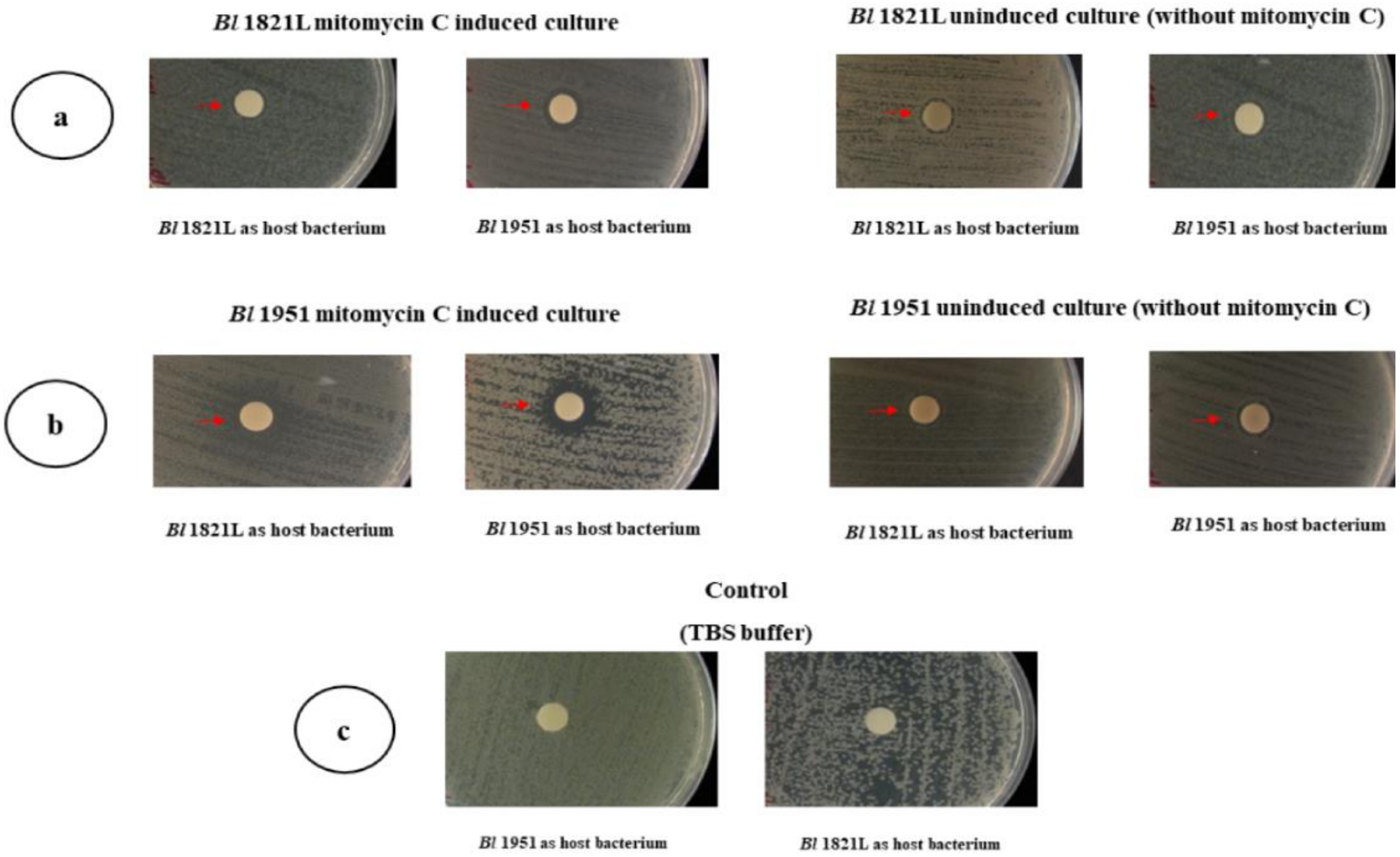

Fig. S3 Disc diffusion assay test of *Bl* 1821L and *Bl* 1951 cultures with/without mitomycin C addition and control treatment against the producer strain and vice versa. Red arrows denote the zone of inhibition produced on the lawn of host bacterium due to the bioactivity of putative antibacterial proteins

Note: For the bioactivity assay 80 µL of mitomycin C- induced cell free supernatant (CFS) was pipetted on the paper disc (8 mm), the negative control comprised the same volume of tris buffer saline (TBS).

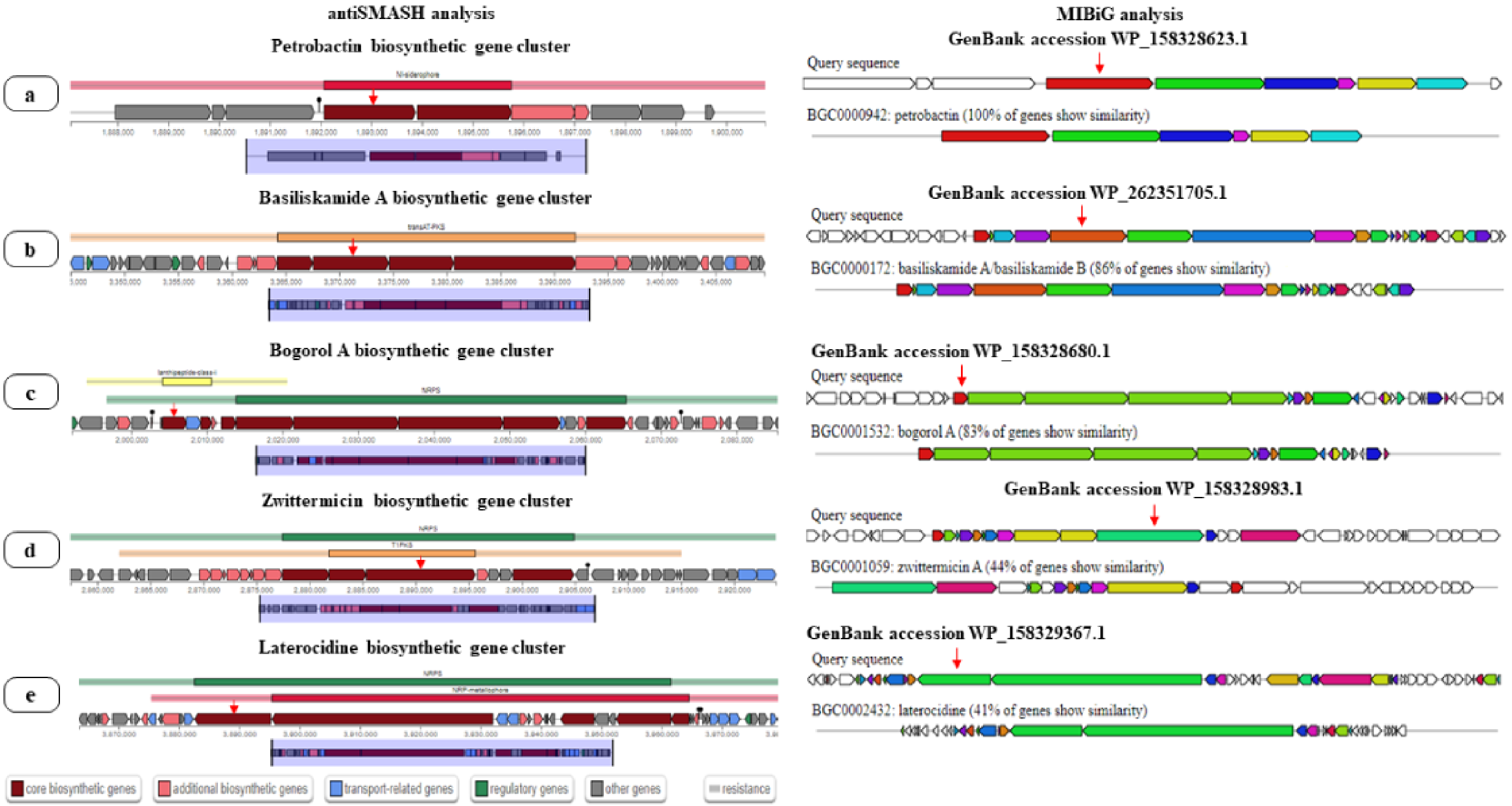

Fig. S4 antiSMASH and MIBiG analysis of the *Bl* 1821L genome *Bl* 1821L (NZ_CP033464.1)

Note: antiSMASH analysis identified the potential gene clusters of *Bl* 1821L (NZ_CP033464.1) associated with the production of various secondary metabolites including petrobactin, basiliskamide A, bogorol A, zwittermicin, and laterocidine. The predicted gene clusters were compared with the known gene cluster of different bacteria using the programme MIBiG. For MIBiG analysis, the *Bl* 1821L genome served as the query sequence and the BGC 0000942, BGC 0000172, BGC 0001532, BGC 0001059, and BGC 0002432 were the MIBiG accessions belonging to the similar known gene clusters of the bacteria *Bacillus anthracis* strain Ames (petrobactin), *Bl* PE36 (basiliskamide), *Bl* DSM25 (bogorol A), *Bacillus cereus* UW85 (zwittermicin), and *Bl* LMG 15441 (laterocidine) respectively. Red arrow denotes the encoded protein sequence that was compared the similar proteins from the other bacteria (% amino acid identity is presented in Table S1).

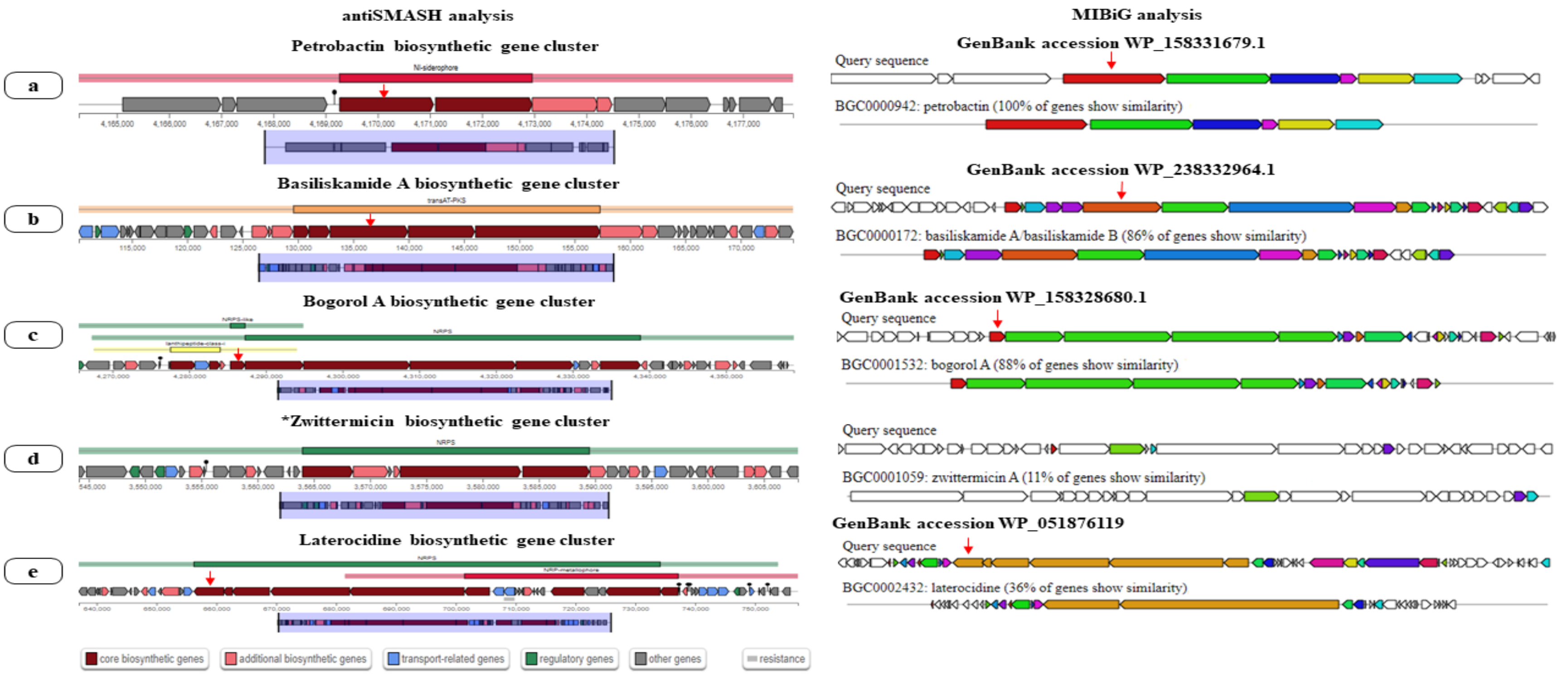

Fig. S5 antiSMASH and MIBiG analysis of *Bl* 1951 genome (RHPK01000003.1, contig 1)

Note: antiSMASH analysis identified the potential gene clusters of *Bl* 1951 genome (RHPK01000003.1, contig 1)associated with the production of various secondary metabolites including petrobactin, basiliskamide A, bogorol A, zwittermicin, and laterocidine. The predicted gene clusters were compared with the known gene cluster of different bacteria using the programme MIBiG. For MIBiG analysis, the *Bl* 1951genome served as the query sequence and the BGC 0000942, BGC 0000172, BGC 0001532, BGC 0001059, and BGC 0002432 were the MIBiG accessions belonging to the similar known gene clusters of the bacteria *Bacillus anthracis* strain Ames (petrobactin), *Bl* PE36 (basiliskamide), *Bl* DSM25 (bogorol A), *Bacillus cereus* UW85 (zwittermicin), and *Bl* LMG 15441 (laterocidine) respectively. Red arrow denotes the encoded protein sequence that was compared the similar proteins from the other bacteria (% amino acid identity is presented in Table S1).

* Zwittermicin biosynthetic gene cluster of *Bl* 1821L showed a low (11%) similarity with the known gene cluster of *B. cereus* UW85 therefore and no particular encoded protein was compared for % amino acid identity. However, an identical protein encoded (zwittermicin) in *Bl* 1821L was searched in *Bl* 1951genome and compared with the known gene cluster of *B. cereus* UW85 (*B. cereus* UW85 ((% amino acid identity is presented in Table S1).

Table. S1 Genome based prediction of antimicrobial molecules using bioinformatic tools (antiSMASH and BAGEL4)

**Bioinformatic tool**	**Antimicrobial molecule**	**Amino acid sequence**	**% amino acid identity of the predicted antimicrobial molecule of the *Bl* 1821L and *Bl* 1951 with the similar encoded protein in the gene cluster of other bacteria**
antiSMASH	Petrobactin	MKHVKDIAEHAAMQSFLNCYLRETGNGRWTTTNGEMHLHVPLVSQRMDLYVKASYQSPT<br>GRHLFDYPAFYQSGAEAPLVVADYVTMTVLLVKELSLQHGTKAVPDELVLRVIQSCQNM<br>ERYLAARYDDGEELYGFEMDFITAEQALLFGHLFHPTPKSRQGIPDAKQAVYSPEQKGA<br>FALHYFAAHHSLVLEGSGMDQSATEIIKTELGQGVLDLEGVKQFVSNDSYSLIPLHPLQ<br>AEWLLEQQPVQKYIKRGLLHNVGPLGESYQATSSLRTVYHPDKGFMIKLSMPVKVTNSL<br>RVNKLSELESGVEAKKVLDTFTKDMSKKFPGFAFIHDPAYITLKLDEQDESGFEVILRT<br>NPFQGSEAENTTVLAALIQDPLPGQQSRLAVIILQLARQEGRTPAEVSIDWFRRYLEIS<br>LKPMVWLYLKWGIGLEAHQQNSIVKLQDGYPSQFYYRDNQGYYYCQSMKELLSRSIPNL<br>GEKSRNVTEDSLVDERLRYYLIFNHMFGLINGFGSAGLIDEQQLLDEMRTVLQEFLPLN<br>REPSQFLQSLLTSEKLACKANLLTRFYDVDELESEEITSAIFVQIDNPLAKASQREKEL<br>ETLNQLALR	*Bl* 1821L= 55<br>*Bl* 1951= 55
antiSMASH	Bogorol A<br>(Non-ribosomal peptide synthetase NRPS)	MDLSTLNFLDEAEKHKLLNQFNDTDADFPQEMTIHALFEKQVQERPNHTAIIFKEQSMT<br>YKEINERANQVAHSLRKHGVIPDEIVGILADRNIDMIVAILGVLKAGAAYMPIDPTYPT<br>ERIHYMINDSQTKIVLVEDSEMSPEGCKVDLIFLHDPSLLEEETTNLLHVNKPEDLAYI<br>IYTSGSTGKPKGVMIEHCNVIRLLFNDRNLFDFNSEDVWTVFHSFCFDFSVWEMYGALL<br>NGGKIVLVSFEVARDPNAFRKLLQEQKVTILNQTPTAFYQLTSEEMQHSDGNLSIRKII<br>FGGEALTPSQLVAWKQKYPNTALINMYGITETTVHVTYKEFDLVDMGSTVSNIGKPIPT<br>LKTYVLDQRKNLVPIGVKGELYVSGKGVARGYLNKPELTEERFMENPFIPGERMYRTGD<br>LARWLPEGELEYLGRIDQQVKIRGYRIELGEIEAELLKLKGIKEAVLLVTNDKNEQPQL<br>QAYMTSTEDWSPSDLRIQLTTTLPSYMIPAHFIFVSHMPITSNGKIDKESLRKIEPTLQ<br>ESSTGMYVAPQTPTDQQLALIWEENIGMEPISIDNNYFALGGDSIKAIKLLHAINKEFQ<br>VNFQIGDLYKHGTIREMGQQIGEKGKQSSNQKQMKLQELERLKEKILGSDK	*Bl* 1821L= 90<br>*Bl* 1951= 90
antiSMASH	Basiliskamide A<br>Basiliskamide B<br>(Polyketide)	MLSILHLYSHGYLAIPVLLSCKKRGLFNLLQQSQCTPFKQIVRELNANSGHLRSALHML<br>ESLQIVSRNSEDAYALLTGWNVVEKIPEEIVELFDFPMKAYVKKSQRKHRLQKWIELSA<br>KQWNIDNVMYSHFLDGMLMIPLLFSLKEHGFVMQSSSQQKEFMFSNLSGIAKQEITQLF<br>LQKNWLDQGSLTEAGMYMMDRIFNTAIIYSYRPMIKNIDQLLFGDCQAVFDRDVQEHES<br>HIDRTLNVIGCGFQHEKYFKDMEQMIVSIFNRDSLEEQPKYIADMGCGDGSLLKKIYEI<br>IKMKSLRGQLLDQYPLTLIGIDYNEKSLIETAVTLCDIDHVLLKGDIGNPDELITSLEK<br>IGITDTENILHVRSFLDHDRPFIAPKEADEVNSFHSIPTETVFVDENGRERSSKLVMKN<br>LVEHLQLWSNVVNKHGLILLEVHCLEPKTINQYIDKCEGLHFDAVHRFSQQLLVEADTF<br>MLAAAEAGLFPHKQYFRKYPKSMSFARITLNYFEPREYRIRMAHEKDIPVLRQLELQCW<br>GEALGASSGNILHRIRNYPEGQLVLEIDNRVVGVMYSQKIRGLQDIKSAQTNTVEQLHH<br>NEGTIIQLLAINVLPEMQDRNLGDQLLEFMLQRCSLISGVRSVIGITRCKDYHRHRTVS	*Bl* 1821L= 70<br>*Bl* 1951= 70

		MDKYINFRNQNNKLIDKTLRFHEMHGAKIRELVPNYRPLDHANLGYGVLIEYDNLNRGY<br>SETHDKARKQVNLEKNFNPIKNGNTDLTRSITDFVLDRIRSLLGGNKEDVLSYVDRPLM<br>ELGLDSGDLLELNDQLGTKFQIELEATFFFTYNTARKIISYFIE	
antiSMASH	Zwittermicin A<br>(Non-ribosomal peptide, Polyketide)	LEIAVIGIAGRFPGADNLEQYWENLKNSVETISVFTDEELRMAGIDPALLQKPNYVKAK<br>GYIEDIELFDPGFFGYSPREVEIMDPQIRLLQECSWQALEDAGYDPKSYEGLIGLYVGA<br>ASNYEWVAQSPLMKNDGGAELHEAGTLCYKDAISTLTSYKLGLKGPSFTMYTACSTSML<br>SIHLACRALLTGECHIALAGGVRVSYPNKKGYLYEQGLTSSPDGHVRAFDADAGGAVFS<br>DGVGMVVLKRLEDAIADGDNISAVIKGSAVNNDGARKVGYTAPSVEGQSEVILAAQHLA<br>EVEPESISYIETHGTATNLGDTIEFEALKRAFQTEQKSFCGIGSVKSNIGHLDTAAGVA<br>GFLKTVLALKHKQLPPTLHFNRPNPRINLVNSPFYVVKGLQDWEHEGYPLRAGVSAFGY<br>GGTNVHVILEEAPIIESECSQSDQYVFPLSARSLTALDNAANQLADYLSNQLKVELADV<br>AYTLQVGRREFPYRRAIVATNREEAIDSLLARKGNATLAVVHQPDVIFMFHKHGFSDVT<br>FLLDLYSSEPTFKNALDRCFDQVQMITGYDLRNVLFPQYNLDDIALVLKQPEISEVVSF<br>VLQYALASLFIARGVAPTAMIGFGIGEYVSAVLGEVMTLEDVLSLVTARGKVIYSQTAT<br>VDDFQRLAQDIRLQSPRLPFISSVTGTWINDQQATDYGYWVRQIYEQADEGIGLEEVAK<br>NADVLFLQLFASSEQRNRLAKCLADREERIVSVIDQEEGNGETSQVYQSLGTLWSLGKT<br>PEWKSFYEGEKRCRVPLPTYPFEKQRYWPEQNSITTHTAMQKTQKLQKNENMAEWFYLP<br>TWEPSTFTAEKENKERRKYLIFTDEAGMGNKVANRLKETGHQVLTVEYGAAYSKKTERE<br>YVIQASEENDYKRLFQDLRNNDNLPQEIIHMWNVNKEDIGTASMELGYYSLIHLVKAIS<br>GQNMIAPLQISVITNHMQKVTDTDIVQPEKAMLLAPVKVIPQEFQNIRCRSIDMVETIE<br>QSLSQDAVIEQLFTELASEIVDTVVAYRNKERYVRTFNQVPVDQLAATLSLGIEETAPQ<br>LRKQGVYLITGGLGGIGTKLAEYLAKTVQAKLVLLGRSQLPPRTEWETWLANHEEKDDT<br>SLKIRHIQQMEEYGAEVLVISANVVDENQMRDVIAQAEEKFGAIHGVIHAAGILRVKSA<br>QCMMEKISKEECEEQFLPKLYGTLTLEKVLRDKPLDFCLLVSSLSPILGGLGFVAYAAA<br>NLYLDAFAEKQSVLNPNRWISVNWGDWQYTGREYEKTFVSESIELLEMTPTEGTKTFQC<br>VLGLGGVHQVIISSGNLYSRINQWIKLEALREEGPSVSVKSKASKQQNRIRNMENKLKI<br>SEIEQMISEIWMDFYRVDSVNPAESFFQLGATSLDIIQIHNKIVKTLERHIPIEVMFEF<br>PTIELLAKHLSGGEAVEEMEEVSKGNKNSTRKMSGDIAVIGMAGRFPGAQDIYAFWENL<br>KNGVESVVFFTDEELKEAGVDPAQSSLPNYVKAKGYLEGAEYFDSAFFDYTPKDAMLMD<br>PQLRVFHECTWAAFEHAGYNIDSYKGSVGVFAGASPNLYWQVMATLAEANEPSGQFLAS<br>LLNDKDSLSTQISYKLNLKGPSSNIFTGCSTSLVSIISACQALSNGQCDMAIAGGITLT<br>QPDKAGYIYEEGMLFAVDGHCRSFDEKASGMLFGDGVGVVVLKSLEDAVADGDFIHAVI<br>RGSAINNDGNRKVGYTAPSVEGQAEVIRAAHSSANVEPNSITYIETHGTATKLGDTIEI<br>SALKQAFNSEKRQYCAIGSVKSNVGHLNAASGVAGFIKVVMSMQNRQIPASLHFETPNK<br>QIDFDNSPFFVNTQLREWKSEEYPLRGGVSSFGIGGTNAHIVLEEASPKSISGDSLRGW<br>KMLVLSAKTVSSLDRMTQNLGNYLGKHPDVNLADVSYTLQIGRKPFRYRRTVLCQSVEE<br>ASELLLSIDTHKIDTVMAKGENRPVVFMFSGQGSQYVNMGRDLYEQEPQFRAEMDKCFA<br>ILEPMLGYDLRNVLFPTANEDEARTKINKNECGQPLLFIFEYALSQLLMQWGIQPSAVI<br>GYSFGEYVAATIAGVFTLQDVLKLIVERGRLMNKVSGAMLSVPLNAKETSARMADFITK<br>HSEADIRAISLAIDNGPSSIVSGSEKAIKEFELELRANRLLCMRVPSEHAAHSIVLDPI<br>LEDFEQMIRTIPLQEPEIPYISNVTGTWVTAEQVQDPMYWVTHMRETVRFADGIEVLKQ<br>DKDSVFVEIGPSRDLSVLLNRFFEGEPERILHTTKHPQQKISDVYFLLTKLARLWALGV	*Bl* 1821L= 53<br>*Bl* 1951= 53

		FVDWTNFYADEERQRIPLPTYSFEPTSYKIQANPFELSQRLTKKKPQLHKKENISEWFY<br>TPQWSSVPLRKAKGSESLAEQRWLIFADESGLGTKVATGIETVAKSVVIVKAGESFSKL<br>AENCYQLNLKEEDHYLKLLSELSKEEKLPTRIAHFLGVTTTEQNAASNLSDFQNRGFYS<br>LFHLVKSLVGKQTNDQIDIKVISNNVHEILGDETLVPEKATVLATAIVAPQEYSYLNCS<br>HIDIVSPDMGSKREEKLVEQLIAECCAPSSDKLIAYRGKNRWVQGYVSHTIDNVIENTS<br>PLRQKGVYLITGGLGGIGLILAEYLVKTVQAKLILTGRSGLPAHEDWKEWLETHDENNL<br>ITQKIRKLEELEQLGGEVLVCSCDVADKEQMQAVITQAEQLFGPINGVIHGAGVLGGNT<br>FNLIKELHVADCEEQFHSKMYGLRVLSEVLCDKQLDFCLLMSSIASVLGGLGFTAYSAA<br>NLYMDYFVQKYNKDALVPWISVNWSDWKYWNDAEKDTSVGGSIWELSMLPEEGVQAFHR<br>ILASGETWIANSPGDLQVRIDQWVHLQSLRSEEEDDTDITLLHARPALMSPYVEPRTEL<br>EKELVEIWQKIFRVNQIGIEDDFFELGGDSLKGITLVSRIHKELNVEIPVAQLFDTPTI<br>EKLSTHINNAERSGYLSIDVAAKKEAYDLSSAQKRFYVLHRLHPTSTAYNDTATVLLEG<br>ELDLKRLELSFKNVIQRHEVFRTSIQMIDDEPKQFIHDEVEFELMYVEAKETEAPQLVR<br>NFIRAFNLEIAPFLRIGVVRLHETKHILIVDIHHIITDGVSFDIFVRDLMKLYSGEELP<br>QLRIQYKDYAEWQNSEREQEVVKKQKEYWMNRFEDQIPVLNLPTDYSRPEMSNFVGDTL<br>SFEINQELTDKIRKLVSQEESTLYIVLLAIYNALLHKYTGQEDIVVGSPIAGRPHADLQ<br>NIMGVFVNMLPMRNQPKGERTFKTFLAEVKENSLKAFENQKYQYEDLVLSLGLQGNITR<br>NPLFDVMFVLQNMDTEKLEFNGLHVSPFEFGHQRAQVDLLLRAVEAQDKLEMTIEYSTE<br>LFRRGTMEKFVQRYLELLEQVASNVQIKLQDITVSHDFEELVLQIDQGDFNF	
antiSMASH	Laterocidine<br>(Non-ribosomal peptide synthetase NRPS)	MKALFEKEKAFWSNKFDAEDHLVSLPYNRTTTISRNTDSFHTVCTTLPPHISQRISSIA<br>GGSSLAIFMILLAGVECLLHKYSNEENVIVGVPGIRTSDDAPPLSNQLLILKNEVHSKS<br>TFKSLLNQMKTSVSEAIKHQSLPFWNFIDRLNVHYDSKHVPIVNTIVSLKEIHPLDFEE<br>HATFDIHFQFDLENGSLHLTTIYNENRYDQELIAQVGRHLSHILSIVLFNLDLSLDQVD<br>LFLEAEKEQLLLTFNNTKTAYPREKTIQQLFESQVQRTPDQVAIVFEDKQMTYKELNEK<br>ANQLARTLRTKGVQAGQLIGLMVERSLEMIIGIFGILKAGGAYVPIDPEYPNDRIRYML<br>EDSGAKLFVLQSHLQHRVDFKGTCLLIDDSEAYSEDGSNLEPLAEATDVAYVIYTSGST<br>GAPKGTQIRHFSATRVVMNTNYIEIGEKDTLLQLSNYVFDGSVFDIYGALLNGAKLVLI<br>KRTDMLDLNKLSVVIEQQNVTVSFMPTAFFNMLVDHQLTCLKNIRKVLIGGERASVSHV<br>RKAFDYLGPERLIHVYGPTESTVFTTFYPIDSPLEAVATVPIGKPLANTLVYVVDQANN<br>LKPIGVTGELCIAGEGLSIGYLNREELNVEKFVDNPFVPGTQMYRTGDLVKMLPDGTIE<br>FLDRIDKQVKIRGFRIELGEIENALLTFPEINEAVVMDGIKDGNKYLAAYYVVKESIQV<br>GELLRNLSEKLPEYMVPDYFMPLNQMPLTPNGKIDRKMLPDVEDTLQIEVEYVAPRTKL<br>EEQLEDIWKRILSIPKIGMRDNFFDRGGHSLRATALVSTIHKEIQKNIQLRDVFQNPTI<br>EQMAQLIEDMEQTAYSIIPIMEEKPYYPVSSAQKRLYILGQLVGGELSYNMPSALILEG<br>VLDRVRLEKAFDQLIARHEILRTSFELVNGEPIQRVHKEVSFAIEYVQVANEAVAEGFI<br>RDFIRMFDLQSAPLLRVGLVELASDRHILLFDMHHIISDGTSFSVLIQEFSGLYGKEDL<br>PEQRIQYKDYASWQQQQMQSEWYQQQENYWLQAFSGELPVLHMPTDYTRPAVRSFAGSL<br>LEFVIDKQRSEELKQLAAQTGSTLYMVLLAIYTTLLSKYSGQEDIIVGTPIAGRPHADL<br>ESLIGMFVNTLAIRNYPMSEKTFYEYLQEVKETSLKAYENQDYLFEELVDKLDVNRDMS<br>RNPLFDTMFVLQNLESKEQDLEGLRITPYASEHTISKFDLTLNAMEDADTIMCSFEYAT<br>TLYKRETIERMTEHFLQIIDVLVSNPHAKLSSINMMTPQEKALILETFNDTTVDDLQEQ<br>TIHQLFESQVERTPDQVAVVYKGEQLTYREVNERANQLARTLRAEGIGPDQLVGIMVER	*Bl* 1821L= 73

		SLDMMVGLLAILKAGGAYVPIDPAYPEERIRYMLEDAGAKLVVVHSHLQERISFAGKCL<br>TLDEEASYQKDASNPEPISGPADLAYVIYTSGSTGKPKGVMITHQAVVNLVHGISDRID<br>FAQGKSLLSVTTISFDIFVLETFLSLCKGVRVVLASGEQQTDPQALSEVILEQQIDMIQ<br>MTPSRMQMLLSSMHGTERVKCLKGIREIMLGGEALPASLLNTLREHTQARLYNMYGPTE<br>TTVWSSVGEVTTGESITIGKPLMNTLFYILSTEGHPQPIGVAGELCIAGEGLARGYWNR<br>PELTAEKFVENPVMAGSKMYQTGDLAKWLPDGTIEYLGRIDHQVKIRGYRIELGEVEAQ<br>LVNMEAVQETVVIAREDQAGHKQLCAYIVAEKQLTMSELKSRLSHELPGYMIPSYFVQL<br>DKIPHTPNGKIDRKALPAPEDGSHMGVEYMAPRTWMETKLVQIWQEVLGLPNIGMKDNF<br>FDIGGHSLRATNLVSKIYKELNQNLPLRDAFQYPTIEQMAEVLEKREEIAYASIPIIEE<br>REYYPVSSAQKRLYILSQLTGGEISYNMPDVVVIEGELDQGRLEIAFRKLISRHETLRT<br>SFELVNGEPMQRVHQDVDFVVEYAQASMEEAEAYIQGFVRTFDLEQAPLLRVGLVELAR<br>DRHLLMFDMHHIISDGVSLGILIQEFVSLYGGEELSPLRIQYKDYAAWQQEGVKSEEMK<br>QQEAYWREVFQGDIPVLDMPTDYVRPAMQSFEGDTIEIVISKKSRDGLRKIAAQTGSTL<br>YMVLLAAYTTLLHKYTGQEDVIVGTPIAGRSHPDLEPIIGMFVNTLSIRTYPVGEKTFY<br>EFVQEVKEKALGAYENQNYPFEELVDKLDVKRDLSRNPMFDTMFELKNLEQEELSIEGL<br>HLTSYVDDHAVSKFDLTWNAVEQAEGIVCSIEYATSLYKEETVERMGEHFVQLIETIVH<br>DPHATLSSLTIITHQEKEQILAVFNDTTSDYVADKTIHQMFESQVERTPDQVAVIYEAS<br>QLTYRELNERANQLARTLRKEGVQADQLVGIMVERSLEMMIAIFAILKAGGAYVPIDPE<br>FPEERISYMLEDAGTKLLLVQNHLRDRVSFAGKLVDLNDGQMYSKDKSNLLPINDSQHL<br>AYVIYTSGSTGKPKGVMVEHHSVINRLMWMQTRYPITETDTILQKTAITFDVSVWELFW<br>WSLVGSKVCLLSLGGEKNPERIVETIAEQGITTMHFVPAMLHAFLEYLEQCSSKDIKRK<br>LHNLRQVFASGEALAPSQVARFHHIIASVNQAQLINLYGPTEATVDVSYFDCRADQEYT<br>VVPIGKPIFNTQLYIVQNETEHLQPISVAGELCIAGVGLARGYLNRPELTAEKFVNNPF<br>VPNERMYKTGDLARWQPDGNIEYLGRIDHQVKIRGYRIELGEVEAQLLKVETIQEAVVI<br>AREDATGQKQLCVYFVADKPLIVKDLRSTLSQHLPGYMIPSYFVQLERMPLSPNGKIDR<br>RALPAPEGSMQTGVEHVAPRTQIEVQIAKIWQEVLGIPSIGVKDNFFDSGGHSLRATTL<br>VAMMHKEMGISLPLRDVFGYPTIEQMAERVSGIDYVAHSSIPVIQEREFYPVSSAQKRL<br>FILNQIEGGGLSYNMPSVLMVEGQLHRENLEEAFQSLIDRHETLRTGFEMVNGEPIQHI<br>YRDVTFEVEYSQAKEGETAEIIQGFVRSFDLAKAPLLRVGLIELEADRHVLMLDMHHII<br>SDAVSTSIFVQEFFQLYKGEELEPLRIQYKDYAVWQQEEVQSERMINQEAYWLEVHSGE<br>SPVLDLPTDYPRPAVQSYQGDTFEFVIDQQRSDGMRQIAAQTGSTLYMVLLATYTILLS<br>KNSRQEDIVVGTPIAGRPHADLGGLIGMFVNTLAIRTYPAAEKTFYEYVQEVKEHALHA<br>FENQEYPFEELVEKLHVARDFSRNPLFDTMFVLQNVEQGEHTIEGLQLRPYQGEHTVSK<br>FDVTLYAEEDDETIVCTFEFATSLFKRDTIERMAEHFLQIIDSIMIDSHTKLSTIEIIT<br>PQEKELILKTFNDTVADYPREKTIYQLFESQMERTPEHVAVVFEGEQLTYRELNERANQ<br>LARTLRAEGIEANQLVGIMVERSLDMMIGLLGILKAGGAYVPIDPDYPGERIRYMLEDA<br>GAKLVVLQSHLKEQISFTGRFVALDEEASYQKEISNLEPISGPADLAYVIYTSGSTGKP<br>KGVMILQQAVVNLLQGITNQIDFTQGKSLLSVTTISFDIFVLETLLSLSKGVRVVLASG<br>IQQIEPRALSDVIQKQQVDMIQMTPSRMQMLLSSGHIQCLQGLQEIMFGGEALPASMLK<br>TLRENTQARLYNMYGPTETTVYSSVGQLMEGENITIGKPLVNTTCYILNAENHIQPIGI<br>AGELCIAGEGLAKGYWNRPELSAEKFVENPFVAGERMYKTGDMARWLPDGSIEYLGRMD<br>HQVKIRGYRIELGEIEAQLLQVPSVHEAVVIAREDQVGQKHLCAYFVAKQQLTVGEIRS	

		SLLQDLPTYMVPSYFIQLEQLPLTPNGKIDRNALPAPEGNIHLGVEYTAPQTPTELQLV<br>EIWKEVLGVLKIGRKDNFFELGGHSLHVLELIRKIYTDIRVEIPIRVVFEMPTIEGMAQ<br>EIVRSMFEKNHNNPIMKLNEHGRVNVFCFPPAIGYGMAYIDMAKLLENHCVIYGIDFIE<br>EYKNDEDLMEQYVKILTDVQDKQPYVFLGYSMGGNLAFEVAKAMKLKGYEVKDIIMLDS<br>IRSNGEAEFSEEEAIKQIDSLLEDMPEQYKHLVTPAYTNRIYSYARYRNQLVNTGTVQA<br>NIHELIAKDSTRKGSTEDCSWSWGDATLGTHTEHQAKGTHEEMLDPEMIGENVRLVRLI<br>VQQIIDETYAVTSNATNRFR	
antiSMASH	Laterocidine<br><br>(Non-ribosomal peptide synthetase NRPS)	MKALFEKEKAFWSSKFDTDDHIVILPYNRTSTTSRNTDSYHSIYSTLPPQIFQRISSIA<br>GGSQLAIFMVLLAGIETLLSKYTSQETIIVGVPGIRTSNDAQPLSNQLLFLKNEVHSKN<br>TFKSLLNQMKSSISEAMKHQSLPFWNFIDNLNVHYDSNRVPIINTIVSLKEIHPLDFEE<br>HAAFDIHFQFDLENGSLQLNTIYNVNRYDHELITQVGRHLSHILSIVLFDLDLSLELVD<br>LLSEDEQEQLLLTFNNTTTTYPREKTIQQLFEEQVERTPDQVAIVLDDKQLTYKELNER<br>ANQLARTLRNKGVTTGQLVGLMVERSLEMIIGIFGILKAGGAYVPIDPEYPNDRIRYML<br>KDSGAKVFVLQSHLQQRADFEGTCILLDDSEAYSEDSSNLEPVAEATDVAYVIYTSGST<br>GTPKGTQIRHLSATRVVMKTNYIEISEEDTLLQLSNYVFDGSVFDIYGALLHGAKLILI<br>KKTDMLDLHKLSSVIEQQNVTVFYIPTAFFNTLIDNQLTCFQNIRKVLFGGERASVSHI<br>RKAFHYLGPERLIHVYGPTEGTVFTTFYPINSSIEEDVATVPIGKPLANTLVYIVDQFN<br>NLKPIGVPGELCIAGEGLSKGYLNREELTGEKFVDNPFVPGTQMYRTGDLVKMLPDANI<br>DFLDRIDKQVKIRGFRIELGEIENALLTFPEINEAIVVDGIKDGNKYLAAYYVAKEPIE<br>AKELLKNLSAKLPEYMVPDYCISLDQMPLTPNGKIDRKMLPAVEESLPIEEYVAPRTLL<br>EEKLEDIWKGILGLTQVGIKDNFFERGGHSLRATALVSTIQKELQKNIQLRDVFQHPTI<br>EQMAQIIENMEQTAYSIIPIMEEKPYYPVSSAQKRLYILGQLAGGEISYNMPSALILDG<br>VIDRERIEKAFQQLIARHEILRTSFELVNGEPIQRVHKEVPFTMEYVQVANEEAAEGII<br>RDFTRMFDLQSAPLLRVGLVELAQERHILLFDMHHIISDGTSMSVLIQEFARLYEGEVL<br>PKQRIQYKDYASWQQQQIKSEWYQQQENYWLQAFSGELPVIQLPTDYTRPAVRSFEGNL<br>LEFRIDKQTSEELKQLASQTGSTLYMVLLTIYTTLLGKYSGQEDIIVGTPIAGRPHADL<br>ESMIGMFVNTLAIRNYPRGEKTFYEYLQEVKETSLKAYENQDYLFEELVDKLDVNRDMS<br>RNPLFDTMFVLQNLESKEQELEGLRITPYASEHTISKFDLTLHAMEDADTILCSFEYAT<br>TLYKKETIERMAKHFLQLIDGLLSNPHAKLSTIEMMTMQEKTLILETFNNTIVEDVEEQ<br>TIHQLFETQVQRTPDQVAVVYDGEQLTYREVNERANQLARTLRAEGIRSDQLVGILVER<br>SLDMIIGLLGILKAGGAYVPIDPSYPEDRIRYMLEDAGATFVVVHSHLQDQIIFDGFNG<br>KCIVMEEKASYHKDTSNLEPCNSPSDLAYVIYTSGSTGKPKGVMITHQAVVNLIHGITK<br>RIDFAEGKSLLCVTTISFDIFVLETLLSLSKGVRVVLASENQQTDSQALRNVISEQQID<br>MIQMTPSRMQMLLSSVDGTDQVKCLQDVREIMLGGEALPISLVNALHEHTSACLYNMYG<br>PTETTVWSSIGEVAVGQPVTIGKPLMNTQFYILSTDGHPQPIGVAGELCIAGKGLARGY<br>WNRPELTTDKFVENPFIPGSKMYKTGDLAKWLPDGTIEYLGRIDHQVKVRGYRIELGEI<br>EEQLRNMEEIQEAVVIAREDHAGVQHLCAYIVVEKQLTISELKIKLAYQLPNYMVPSYF<br>IQLDKMPHTPNGKLNRKALPAPEDSSIHSGAEYVPARTWMESKLVQIWQEVLGLPKIGV<br>KENFFDIGGHSLRATHLVAKIYKELNQNFPLRDVFQNPTIEQMAEILKGREKIAFTSIP<br>IIDEREYYPVSSAQKRLYILSQLTGGEISYNMPDVIQIEGELDKERLEMVFRKLISRHE<br>TLRTNFELVNGEPMQRIHQNVEFSIEYTKANVAEVKGHIQNFVRTFDLEQAPLLRVGLI<br>EITKDRHIMMFDMHHIISDGVSLGILIQEFISLYKGEELSPLRIQYKDYAVWQREGVLS	*Bl* 1951= 67

		EEMKRQEAYWLEVFKGEIPVLDLPTDYIRPAMQSFEGDTIEFIIDHKSRNGLRQIAAQT<br>GSTLYMVLLAAYSTLLHKYTSQEDVIVGTPIAGRSHPDLEPIIGMFVNTLSIRTYPVGE<br>KSFYEFVQEVKDTALGAYENQNYPFEELVDKLDVNRDLSRNPLFDTMFELKNVEQEELS<br>IEGLQLSPYENNHAVSKFDLSWNAVEESERIVCLIEYATSLYKKETVERMGLHFVQLIE<br>AIVNDPHAPLSSLQIITNQEKEQILGLFNDTTTDYAEEGTIHQMFENQVERTPDQVAVI<br>YENSQLTYRELNERSNQLARTLRNEGVQAGHLVGIMAERSLEMLIGIFAILKAGGAYVP<br>IDPGFPEERISYMLKDANMKLLVLQNHLRDRIPFDGKRINLQDEQMYSEDKTNLLPVNS<br>SRDVAYVIYTSGSTGKPKGVMVEHHSVINRLMWMQNQYPIDETDIILQKTSITFDVSVW<br>ELFWWSMVGSKVCLLAVGGEKNPEQIVETIAKQGITTMHFVPAMLHAFLEYIEQCPRKD<br>VRNKLHCLRQVFASGEALTPSQVTKFNQIVATVNQAKLTNLYGPTEATVDVSYFDCHAD<br>QEHAIVPIGKPIFNTQLYIVQNETNNLQPIGVAGELCIAGVGLARGYLNRPELTAEKFV<br>MNPFTAGKTMYKTGDMARWLPDGTIEYLGRIDHQVKIRGYRIELGEIEALLLKIASVDE<br>AVVTAREDHTGQKHLCAYFVAKQQLTVSELRSSLSQDLPTYMIPSYFIQLEQFPLTSNG<br>KIDRKVLPSPEGKLHLGVEYEAPQTPMEVQLVEIWQEILGVSKIGRKDNFFELGGHSLT<br>VLELVRKIYMNIRVEIPIRVVFEKPTIEGMAPEILRPIFEKNNRNPIMKLNEHGHVNVF<br>CFPPAIGHGMAYIELAKLLENHCVTYGIDFIDEYTNDEDLMEQYVKIVTDVQDKQPYVF<br>LGYSLGGNLAFEVAKAMELKGYMVSDIIMLDSIKRNGASEFSEDEAVKQVDALLEDMPE<br>QYKHFVTPTFTHKIYSYAKYRDQLVNTGSVQANIHELIAKDSTKLNSEEGNCLSWRQAT<br>RSNYSEHQAKGIHEEMLDPEMIEENAKLVRHIVQKIIDETYAVSAHSTDRLR	
BAGEL4	Laterosporulin (Class II bacteriocin)	MKGMGCQYECPDAVKNWAYQDYQCHPVEKKYYRHVYAVCMNGLNVYCKTEWSTKC	*Bl* 1821L= 61.5<br>*Bl* 1951= 61.5
		MLVTDDVLTFPKLAVYREKEDYIIHNYLVSSWIVLNQQEYEVAHEMIYNKKSPQGLIDE<br>QYDVSLVKRVIGLIRLYKIDSFNVDTENKQSTHKPVPKSVYFVTTYKCNLNCVYCYAES<br>SPSRSMDNDISTEEAKRVITEVKELGTKTIVFTGGEAFLRKDLVELMEYSNFLGLNVHM<br>ISNGSFISNKEIANKIAEITKLVTISLDSMIEDEHDKNRGKGSWQKAKKAIDLLLEAGC<br>KLKINQTITKNNMDAVEDVIDYTNKNNIRLIIAPVASLGRGKTHEHELNYVQRIKIENT<br>IINKQPNFDAVNQFFIKNHCGHAYSEFSVDSKGDVFPCKIMHNPEFLGGNVKDKSLEEI<br>YYESPVFTQSRNRITDNLPICGKCTFKHMCGGGCRAIQWSDTDSIDGTNTEECKIIKSN<br>LKKHMWNYFRKSKEEVPS	*Bl* 1821L= 27.1<br>*Bl* 1951= 27.1
BAGEL4	Sactipeptides	MNYDQNPFIVIWEVTRACALRCLHCRAIAQPNRHPDELSTEEGKEVLNQIKAMGNPLLV<br>FTGGDPLMREDLYELIDYAVKIGLRVSMSPSATPRVTEKAIVKAKKAGLSRWAFSLDGP<br>TADVHDYFRGTPGSYDITVKSLATLRKHGLPVQLNTTISVYNAHLLEEMSQMVEEWDAV<br>LWSVFFLIPTGRGQASDAVSPMEQERIYRWLIEKSQIASYSIKTTAAPAYRRALYFATQ<br>KSMQTQPVQVTRMDNLGRSAGGVTDGKGFVFISHVGDVHPSGFLPIDCGNVRDTPLADI<br>YRNHTVFQALRNPDGYSGKCGVCNFNQVCGGSRARAYAMTQDYLASDPSCMYIPPKWVE<br>GYEQNQTGYFQ	*Bl* 1821L= 28.9<br>*Bl* 1951= 28.9
		MPESLYKGLEEDMMIGLSSLLETTTTFGDKLRYSEASRNQKTGTHQGSGPVVAWNITKT<br>CNLRCLHCYSSSENKKYEGELSTEEAHDLINQLADMKVPVILFSGGEPLIRPDIFELAE<br>HAISRGIRVTFSTNGTLIDEKKAQRLKDLGISYVGISLDGMQETHDFFRKKEGAFDLAI<br>RGIRNCMAVGQKVGLRFTMNKHNIDSLPDIFKLIEEENIPRVCFYHLVYAGRGGVEDDI<br>THQQTREALDFIIEKIHYFQESGQPREILTVDNHADAIYLYLKVMETNPELAERIWEKL	*Bl* 1821L= 30.4<br>*Bl* 1951= 30.4

		QRNGGNRSGIAFCNIDHRGIVHPDQFSQDIALGNLREQSFAEIWQGEHPILLGLRDRKT<br>KIHGRCATCDYFSVCNGNFRPRAQGFYGDFWASDPQCYLTEDEIHFATSV	
BAGEL4	UviB	MGSYGSLFYLPQGGEEERGSMEEMIYKAVVSQGPFVALFVWLLFSTKKEGRDRETRLVK<br>QAQTREEKLMEHNERMVIHLERNTATLQQIERSLNGLENDIHELKEKVG	*Bl* 1821L= 32.2<br>*Bl* 1951= 32.2
BAGEL4	Lanthipeptide<br>(Class I bacteriocin)	MAKQLEKELTTQYGKTNKEENLFTAADFFMLRLPLFPLENFYELNKMDDYLTAIREYAR<br>EEKVREAILVSSPSLYAALPNLESDLTSRKTKQTLSSFIRYFLRMSTRATPYGLFAGVA<br>LGSLTDKTDILLGNCDYNQKRTRPDMEWLLAIIKGLEQRLYVVKQLKVQKNHASLQVGG<br>RIELTLATEYGQIKRLDGMQKERISIRATEVVHNVFALTEHPILFEELIARLMELYPKA<br>GEQKVTGLLWQLFSQEFLISELRPPLTVANPFDYVMGHLKLLDGVQEIYGELVYIQEMM<br>KRYDELPIGNGIQAYQELIARMKAIEASDTPIQVDFHLASNKVCLHNGIGEEVAKAAEC<br>LWRLSFVQRGATHLRSYHKDFLERYGTSREVPVLELLSEEIGLGAPAGYMSPPSNRREI<br>VSPLKYSDQREAIVMRKVVQAIATKKREVEITNDLLEQLDADPVNPKYAPDSLEMYVEV<br>IASSQEAIDKGEYLLEVGPMPGSSKVGQSFGRFLDILGTEAIEKHEQVHKQVQGQFEDV<br>IMVDAVYLPAAGRMANIMLHPSLSEYELAIGTNCSEKAKHPLLLEDIVVCATVEHLYLK<br>SRTHGKKIIISSDNMLNFRNSPNVYRFLREVSFENIRNWQPFSWGNMEGSPYLPRVRYG<br>RTILSPAVWKLTPSSLGMKENPKEDQLWYDAFSKWRMEWDVPRHVYMAFGDNRILLDLD<br>NSYHIEEIRSELFKSKGIKFREKIGGLEKNWVNGPDGHFAMECVIPLIKQKHLVKMNPP<br>VYRKNMVLSKEQYIKFPGSDWLFIKLYGGQQRQNEFIVEKIRTFADSMVQKGAADEWFF<br>MRYLDPEHHIRLRFHGKPEKLIQEILPELYRWIQECQREGFIQRMVIDTYDREVERYGG<br>PTMMANAERVFSKDSQTTVKLLGLLRYKQTNLPDYVLATISIIDIMTRYGLSFEQQFEW<br>MEQVVKKDAHREEFRKWRKTLLTLADPRNDWQGLRSHPSGEEIREAFYLRTDALNIFWK<br>LVSEGEQNKSLWSSKATILGSLIHLHCNRIFGVNREMEEKAMAFVRHTLHSQLHWRENV<br>GVSI	*Bl* 1821L= 42<br>*Bl* 1951= 42
		MEKNKVVTGNEHVWIGIKKEELRTKAFSIALKVAEKLRDPIQVEKVVMHKDNISTIGEV<br>PQYPWGATSLSHGYPGTILFFAELDRHFPEEGWDIVAHEHLLALQEKIRSIGVSSDSMF<br>GGIAGISFATLVASHNRKRYQRFLGQLDQLLAQRTVAMIEDEYVLHKKRGGSNPHIYDV<br>INGVSGIGRYLLASKQINELHSALQDVLRYLVYLSQPMEWEDGRKIPGWYLTQEMQYLD<br>KDKEMYSKGNFNLGFAHGIPGPLALLSLATREGIEVTGQRDAIHRIAEWLMRWQETDEY<br>GIMWRDRVSLEDELESEFRSQSSFRRDAWCYGTPGVAHSLHLAGEALQDSSYCNLAFQA<br>YDHIFKRPVSDWNLYSDTFCHGKAGLLQMTMRMDMVADDERYQPYIEYLTETLVNNYNP<br>SLPFGYQDIEPRDGQRIGLNKAGALEGAAGIGLALLSASSKQEPIWDQPFLLA	*Bl* 1821L= 41.1<br>*Bl* 1951= 41.1
		MHDPFLKDKKLLVGLSGSSAVLGLPAYLGVFRAIFKEVKVIMTEAATKLIAPSTILLFC<br>DEVFIDEDLGLEKKMNHVELARWADLFIILPATANVIGQAANGIALNLLTSTILASPNP<br>VMFFPNMNRLMWTKKVVQRNVSQLREDNHIVVTPLEAMAYEIASGTMQPNYILPPGQTV<br>IQEMKNTLLEREEETVVST	*Bl* 1821L= 47.9<br>*Bl* 1951= 47.9
BAGEL4	Linear azole containing peptides (LAPs)	MGFVNKLVQPIGGLLQNVVPIPSLPGEPSYHVYSGIMGNLSHVIPTIAFSIDGAGNSTL<br>EEEAKRKAIAETVERYSSCMYNEKQMIWATGRELGEEALDLDMIPRCSDKELQHPRCPL<br>IKPDKDLPLRWVRGISLTHKRSVWLPAIMVYLNFEIMSKGEKIWLPISTGCAAHTTLEQ<br>ALLGAICEVIERDAIAITWLQQLELPKISVDSYPQWVQPYLENLDRNKHIQQIFYDATS<br>DLEIPTLYSIQLAPHNPQLTTMVMCSTELNPYHSFTKLMKESASSRIALQNGLKHPENL	*Bl* 1821L= 24.3<br>*Bl* 1951= 24.3

		DDFIKVYDGATYMGAPERASAYHFLLQTSKNKKISEMKNIETGNPKKDLVELVKRLEEK<br>GHEVFALDLTTDEARRANLHIVRVIIPTLMPLSFSYRARFLGTPRLYEAPRLNGYPIRM<br>EEEINIWPQPFA	
		VSSVVLVVGEGLLADLVCKELSAPYEVVRQTNLKAQIPKTAEIALVLHDAWNPFVHREA<br>EAVLQTSGIPWLRGFVSFGEGVVGPLVRPGTPGCSQCADLRYLMAGRERQEIWELQQRL<br>GEQANISRDAWASHTGLLQMTHVLVAEVQRVLTGEQAHLEGRLFLINLKMLRSSRHFIL<br>PDPSCEVCGQMPEDSAIAARISLKPNPKISADSYRSRPMDDLKKVLGKEYLDYRTGFLN<br>KKLRDLVSPFADVSVSLPLFTGDEGTAGRTHSYEDSEMTAILEGLERYCGIAPRGKRTV<br>IRDSFHNLADQALDPVKVGLHTEEQHERPDFPFVRFDPDRPMYWVWGYSFLQERSILVP<br>EMLAYYGMGDKDCFVFETSNGCALGGSLEEAIFYAILEVVERDSFLMTWYGELPLPRID<br>PFSIHDQELHLMLYRLQAVAGYDVYVFNATMENGIPSVWTLAKNRKEKGVNLICAGGAH<br>PDPVRALKSSIHELAAMLLTLNEKFEANREEYVRMFHDPFLVKKMEHHSMLYALTEAEE<br>RLQFLLNDHRPLRTFEEEFIPRPKYGDLTDDLKSLLQTFRRLNLDVIVVDQTTPEILRN<br>GLHCVKVLIPEMLPMTFGHHLTRVKGLDRVLQVPMELGYTKQPLTYEQLNPHPHPFP	*Bl* 1821L= 24.3<br>*Bl* 1951= 24.3

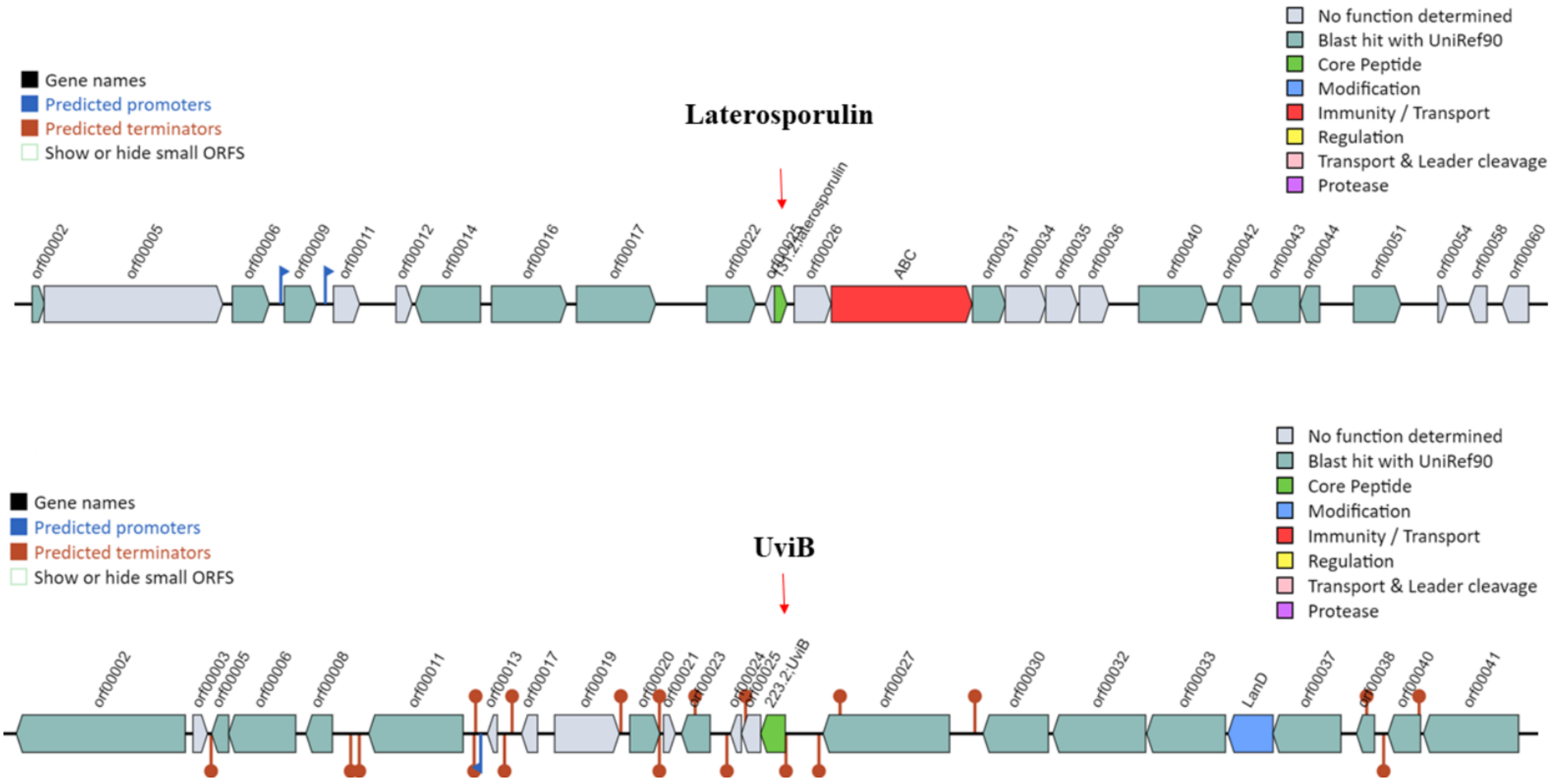

Fig. S6 BAGEL4 analysis of *Bl* 1821L (NZ_CP033464.1) genome showing the areas of interest encoding the core peptides (bacteriocins) laterosporulin and UviB (shown with red arrow)

Note: % amino acid identity of the predicted antimicrobial molecules (laterosporulin, UviB) of *Bl* 1821L with the known similar protein of *Bl* GI-9 and *Bacillus thuringiensis* serovor *israelensis* is presented in Table S1.

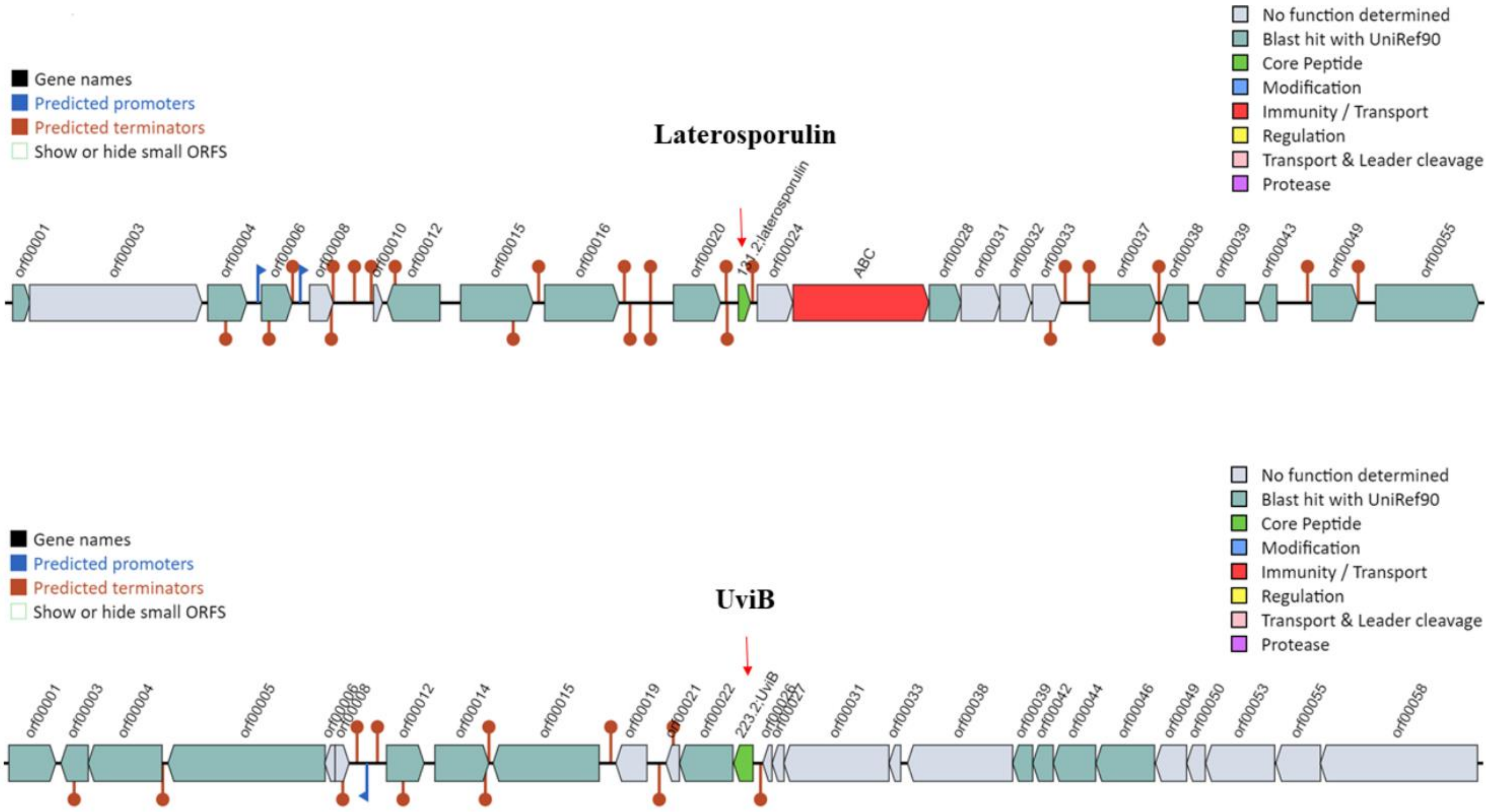

Fig. S7 BAGEL4 analysis of *Bl* 1951 genome (RHPK01000003.1, contig 1) showing the areas of interest encoding the core peptides (bacteriocins) laterosporulin and UviB (shown with red arrow)

Note: % amino acid identity of the predicted antimicrobial molecules (laterosporulin, UviB) of *Bl* 1951 with the known similar protein of *Bl* GI-9 and *Bacillus thuringiensis* serovor *israelensis* is presented in Table S1.

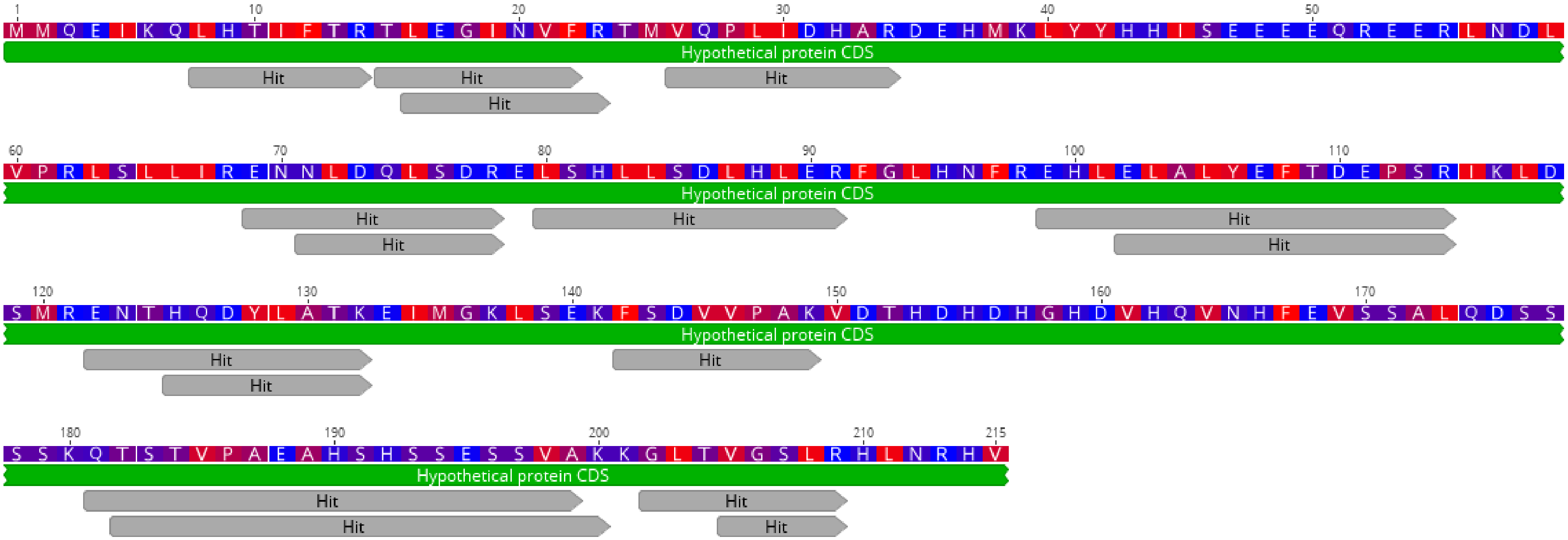

Fig. S8 N-terminal sequenced short amino acid match hits to the gene of 25 kDa putative hypothetical protein encoded in *Bl* 1821L and *Bl* 1951 genomes

Note: Refer to supplementary material for N-terminal sequence data of purified ~30 kDa excised bands of *Bl* 1821L and *Bl* 1951
https://doi.org/10.25400/lincolnuninz.16713442

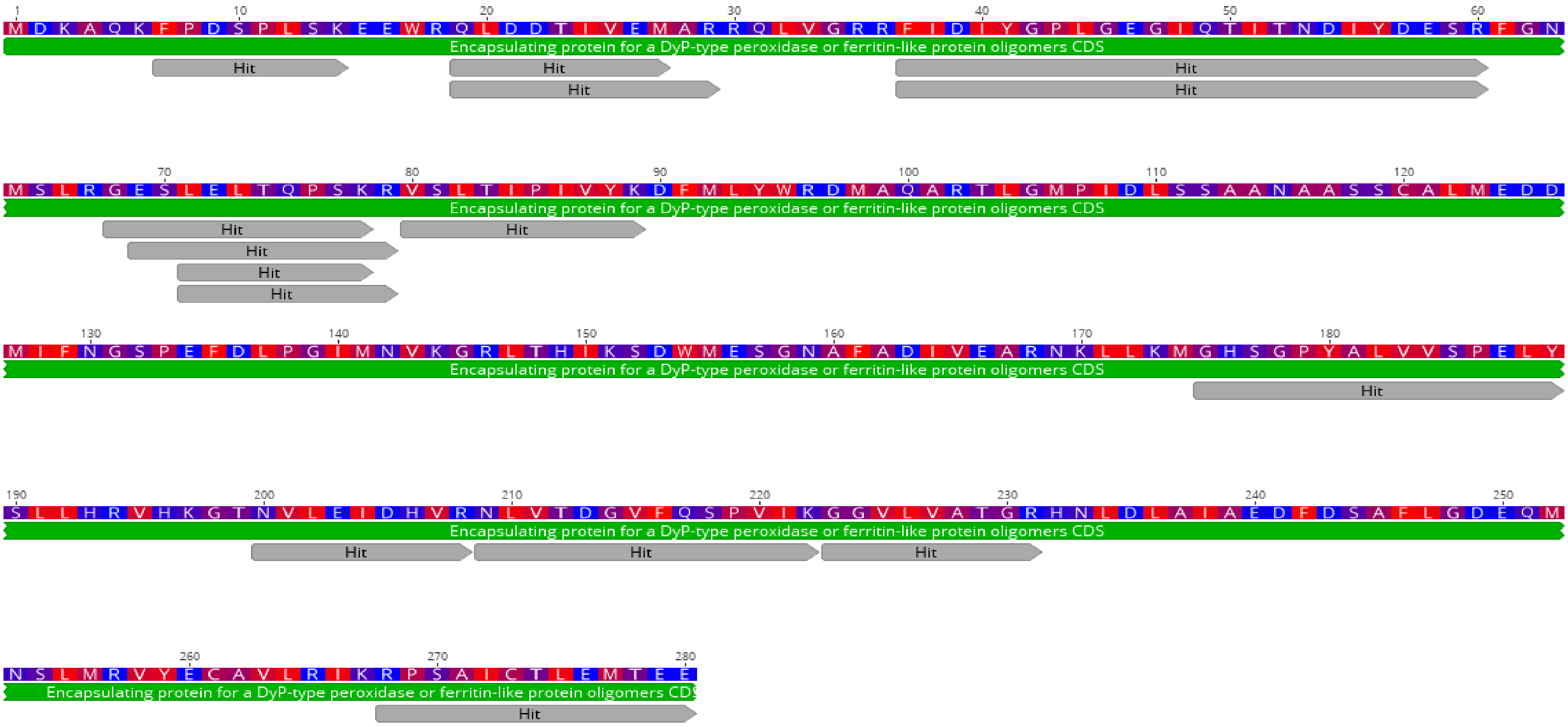

Fig. S9 N-terminal sequenced short amino acid match hits to the ~30 kDa putative bacteriocin family protein/encapsulating protein for a DyP-type peroxidase or ferritin-like protein oligomer gene encoded in *Bl* 1821L and *Bl* 1951 genomes

Note: Refer to supplementary material for N-terminal sequence data of purified ~30 kDa excised bands of *Bl* 1821L and *Bl* 1951

https://doi.org/10.25400/lincolnuninz.16713442

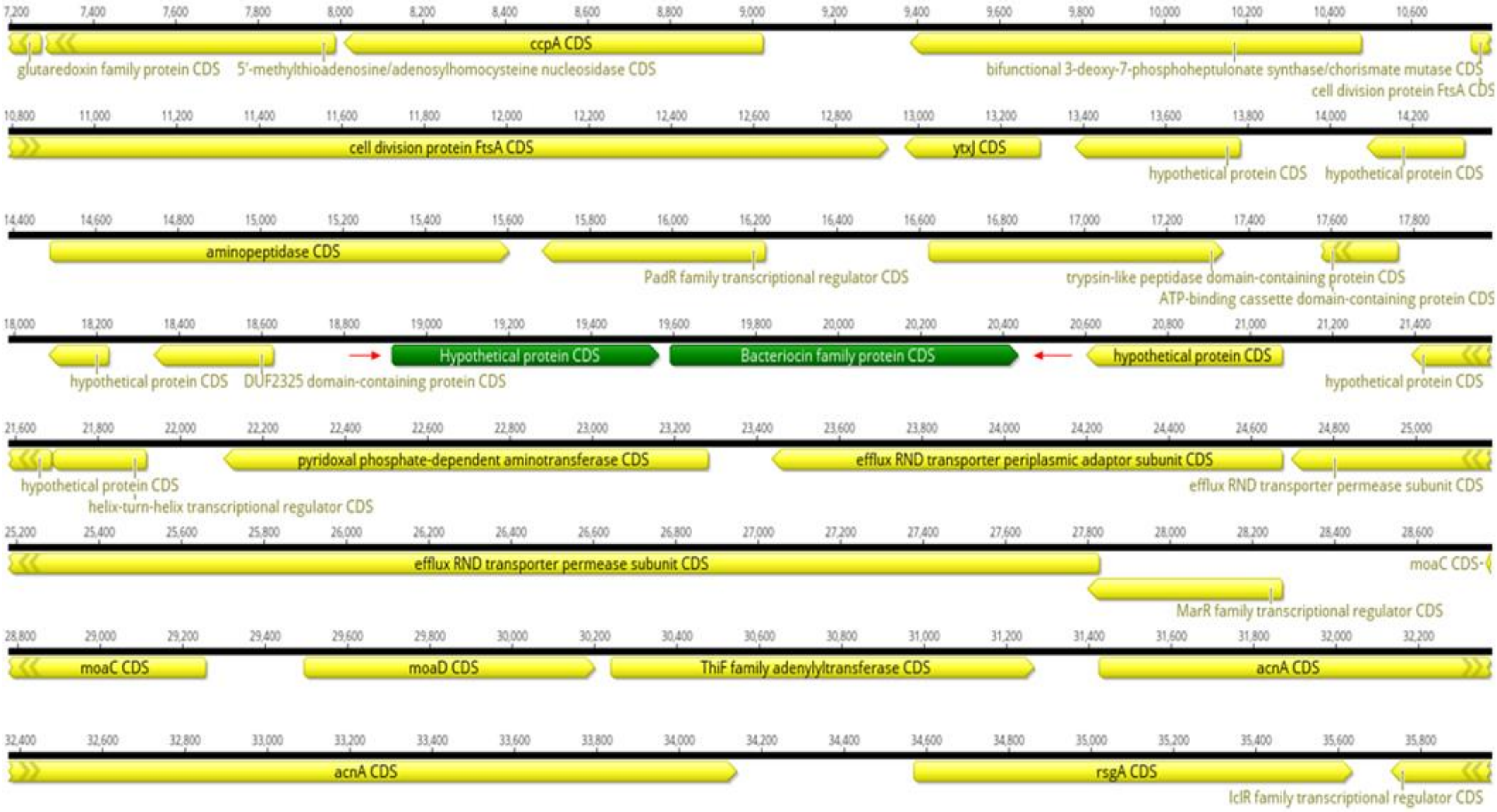

Fig. S10 Geneious output of the ~30 kDa N-terminal sequence of *Bl* 1821L identifying genes corresponding to a hypothetical protein (25 kDa) and a bacteriocin family protein (31.4 kDa) in *Bl* 1821L genome (shown in green with red arrow)

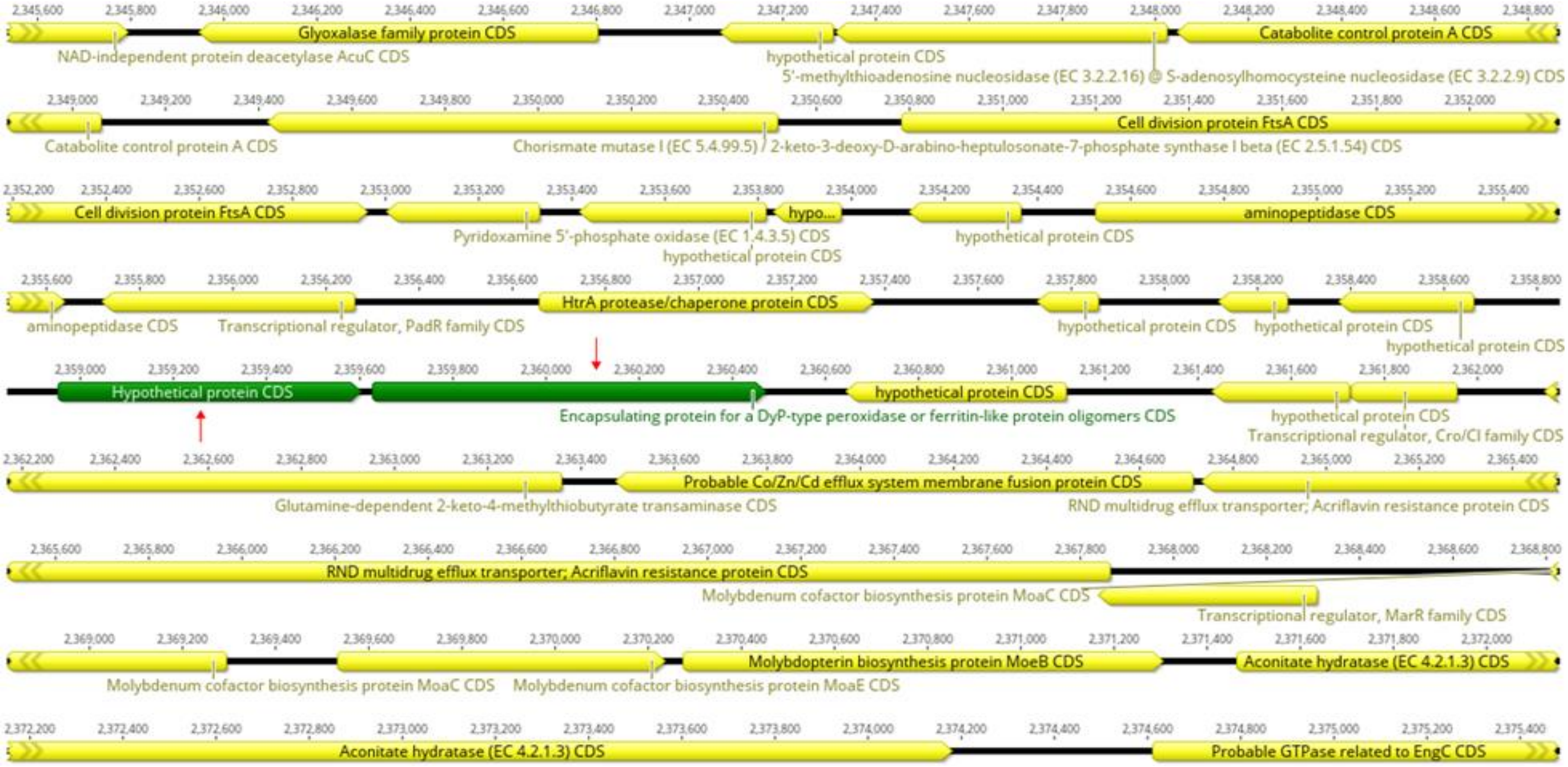

Fig. S11 Geneious output of the ~30 kDa N-terminal sequence of *Bl* 1951 identifying genes corresponding to a hypothetical protein (25 kDa) and a putative encapsulating protein (31.4 kDa) in *Bl* 1951 genome (shown in green with red arrow)

Table. S2 BLASTp comparisons of 31.4 kDa identified putative encapsulating protein of *Bl* 1821L and *Bl* 1951

GenBank accession	Protein	Host bacterium	Accession length (Amino acids)	% identity with the identified protein (GenBank accession WP_113757162.1)
QOS99091.1	Bacteriocin family protein	*Brevibacterium* sp. JNUCC-42	280	98.6
WP_003334816.1	Family 1 encapsulin nanocompartment shell protein	Multispecies (*Brevibacillus*)	280	97.1
WP_094700754.1	Family 1 encapsulin nanocompartment shell protein	Multispecies (*Brevibacillus*)	280	97.1
WP_212932429.1	Family 1 encapsulin nanocompartment shell protein	*Brevibacillus halotolerans* J5TS2	280	97.1
WP_197247050.1	Family 1 encapsulin nanocompartment shell protein	*Brevibacillus laterosporus* VKPM-B-13242/VKPM-B-13243/G25-129	280	96.8
WP_199793498.1	Family 1 encapsulin nanocompartment shell protein	*Brevibacillus laterosporus* G25-131	280	96.8
WP_018672702.1	Family 1 encapsulin nanocompartment shell protein	*Brevibacillus laterosporus* DSM 25/G25-130/NRS590	280	96.8
WP_258420007.1	Family 1 encapsulin nanocompartment shell protein	*Brevibacillus laterosporus* VKPM-B-10531	280	96.8
WP_104033635.1	Family 1 encapsulin nanocompartment shell protein	*Brevibacillus laterosporus* BGSP11	280	96.8

Table. S3 BLASTp comparisons of 25 kDa identified putative hypothetical protein of *Bl* 1821L and *Bl* 1951

GenBank accession	Protein	Host bacterium	Accession length (Amino acids)	% identity with identified protein (GenBank accession WP_113757161.1)
QOS99092.1	Hypothetical protein	*Brevibacterium* sp. JNUCC-42	215	98.6
WP_064017301.1	Hypothetical protein	*Brevibacillus* sp. SKDU10	215	91.2
WP_003334817.1	Hypothetical protein	Multispecies (*Brevibacillus*)	215	90.7
WP_101669076.1	Hypothetical protein	*Brevibacillus laterosporus* ZQ2	215	90.7
WP_031413111.1	Hypothetical protein	Multispecies (*Brevibacillus*)	215	90.7
WP_096887610.1	Hypothetical protein	*Brevibacillus laterosporus* Lak 1210	215	90.2
WP_121472860.1	Hypothetical protein	*Brevibacillus laterosporus* Bl-zj	215	90.2
WP_022585990.1	Hypothetical protein	*Brevibacillus laterosporus* PE36	215	90.2
WP_116334703.1	Hypothetical protein	*Brevibacillus* sp. VP	215	89.8
WP_119734623.1	Hypothetical protein	Brevibacillus laterosporus E7593-50/ K75/SAM19	215	89.8
WP_018672703.1	Hypothetical protein	*Brevibacillus laterosporus* DSM 25/ACRRF/G25-128/G25-130/ OSY-11/BGSP9	215	89.3
WP_197247052.1	Hypothetical protein	*Brevibacillus laterosporus* VKPM-B-13247/VKMP-B-13242	215	88.8
WP_199793499.1	Hypothetical protein	*Brevibacillus laterosporus* G25-131	215	88.8

WP_104067681.1	Hypothetical protein	*Brevibacillus laterosporus* BGSP7	215	88.8
WP_104033636.1	Hypothetical protein	*Brevibacillus laterosporus* BGSP11	215	88.4

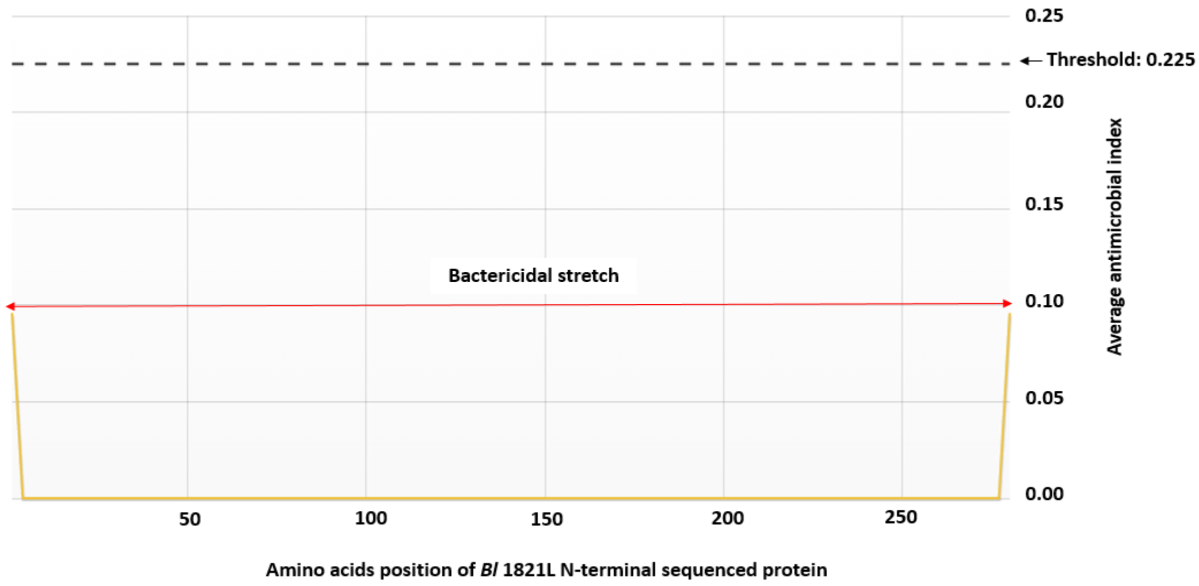

Fig. S12 AMPA analysis of 31.4 kDa putative encapsulating protein of *Bl* 1821L spanning -2 to 279 amino acid residues of the protein

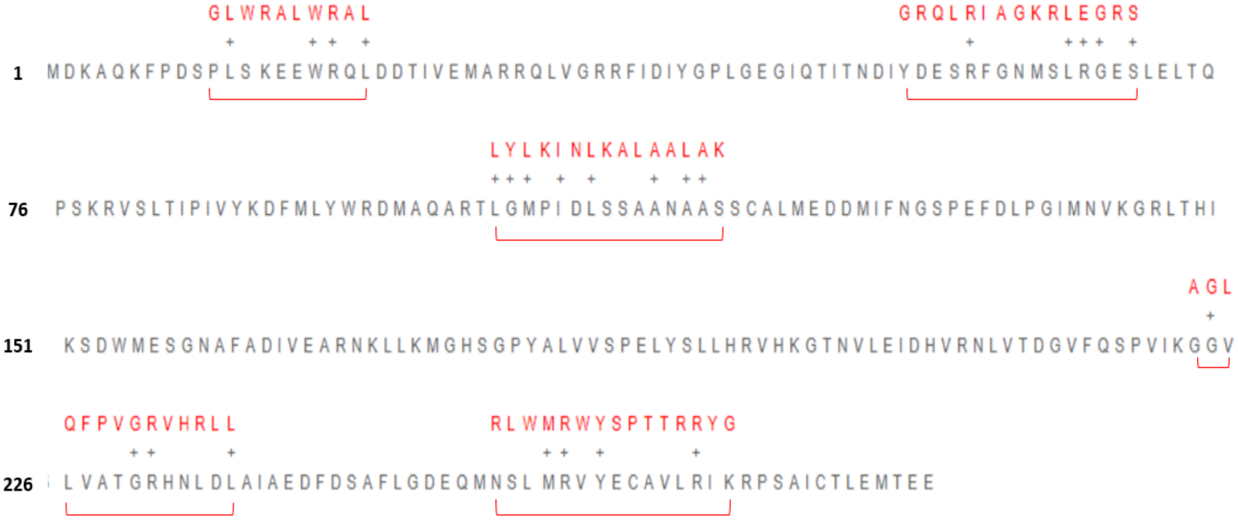

Fig. S13 Cell penetrating peptides (CPPs) motifs identified (shown in red) in putative encapsulating protein (31.4 kDa) of *Bl* 1821L

Table. S4 Cell penetrating peptides (CPPs) identified in *Bl* 1821L putative encapsulating protein (31.4 kDa) using CellPPD

Peptide sequence	Mutation position	SVM score	Hydrophobicity	Hydropathicity	Hydrophilicity	Charge	Molecular wt.
MARRQLVGRR	No mutation	0.36	-0.60	-1.02	0.71	4.00	1242.65
ARRQLVGRRF	No mutation	0.22	-0.56	-0.93	0.59	4.00	1258.63
RRQLVGRRFI	No mutation	0.10	-0.52	-0.66	0.46	4.00	1300.72
KGRLTHIKSD	No mutation	0.01	-0.41	-1.26	0.78	2.50	1154.48
ARNKLLKMGH	No mutation	0.14	-0.33	-0.81	0.33	3.50	1167.59
ECAVLRIKRP	No mutation	0.06	-0.32	-0.12	0.54	2.00	1184.60
CAVLRIKRPS	No mutation	0.11	-0.29	0.15	0.27	3.00	1142.56
AVLRIKRPSA	No mutation	0.05	-0.27	0.08	0.32	3.00	1110.50

Table. S5 Effect of crude *Bl* 1951 putative antibacterial proteins (ABPs) on the number of viable cells of *Bl* 1951 and *Bl* 1821L after incubation at 30°C for various time intervals. Data presents the mean values of four experiments. Values of % decrease/increase in the number of viable cells are calculated from CFUs values of corresponding time intervals

Time intervals (Hours)	*Bl* 1951	*Bl* 1951 + *Bl* 1951 crude ABPs	% Decrease/increase in no. of viable cells	*Bl* 1821l	*Bl* 1821L + *Bl* 1951 crude ABPs	% Decrease/increase in no. of viable cells	*LSD (5%)
1	8.54E+06 (6.931)**	1.00E+07 (7.001)	-17.28	2.08E+07 (7.318)	1.92E+07 (7.284)	7.51	0.853
3	4.81E+06 (6.682)	4.03E+06 (6.605)	16.36	6.58E+06 (6.818)	9.89E+06 (6.995)	-50.38	0.339
6	3.29E+06 (6.517)	2.45E+06 (6.389)	25.48	1.15E+07 (7.062)	1.03E+07 (7.013)	10.63	0.606
12	9.33E+06 (6.970)	4.81E+06 (6.682)	48.39	1.08E+07 (7.033)	9.23E+06 (6.965)	14.48	0.550
18	8.01E+06 (6.904)	9.60E+06 (6.982)	-19.81	7.03E+06 (6.847)	8.68E+06 (6.938)	-23.49	0.359
24	7.56E+06 (6.879)	5.99E+06 (6.777)	20.83	1.08E+07 (7.033)	1.27E+07 (7.1032)	-17.27	0.311

*=Least significant difference

**=The values in parenthesis indicate the converted value of number of viable cells (CFU/mL) into log_{10} CFU/mL.

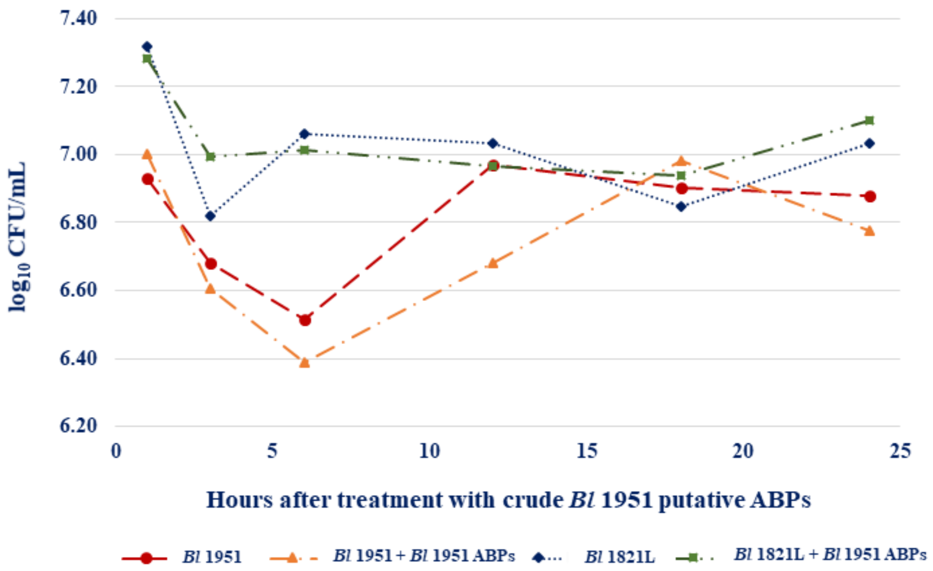

Fig. S14 Number of viable cells (log_{10} CFU/mL) of *Bl* 1951 and *Bl* 1821L with/without treatment of crude *Bl* 1951 putative antibacterial proteins (ABPs) after incubation at 30°C for various time intervals

Table. S6 Effect of crude *Bl* 1951 putative antibacterial proteins (ABPs) on the OD_{600nm} reading of *Bl* 1951 and *Bl* 1821L after incubation at 30°C for various time intervals. Data presents the mean values of four experiments

**Time intervals (Hours)**	***Bl* 1951**	***Bl* 1951 + *Bl* 1951 crude ABPs**	**% Decrease/increase in OD_{600nm} reading**	***Bl* 1821l**	***Bl* 1821L + *Bl* 1951 crude ABPs**	**% Decrease/increase in OD_{600nm} reading**	***LSD (5%)**
1	1.81	1.79	0.90	2.83	2.68	5.35	0.817
3	1.59	1.66	-4.31	1.82	1.76	3.16	0.286
6	1.44	1.54	-7.03	1.69	1.59	5.70	0.225
12	1.46	1.38	5.31	1.40	1.36	2.78	0.380
18	1.07	1.19	-11.74	1.28	1.23	3.81	0.296
24	0.98	1.12	-14.47	1.12	1.11	1.01	0.141
**% Decrease from the start (1 hour) to the end (24 hours) of incubation**	45.86%	37.43%		39.58%	41.42%		

*=Least significant difference

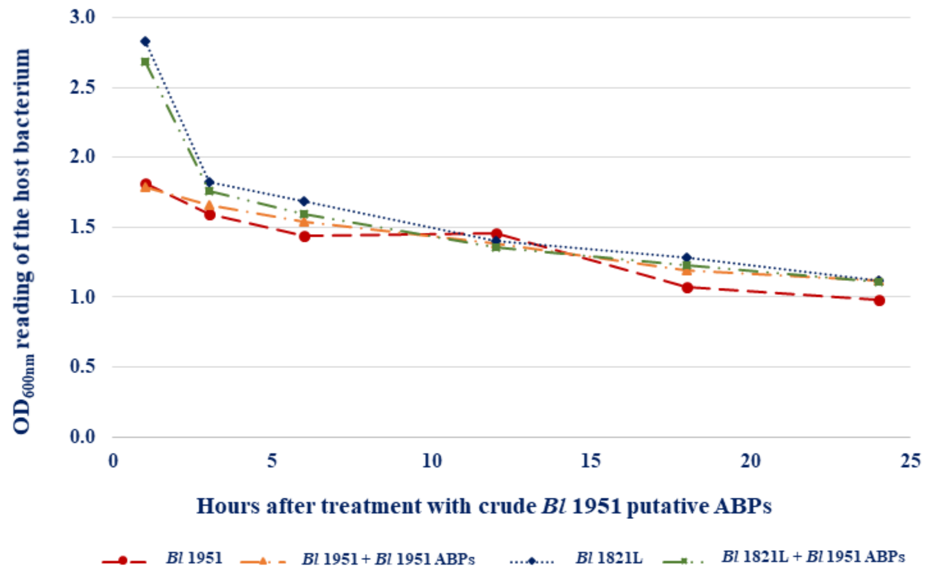

Fig. S15 Effect of crude *Bl* 1951 putative antibacterial proteins (ABPs) on the OD_{600nm} reading of *Bl* 1951 and *Bl* 1821L after incubation at 30°C for various time intervals

Table. S7 Effect of purified *Bl* 1821L ~30 kDa putative encapsulating protein (EP) on the number of viable cells of *Bl* 1821L and *Bl* 1951 after incubation at 30°C for various time intervals. Data presents the mean values of one experiment. Values of % decrease/increase in the number of viable cells are calculated from CFUs values of corresponding time intervals

**Time intervals (Hours)**	***Bl* 1821L**	***Bl* 1821L + *Bl* 1821L EP**	**% Decrease/increase in no. of viable cells**	***Bl* 1951**	***Bl* 1951 + *Bl* 1821L EP**	**% Decrease/increase in no. of viable cells**
1	5.30E+06 (6.724)*	5.65E+06 (6.752)	-6.60	6.65E+06 (6.823)	8.40E+06 (6.924)	-26.32
3	2.35E+06 (6.371)	2.85E+06 (6.455)	-21.28	6.35E+06 (6.803)	6.75E+06 (6.829)	-6.30
6	7.05E+06 (6.848)	3.60E+06 (6.556)	48.94	8.00E+06 (6.903)	4.50E+06 (6.653)	43.75
12	2.14E+07 (7.330)	2.79E+07 (7.445)	-30.14	2.08E+07 (7.318)	2.14E+07 (7.330)	-2.88
18	1.12E+07 (7.049)	1.67E+07 (7.221)	-48.66	1.21E+07 (7.081)	1.67E+07 (7.221)	-38.17
24	1.28E+07 (7.109)	1.82E+07 (7.259)	-42.35	1.24E+07 (7.093)	1.51E+07 (7.179)	-21.77

*=The values in parenthesis indicate the converted value of number of viable cells (CFU/mL) into log_{10} CFU/mL.

Table. S8 Effect of purified *Bl* 1821L ~30 kDa putative encapsulating protein (EP) on the OD_{600nm} reading of *Bl* 1821L and *Bl* 1951 after incubation at 30°C for various time intervals. Data presents the mean values of one experiment

**Time intervals (Hours)**	***Bl* 1821L**	***Bl* 1821L + *Bl* 1821L EP**	**% Decrease/increase in OD_{600nm} reading**	***Bl* 1951**	***Bl* 1951 + *Bl* 1821L EP**	**% Decrease/increase in OD_{600nm} reading**
1	3.02	2.90	3.97	2.90	2.93	-1.03
3	2.76	2.43	11.96	1.91	1.95	-1.83
6	1.93	1.93	0.00	1.93	1.92	0.52
12	1.69	1.73	-2.07	1.88	1.93	-2.39
18	1.53	1.58	-3.27	1.41	1.33	5.67
24	1.41	1.33	5.67	1.38	1.35	2.17
**% Decrease from the start (1 hour) to the end (24 hours) of incubation**	49.7%	46.9%		52.4%	53.9%	

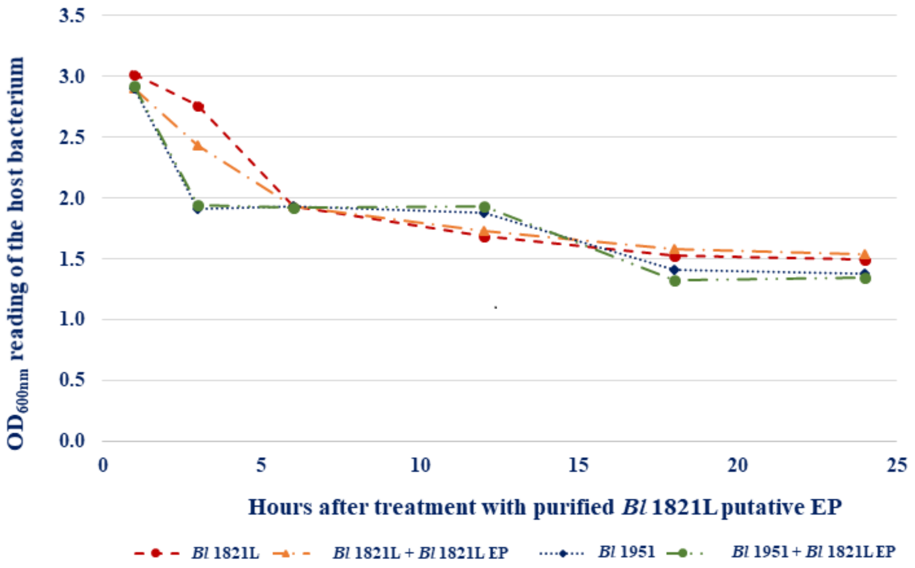

Fig. S16 Effect of *Bl* 1821L ~30 kDa purified putative encapsulating protein (EP) on the OD_{600nm} reading of *Bl* 1821L and *Bl* 1951 after incubation at 30°C for various time intervals

Table. S9 Effect of purified *Bl* 1951 ~30 kDa putative encapsulating protein (EP) on the number of viable cells of *Bl* 1951 and *Bl* 1821L after incubation at 30°C for various time intervals. Data presents the mean values of one experiment. Values of % decrease/increase in the number of viable cells are calculated from CFUs values of corresponding time intervals

**Time intervals (Hours)**	***Bl* 1951**	***Bl* 1951 + *Bl* 1951 EP**	**% Decrease/increase in no. of viable cells**	***Bl* 1821l**	***Bl* 1821L + *Bl* 1951 EP**	**% Decrease/increase in no. of viable cells**
1	3.05E+06 (6.484)*	4.10E+06 (6.613)	-34.43	5.30E+06 (6.724)	5.65E+06 (6.752)	-6.60
3	3.50E+06 (6.544)	2.55E+06 (6.407)	27.14	4.80E+06 (6.681)	3.35E+06 (6.525)	30.21
6	5.40E+06 (6.732)	5.50E+06 (6.740)	-1.85	4.40E+06 6.643	4.80E+06 6.681	-9.09
12	9.45E+06 (6.975)	8.00E+06 (6.903)	15.34	1.02E+07 (7.009)	1.14E+07 (7.055)	-11.27
18	1.04E+07 (7.015)	8.25E+06 (6.916)	20.29	9.80E+06 (6.991)	1.46E+07 (7.164)	-48.98
24	1.67E+07 (7.221)	1.31E+07 (7.116)	21.62	2.50E+07 (7.397)	2.57E+07 (7.409)	-2.81

*=The values in parenthesis indicate the converted value of number of viable cells (CFU/mL) into log_{10} CFU/mL.

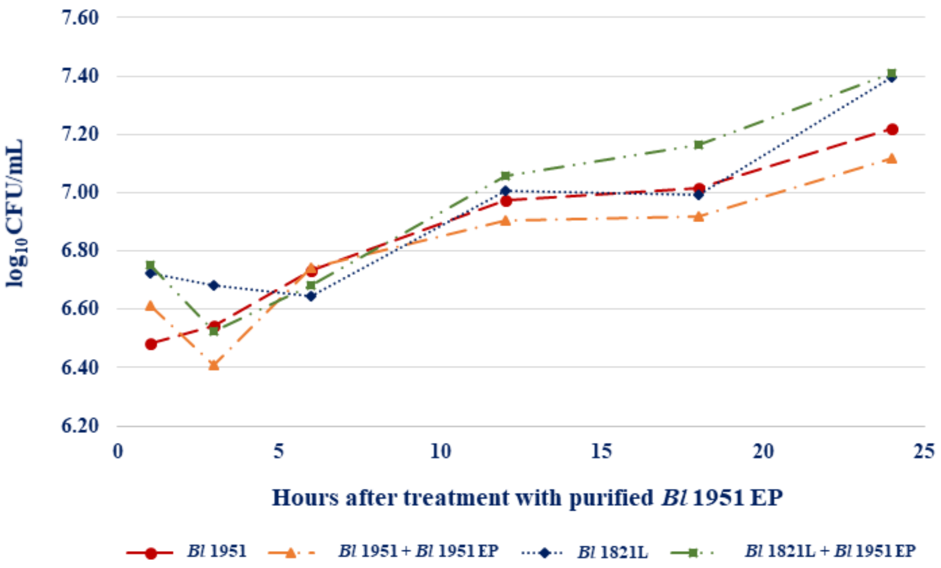

Fig. S17 Number of viable cells ($\log_{10}$ CFU/mL) of *Bl* 1951 and *Bl* 1821L with/without treatment of purified *Bl* 1951 putative encapsulating protein (~30 kDa) after incubation at 30°Cfor various time intervals

Table. S10 Effect of purified *Bl* 1951 ~30 kDa putative encapsulating protein (EP) on the OD_{600nm} reading of *Bl* 1951 and *Bl* 1821L after incubation at 30°C for various time intervals. Data presents the mean value of one experiment

**Time intervals (Hours)**	***Bl* 1951**	***Bl* 1951 + *Bl* 1951 EP**	**% Decrease/increase in OD_{600nm} reading**	***Bl* 1821l**	***Bl* 1821L + *Bl* 1951 EP**	**% Decrease/increase in OD_{600nm} reading**
1	1.76	1.78	-1.42	1.98	1.96	0.76
3	1.68	1.68	0.00	1.92	1.95	-1.30
6	1.58	1.555	1.27	1.91	1.87	1.84
12	1.03	1.01	1.94	1.41	1.33	6.03
18	1.04	0.96	7.69	1.37	1.32	3.30
24	1.30	1.25	3.85	1.66	1.55	6.63
**% Decrease from the start (1 hour) to the end (24 hours) of incubation**	26.2%	29.8%		16.2%	20.9%	

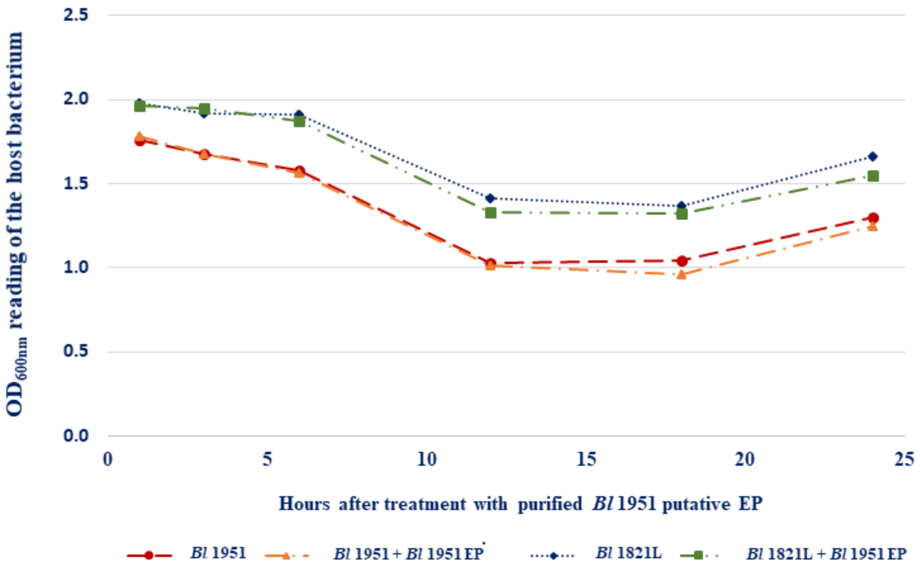

Fig. S18 Effect of *Bl* 1951~30 kDa purified putative encapsulating protein (EP) on the OD_{600nm} reading of *Bl* 1951 and *Bl* 1821L after incubation at 30°C for various time intervals

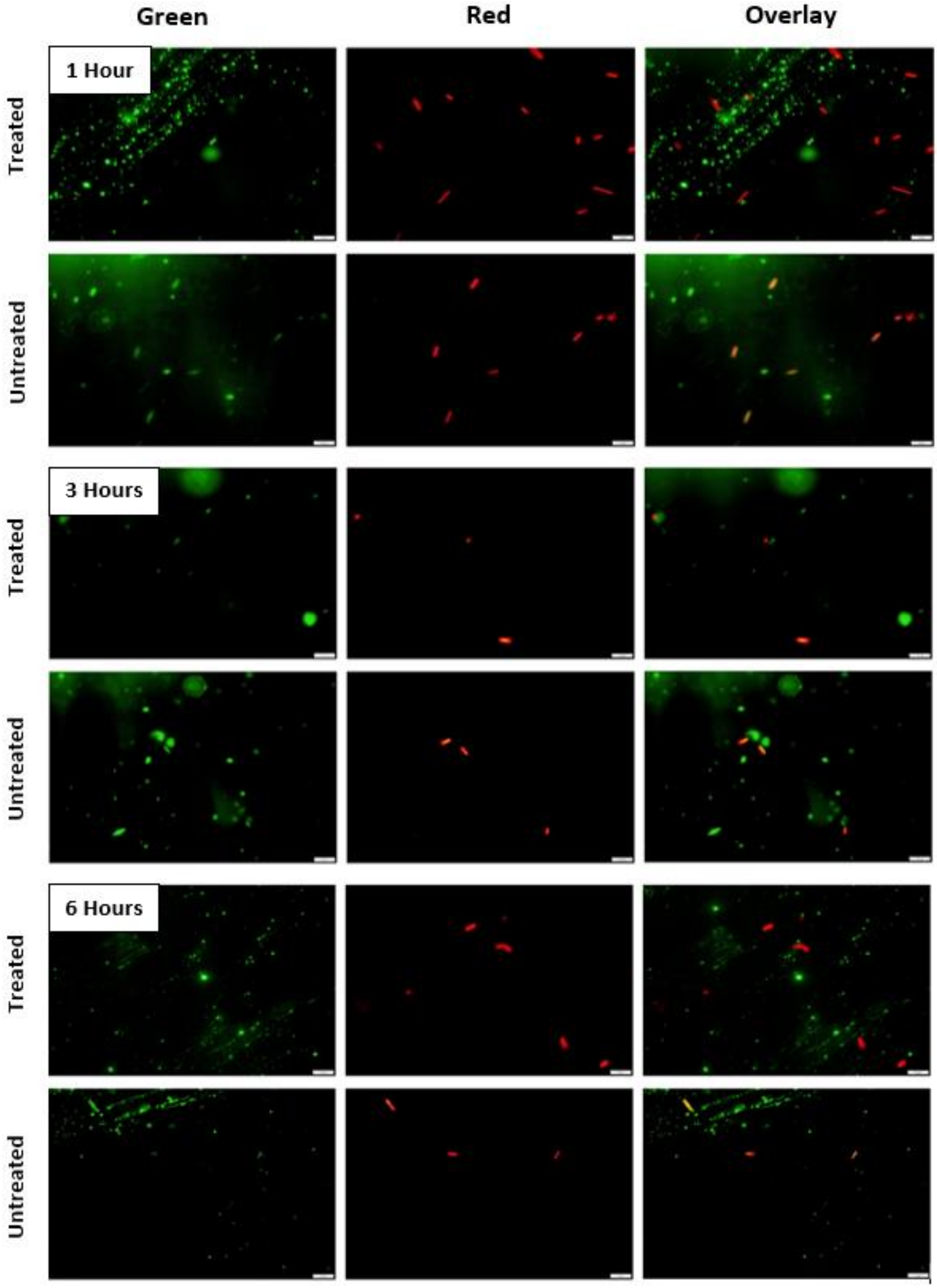
Green
Red
Overlay
1 Hour
Treated
Untreated
3 Hours
Treated
Untreated
6 Hours
Treated
Untreated

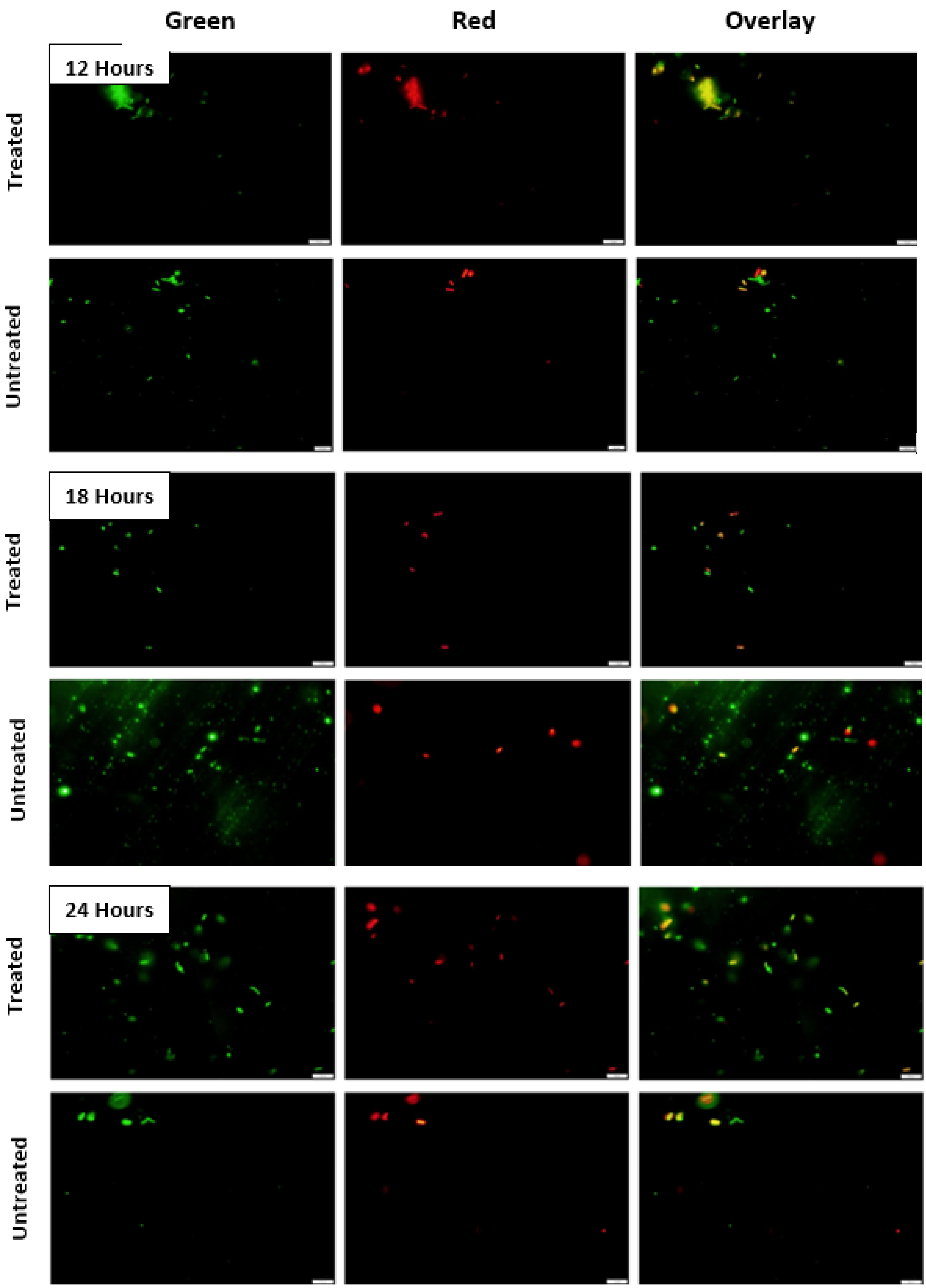

Fig. S19 LIVE/DEAD staining of *Bl* 1821L cells after treatment with the purified *Bl* 1821L putative encapsulating protein (~30 kDa) at a higher volume. Green denotes the live cells while red and orange shows the cells with compromised cell membranes. Scale= 10 µm

Table. S11 Assay of *Bs* WB800N supernatant containing 25 kDa hypothetical (pHT01-*hypo*), 31.4 kDa putative encapsulating (pHT01-*encap*), and both 25 kDa and 31.4 kDa proteins (pHT01-*hypo*.*encap*) against *Bl* 1951 as the host bacterium

Cell free supernatants (CFS)	Zone of inhibition diameter (mm)			
	Time after induction (3.5 Hours)		Time after induction (24 Hours)	
	Induced	Uninduced	Induced	Uninduced
Hypothetical protein (pHT01-*hypo*, A1)	11.7	12.0	-*	-
Hypothetical protein (pHT01-*hypo*, A2)	11.0	-	-	-
Encapsulating protein (pHT01-*encap*, B1)	10.3	10.0	11.0	-
Encapsulating protein (pHT01-*encap*, B2)	-	-	-	-
Hypothetical and encapsulating proteins (pHT01-*hypo.encap*)	10.0	-	12.3	-
** Non- transformants	-	-	-	-

*= No zone of inhibition
**= Non- transformants mean, cell free supernatant of *Bs* WB800N without (pHT01-*hypo*, pHT01-*encap*, pHT01- *hypo*.*enca*p) expression was used against *Bl* 1951as the host bacterium

Table. S12 Assay of *Bs* WB800N supernatant containing 25 kDa hypothetical (pHT01-*hypo*), 31.4 kDa putative encapsulating (pHT01-*encap*), and both 25 kDa and 31.4 kDa proteins (pHT01-*hypo*.*encap*) against *Bl* 1821L as the host bacterium.

**Cell free supernatants (CFS)**	**Zone of inhibition diameter (mm)**			
	**Time after induction (3.5 Hours)**		**Time after induction (24 Hours)**	
	**Induced**	**Uninduced**	**Induced**	**Uninduced**
Hypothetical protein (pHT01-*hypo*, A1)	-*	-	-	9.0
Hypothetical protein (pHT01-*hypo*, A2)	11.7	-	9.7	-
Encapsulating protein (pHT01-*encap*, B1)	-	10.3	12.3	11.7
Encapsulating protein (pHT01-*encap*, B2)	-	-	11.0	-
Hypothetical and encapsulating proteins (pHT01-*hypo.encap*)	9.3	-	11.0	-
** Non-transformants	-	-	-	-

*= No zone of inhibition
**= Non- transformants mean, cell free supernatant of *Bs* WB800N without (pHT01-*hypo*, pHT01-*encap*, pHT01- *hypo*.*enca*p) expression was used against *Bl* 1821L as the host bacterium

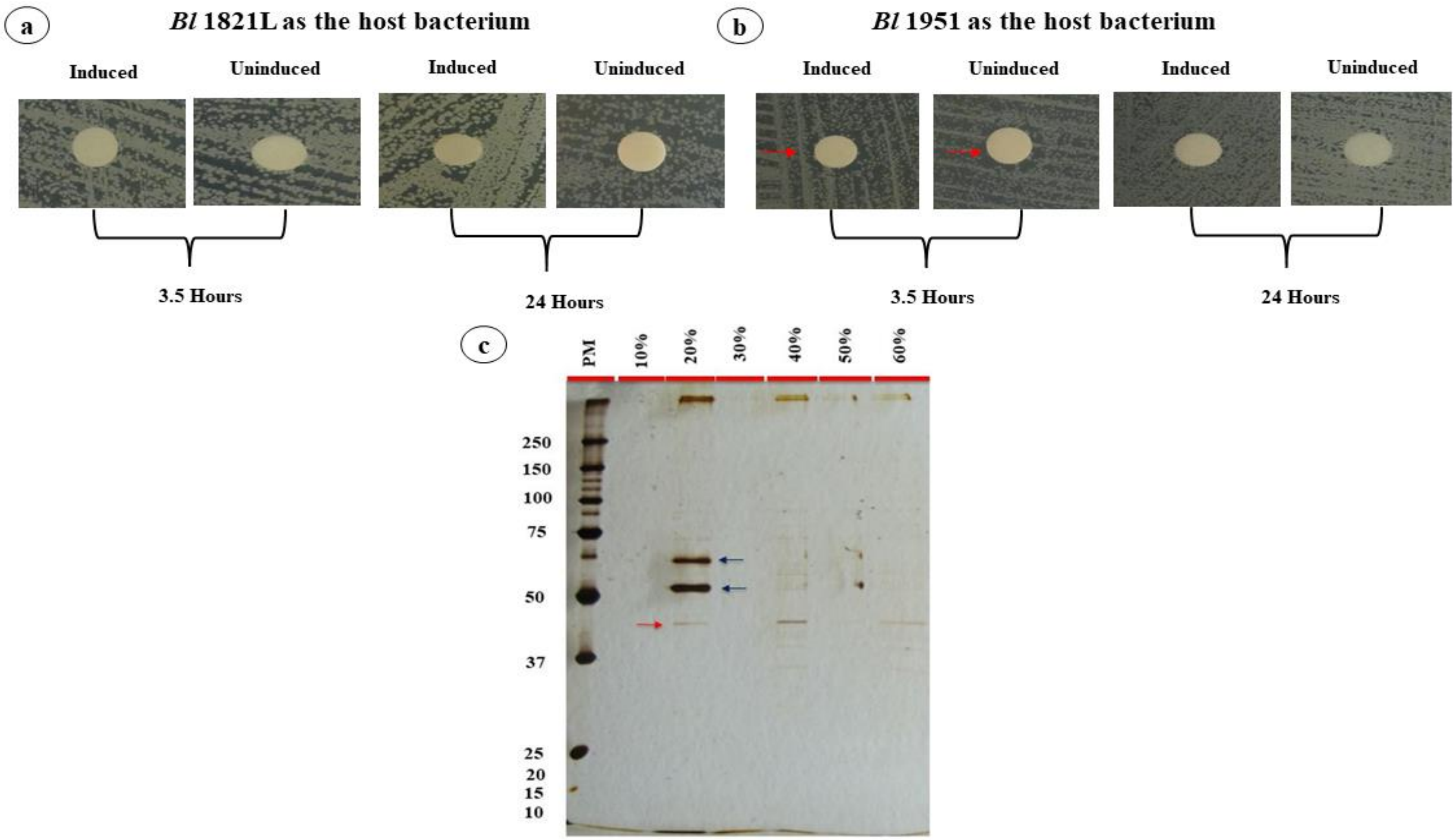

Fig. S20 Assay test of CFS from *Bs* WB800N (pHT01-*hypo*, A1) expressing 25 kDa hypothetical protein against *Bl* 1821L (Fig. S20a) and *Bl* 1951 (Fig. S20b) as the host bacterium. Arrows (red) denote the zones of inhibition showing a diameter of ≥11 mm. SDS-PAGE analysis of supernatant from *Bs* WB800N (pHT01-*hypo*, A1) expressing 25 kDa hypothetical protein after 3.5 hours of induction (Fig. S20c). The red arrows denote sucrose density gradient purified proteins of ~48 kDa and dark blue arrows denote purified proteins of ≥50 kDa (Fig. S20c)

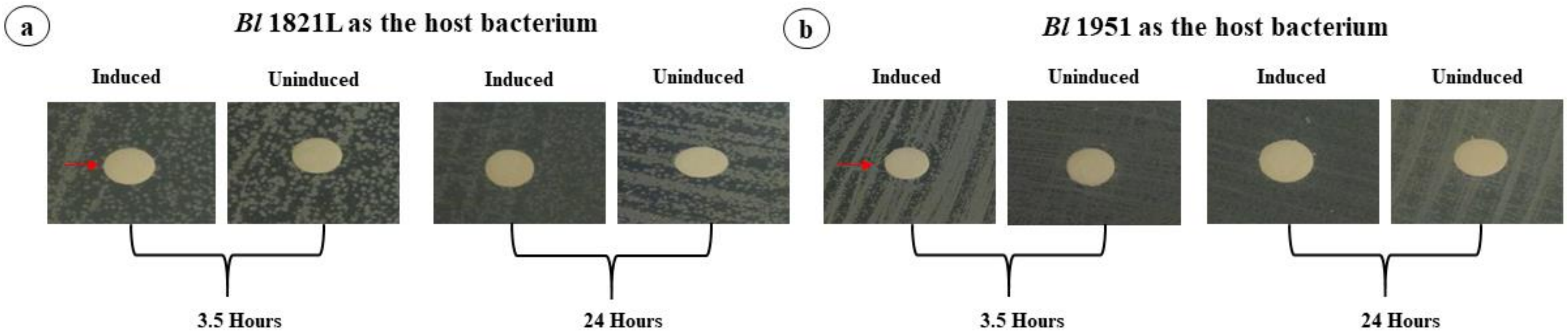

Fig. S21 Assay test of CFS from *Bs* WB800N (pHT01-*hypo*, A2) expressing 25 kDa hypothetical protein against *Bl* 1821L (Fig. S21a) and *Bl* 1951 (Fig. S21b) as the host bacterium. Arrows (red) denote the zones of inhibition showing a diameter of $\geq$11 mm

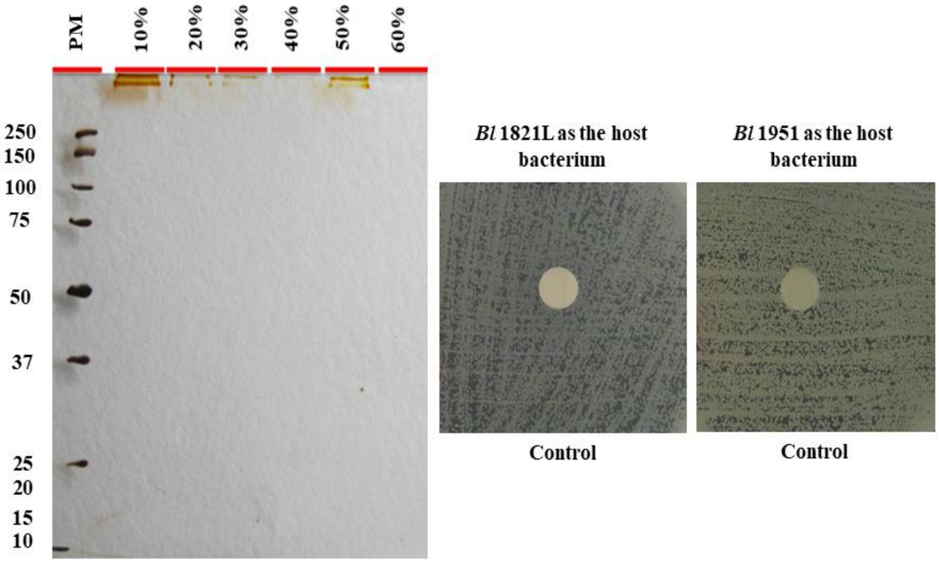

Fig. S22 SDS-PAGE analysis of *Bs* WB800N concentrated CFS protein without any hypothetical (25 kDa) and putative encapsulating (31.4 kDa) protein expression (left side image). Assay tests of CFS of *Bs* WB800N without transformation (pHT01-*hypo*, pHT01-*encap*, pHT01-*hyo.encap*) against *Bl* 1821L and *Bl* 1951 as the host bacterium (right side image)

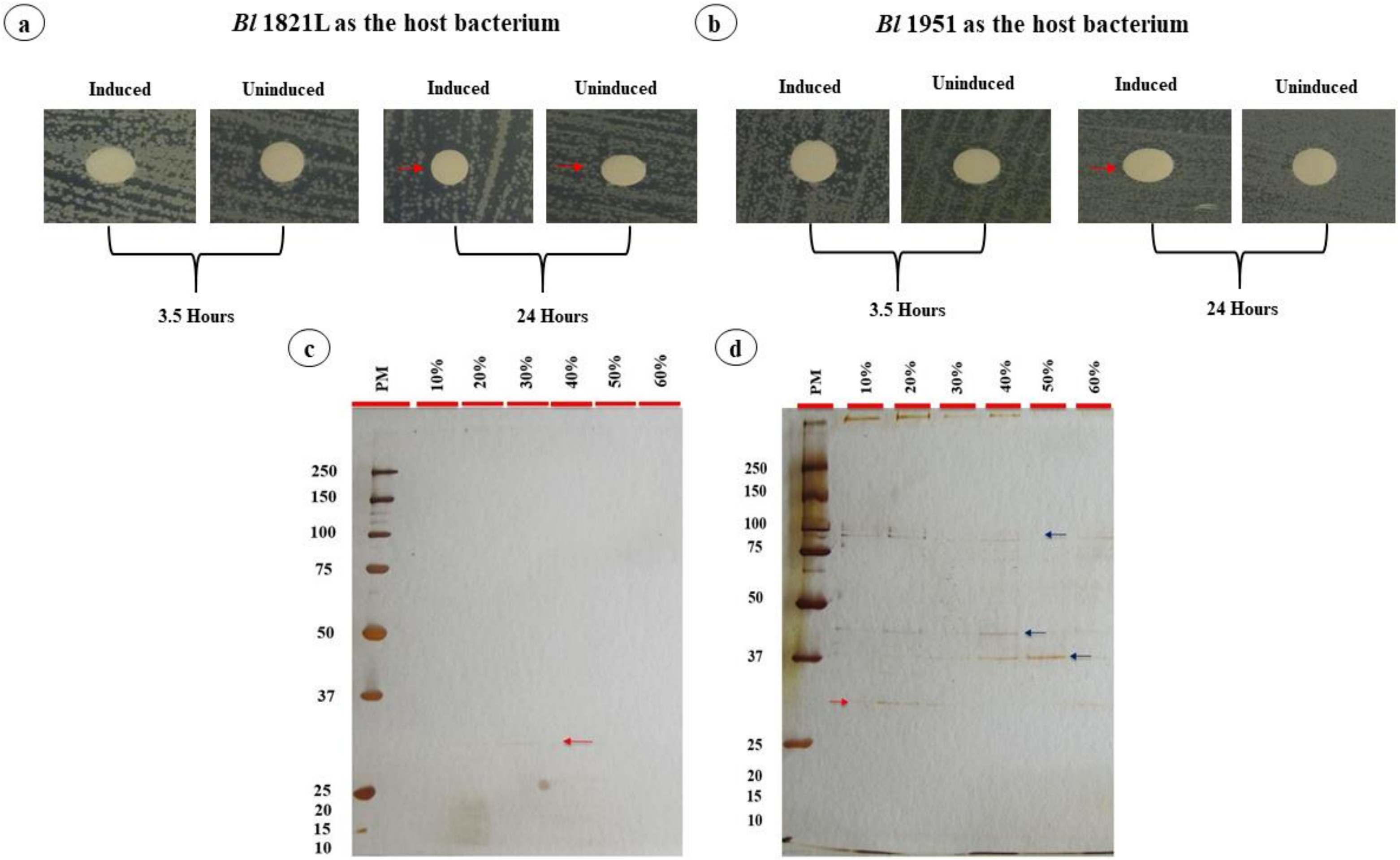

Fig. S23 Assay test of CFS from *Bs* WB800N (pHT01-*encap*, B1) expressing 31.4 kDa putative encapsulating protein against *Bl* 1821L (Fig. S23a) and *Bl* 1951 (Fig. S23b) as the host bacterium. Arrows (red) denote the zones of inhibition showing a diameter of ≥11 mm. SDS-PAGE analysis of 31.4 kDa putative encapsulating protein expressed after 3.5 hours (Fig. S23c) and 24 hours (Fig. S23d) of induction from *Bs* WB800N (pHT01-*encap*, B1). Arrows denote sucrose density gradient purified ~30 kDa encapsulating protein (red) and other proteins (dark blue)
